# Self-Organizing Maps: An AI Tool for Identifying Unexpected Source Signatures in Non-Target Screening Analysis of Urban Wastewater by HPLC-HRMS

**DOI:** 10.3390/toxics12020113

**Published:** 2024-01-29

**Authors:** Vito Gelao, Stefano Fornasaro, Sara C. Briguglio, Michele Mattiussi, Stefano De Martin, Aleksander M. Astel, Pierluigi Barbieri, Sabina Licen

**Affiliations:** 1 Regional Environmental Protection Agency—ARPA-FVG, Via Cairoli 14, 33057 Palmanova, Italy; vito.gelao@arpa.fvg.it (V.G.); sara.briguglio@arpa.fvg.it (S.C.B.); michelmatt@alice.it (M.M.); stefano.demartin@arpa.fvg.it (S.D.M.); 2 Department of Chemical and Pharmaceutical Sciences, University of Trieste, Via Giorgieri 1, 34127 Trieste, Italy; sfornasaro@units.it (S.F.); barbierp@units.it (P.B.); 3 Department of Environmental Chemistry and Toxicology, Pomeranian University in Słupsk, 22a Arciszewskiego Str., 76-200 Słupsk, Poland

**Keywords:** principal component analysis, hierarchical clustering analysis, SOM

## Abstract

(1) Background: Monitoring effluent in water treatment plants has a key role in identifying potential pollutants that might be released into the environment. A non-target analysis approach can be used for identifying unknown substances and source-specific multipollutant signatures. (2) Methods: Urban and industrial wastewater effluent were analyzed by HPLC-HRMS for non-target analysis. The anomalous infiltration of industrial wastewater into urban wastewater was investigated by analyzing the mass spectra data of “unknown common” compounds using principal component analysis (PCA) and the Self-Organizing Map (SOM) AI tool. The outcomes of the models were compared. (3) Results: The outlier detection was more straightforward in the SOM model than in the PCA one. The differences among the samples could not be completely perceived in the PCA model. Moreover, since PCA involves the calculation of new variables based on the original experimental ones, it is not possible to reconstruct a chromatogram that displays the recurring patterns in the urban WTP samples. This can be achieved using the SOM outcomes. (4) Conclusions: When comparing a large number of samples, the SOM AI tool is highly efficient in terms of calculation, visualization, and identifying outliers. Interpreting PCA visualization and outlier detection becomes challenging when dealing with a large sample size.

## 1. Introduction

The monitoring of effluent in water treatment plants has a key role in identifying possible pollutants that can be released into the environment. Currently, the concentration level of several micropollutants is regulated; nevertheless, they are a minimal part of the possible substances of health concern that can be present in the effluents among the following categories: pharmaceuticals and their metabolites, personal care products, pesticides, flame retardants, plasticizers, oxidation treatment by-products, etc. [1,2]. A non-target analysis approach can be used to identify unknown substances and isolate multipollutant signatures related to specific sources using HPLC-HRMS methods [3,4,5]. HPLC-HRMS technique focused on non-target screening (NTS) produces a huge amount of data; thus, it is necessary to use multi-step workflows involving multivariate data analysis techniques such as hierarchical clustering (HCA) and principal component analysis (PCA) [6,7,8,9,10] for obtaining interpretable results.

Most of the studies present in the scientific literature aimed to identify source patterns [4,8,9] and significant differences among sites or processes [6,10,11], also considering time/season variations [12,13,14,15].

In some works [15,16,17] focused on HPLC-HRMS pollutant target analysis, the authors used a type of neural network named Self-Organizing Map (SOM) [18,19,20] for unsupervised multivariate analysis, highlighting the benefits of using it instead of PCA or HCA. SOMs are a rather old type of artificial intelligence (AI) that employs unsupervised learning to convert high-dimensional input data into a lower-dimensional grid composed of nodes or neurons. By engaging in a competitive learning process, every node adjusts itself to distinct patterns within the data, allowing for meaningful insight extraction from data [21,22,23,24]. While SOMs are often classified under the umbrella of machine learning (ML), it is important to recognize that ML itself is a subset of AI. AI encompasses a broader spectrum of techniques, including symbolic reasoning, expert systems, and other knowledge-based approaches. Machine learning, on the other hand, specifically focuses on algorithms that enable systems to learn and improve from experience. SOMs can be classified as AI tools because they possess the ability to learn and adapt to the underlying structure of data without explicit programming for certain tasks. This learning capability aligns with the broader goals of AI. Moreover, SOMs excel at extracting patterns and relationships in complex, high-dimensional data. This aligns with the fundamental goals of AI, which include developing systems capable of recognizing and understanding patterns in diverse datasets [25]. SOMs have several advantages over classical chemometrics methods such as PCA and HCA, including the ability to capture non-linear relationships and structure in the data, the ability to deal with complex high-dimensional datasets, meaningful visualization of the outcomes in 2D maps, tolerance, and robustness to outliers, the ease with which the latter can be identified, and the simplicity with which the results can be related to the experimental variable importance. Nevertheless, only one NTS study used the above-mentioned algorithm for resolving ambiguities in homologs identification in sewage treatment plant effluents [26].

As a consequence, to fill the gap in scientific evidence, this study aims to highlight the capability of the SOM algorithm in identifying industrial wastewater effluent signatures in urban wastewater as outliers, only focusing on “unknown common” compounds. The objective is to build a model that can detect the presence of industrial wastewater in urban wastewater, even when the industrial source profiles are unknown and the wastewater is diluted. The results were compared to those obtained using PCA, showing the benefits of using the SOM tool.

## 2. Materials and Methods

### 2.1. Reagents

LC-MS grade formic acid, acetonitrile (ACN), methanol (MeOH), and ammonium formate were provided by Carlo Erba (Cornaredo, MI, Italy). Water was obtained by a MilliQ Water System (Millipore, Burlington, MA, USA). The list of standard compounds of pesticides, perfluoroalkyl compounds, and pharmaceutical products is reported in Table S1 of the Supplementary Materials. Atrazine-D5 (100 mg/L in acetone) was purchased by Dr. Ehrenstorfer GmbH (Augsburg, Germany). Calibration solutions (“Pierce LTQ Velos ESI Positive Ion” and “Pierce ESI Negative Ion”) for the Orbitrap mass spectrometer were purchased from Thermo Scientific (Waltham, MA, USA).

### 2.2. Solutions

A multi-standard solution (237 compounds) was obtained by diluting the stock solutions to 10 µg/L (for each analyte) in MeOH and then to 100 ng/L in water. The atrazine-D5 solution was used as an internal standard and obtained by dilution of the 100 mg/L stock solution to 10 µg/L in ACN.

Mobile phases for liquid chromatography were the following: (A) water containing 5 mM formic acid and 5 mM ammonium formate; (B) MeOH.

### 2.3. Sample Collection and Processing

Water samples were collected from the effluent discharge point of ten WWTPs located in Friuli Venezia-Giulia region (Italy). Six were urban WWTPs, and four were industrial WWTPs. The exact locations are not provided to keep the identities of the WWTPs confidential. From now on, the urban WWTPs will be referred to as U1 to U6, and the industrial ones will be referred to as I1 to I4. The water samples (500 mL) were collected by grab sampling in amber glass bottles. The samples were collected in October 2021. After collection, the samples were stored at 4 °C until the analysis was performed within a week from the sampling.

Aiming to minimize sample handling, the samples were transferred in a 2 mL vial and centrifuged at 13,000× *g* (relative centrifuge force—rcf) for 5 min with a Centrifuge 5415D (Eppendorf, Amburg, Germany). The supernatant was then transferred to a 1 mL vial suitable for autosampling. A total of 10 µL of internal standard atrazine-D5 solution was added to the samples before analysis. The blank samples were prepared using water added to the internal standard solution. The internal standard was added to the multi-standard solution as well.

For each site, two replicates were analyzed (from now on defined in the text as “concentrated”) together with two diluted replicates (1:10 in water); thus, four samples were analyzed for each site. Considering the replicates and the dilution level, the samples will be named in the text as follows: site name (e.g., U1), dilution level (c or d for concentrated or diluted), and replicate number (1 or 2), and thus, e.g., the first replicate of the concentrated sample collected in U1 will be named as U1c1. The sequence of analysis used for each sampling site was two blanks, diluted samples, and concentrated samples. The same sequence was used for the multi-standard solution.

### 2.4. Sample Analysis

The analysis was carried out using a UHPLC (Dionex Ultimate 3000, Thermo Fisher Scientific, Waltham, MA, USA) equipped with an autosampler (PAL, CTC Analytics, Zwingen, Switzerland) and coupled to a mass spectrometer Orbitrap Q Exactive quadrupole Focus (Thermo Fischer Scientific, Waltham, MA, USA). The UHPLC was equipped with an Ascentis Express C18 column (150 × 2.1 mm, 2.7 µm, Supelco, Sigma-Aldrich, St. Louis, MO, USA) maintained at 35 °C. Column flow was set at 0.3 mL/min. Gradient elution started with 2% B for 2 min, changed to 98% B in 20 min, and then 98% B was kept for 6 min. Eventually, the mobile phase composition was changed to the starting conditions and kept for 4 min. The measurements were acquired in negative and positive ionization in the full scan data-dependent mode (ddMS2) in the *m*/*z* 50–1000 Da range. Details about the mass spectrometer settings are reported in Table S2. Xcalibur™ version 3.0 (Thermo Scientific, Waltham, MA, USA) and Chromeleon™ version 6.8 (Thermo Scientific, Waltham, MA, USA) software were used for instrument control.

### 2.5. Untargeted Analysis

The mass spectra were elaborated on using Compound Discoverer (version 3.2) software (Thermo Scientific, Waltham, MA, USA). Details about the untargeted analysis workflow are reported in Figure S1.

#### Annotation Filtering and Manual Supervision

The compound list obtained using the untargeted analysis workflow was further filtered for undesired features. The compounds that showed a peak area less than five times the area of the same compound present in the blanks were marked as background and discarded from the list. Moreover, the differences in the compound areas among the samples and the respective diluted ones were checked using *t*-test. Compounds that showed no statistical difference (*p*-value > 0.05) between the above-mentioned groups, i.e., that did not show a correlation between area and concentration, were discarded.

The QSRR model proposed by Aalizadeh et al. [27] has been used for annotation retention time prediction and comparison to the measured retention time. The QSRR calibration curve has been built using the retention time of the compounds present in the multi-standard mixture.

Eventually, the compounds were identified according to the confidence level list proposed by Schymanski et al. [28]. Level 1 has been assigned to compounds identified using the multi-standard mixture. Level 2 has been assigned to compounds that satisfy both the following conditions: a match above 75% with a database mass spectrum and a recorded retention time within 4 min concerning the modeled one. Level 3 has been assigned to first labeled “level 2” compounds that did not pass the manual supervision of the matching of the fragmentation spectra with the database (e.g., fragmentations spectra with very few peaks). Level 4 has been assigned to compounds that were identified both by an exact mass and an unequivocal molecular formula, and level 5 has been assigned to compounds identified by an exact mass only.

### 2.6. Multivariate Data Analysis

The dataset obtained by the previous steps has been handled in R software environment (version 4.0.5) [29]. The “unknown common” compound selection has been obtained by hierarchical cluster analysis (HCA) using the pheatmap package [30]. Principal component analysis (PCA) has been performed using the mdatools package [31]. Self-Organizing Map analysis has been performed by SOMEnv package [32].

## 3. Results and Discussion

The elaboration of the mass spectra described in par. 2.5 allowed us to identify, at different levels of confidence, 12,419 compounds overall. The obtained dataset was composed of 40 rows (considering the replicates and the diluted samples for each sampling site).

The dataset elaboration has been performed according to the following steps:

- Screening of the compounds for identifying the artifacts (the features that showed a greater peak area in the diluted sample than in the concentrated one have been discarded);
- Identification by HCA of the “unknown common” compounds, i.e., the substances common among the industrial and urban samples;
- Splitting of the “unknown common” dataset in industrial samples and urban samples;
- PCA and SOM model building using the urban subset;
- Projection of the industrial subset into the above-mentioned models;
- Comparison of SOM and PCA outcomes.

### 3.1. Screening for Artifacts Identification

The dataset has been centered and scaled by variable to unit variance (z-score transformation). This scaling was implemented to standardize the variances of the variables across various samples and conditions. The transformation was necessary due to the variations in concentrations of the compounds in the dataset, which were in different ranges [33]. The mean of the replicates was evaluated for each compound according to the sampling site and the dilution level. A dummy variable (“Correctness”) describing when the diluted mean was lower than the concentrated one was created and coded as either 1 or 0 to indicate if the condition was fulfilled or not. Thus, for each compound, ten values were obtained (one for each sampling site). The compounds were discarded if the median of the dummy variable was found to be <1. The screening led to retaining 9681 compounds.

### 3.2. Selection of “Unknown Common” Compounds

**Figure 1:**
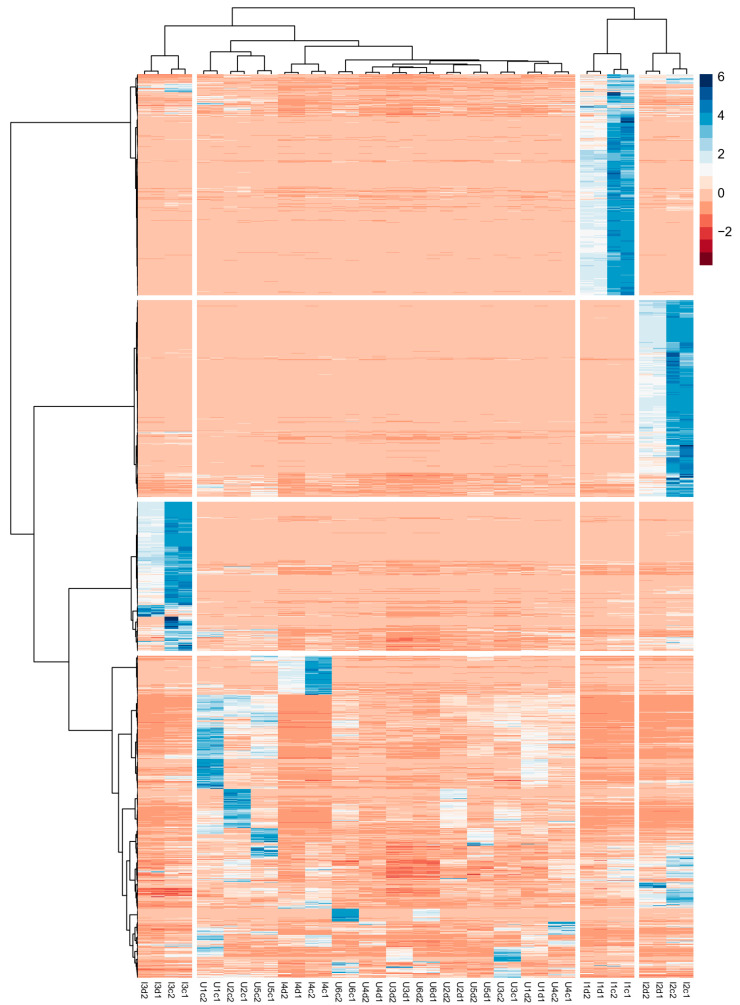
A two-way clustering heatmap was obtained by applying HCA to both the samples (depicted column-wise) and the variables (depicted row-wise; the labels are omitted for the sake of the readability of the figure). The color scale represents the z-score normalization values.

HCA was applied to the 40 × 9681 dataset using the Euclidean distance and Ward’s linkage method. HCA was applied to both the samples and the variables to obtain a so-called two-way clustering heatmap, as shown in Figure 1.

The heatmap discloses that the sites I1, I2, and I3 show a set of peculiar compounds that can be identified as separated clusters by examining Figure 1 from the top. The compounds grouped in the cluster depicted at the bottom of Figure 1 were considered “unknown commons”, while the former were discarded from the dataset. The number of “unknown common” compounds was 3507. The “unknown common” dataset (40 × 3507) has been split into two subsets: the urban subset (24 × 3507) and the industrial subset (16 × 3507). These subsets were used in the subsequent steps. The list of “unknown common” compounds reports the confidence level of identification according to the method described in par. 2.4 is reported in Table S3. The list of “unknown common” compounds is reported in Table S3. The confidence level of identification for each compound is reported based on the method described in Section 2.5. There were 10 compounds identified at level 1, 104 at level 2, 78 at level 3, 3097 at level 4, and 218 at level 5. Even if a higher level of identification is often preferable, it would require additional MS/MS experiments, which were out of the scope of the presented study. It should be noted that the confidence level of identification has no impact on the subsequent data analysis.

### 3.3. PCA Model

**Figure 2:**
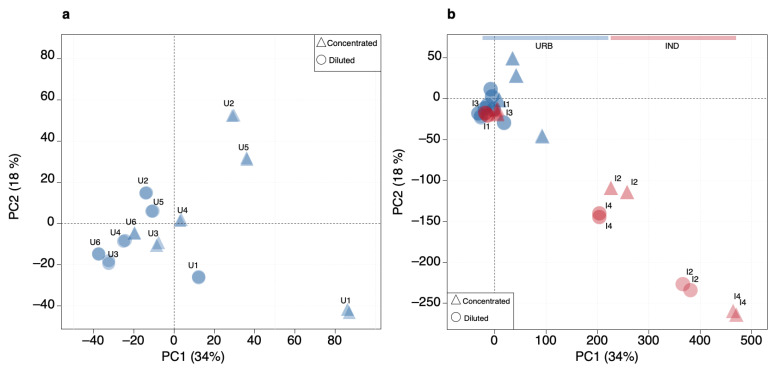
PCA score plots (PC1, explaining 34% of the total variance, vs. PC2, explaining 18% of total variance) of the urban dataset (URB) (**a**) and including the projection of industrial samples (IND) (**b**).

Principal component analysis (PCA) was employed for the initial exploration of the urban data, thus defining the baseline condition (Figure 2a). Clusters formed by the concentration profiles of “unknown common” compounds can be recognized in the PCA score plot of the first two principal components (PCs). Technical replicates were always grouped together. However, a clear separation between concentrated and diluted samples was not evident. More interestingly, when the industrial samples were projected onto the same PC space (Figure 2b), most of them deviated significantly from the origin, but two samples (I3 and I1) appeared well within the cloud of urban samples, showing a similar pattern.

**Figure 3:**
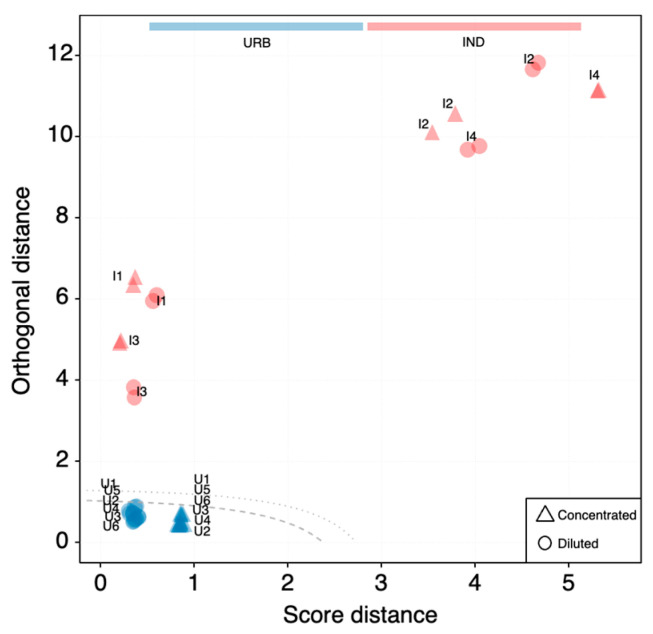
Distance–distance plot of the PCA model with PCs = 6, α = 0.05, γ = 0.05. Axes are logarithmically converted for illustration purposes. The lines on the distance plot represent critical limits for extreme objects (dashed) and outliers (dotted) at α = 0.05.

The detection of the anomaly of the new incoming industrial data was then revealed by visual analysis of score distance (SD) and orthogonal distance (OD) from the centroid of the covariance data structure [34]. The number of principal components to retain was selected using Kaiser’s rule and by looking at the explained variance scree plot. The distance–distance plot constructed for the PCA model with 6 PCs is shown in Figure 3. Samples are placed in accordance with their SD and OD values. In general, samples with high SD values are close to the PCA space but far from the model center, and samples with high OD values are far from the PCA space with large residuals, but their projection may be within the model. The dashed line represents the critical limit for extreme objects (defined by a significance level α, set to 0.05); the dotted line represents the critical limit for outliers (defined by the significance level γ, set to 0.05). The two lines can be used to decide whether a sample should be considered as part of the urban samples’ population or not. All the industrial samples (in red) are easily identified as outlying samples, while the small dissimilarity between the concentrated (triangles) and diluted (circles) urban samples is easily recognized along the SD axis despite remaining within the critical limits. By interpreting these results with respect to mass spectrometry fingerprinting, it can be said that higher SD values reflect different intensity levels of the same chemical characteristics. Industrial samples with both high OD and SD values deviate in the existence and ratios of chemical compounds (overall pattern) from the urban baseline characteristics. Interestingly, industrial samples I1 and I3, which were clustered together within the same cloud of all urban samples in the score plot of Figure 2b, had more or less the same SD values as the diluted urban samples but with much higher OD values.

### 3.4. SOM Model

The urban dataset was used for building the SOM model. The number of nodes on the map has to be chosen by the user; some heuristic rules are available. Usually, more than one map is trained, selecting different numbers of nodes and map dimension ratio and checking the quality of the model. Typically, the SOM map dimensions are a compromise between preserving the topology of the dataset and the multivariate distance between the nodes and the samples. The model can be evaluated using some quality parameters: TE (topographic error), overall QE (quantization error), distribution matching error (DME), and number of nodes not representing any sample (i.e., number of nodes without “hits”). The values of TE and QE should be minimized, but they typically have an inverse relationship with the number of nodes. However, there are no specific reference values. The DME should also be minimized to mitigate any distortion in the fitting of variables in the model with respect to the dataset. It is advisable to avoid having a large number of nodes without hits [20,35]. We trained two maps according to Vesanto’s heuristic rules [24], thus considering a map dimension ratio as close as possible to the square root of the ratio between the first two eigenvalues of the dataset. The number of nodes in each map was determined based on two options: either five times the square root of the number of samples (referred to as a “regular” map) or 5/4 times the square root of the number of samples (referred to as a “small” map). After evaluating the recommended quality parameters for each map, we made the decision to create two additional maps, considering a number of nodes between the “small” and “regular” map and retaining a dimension ratio as close as possible to the square root of the ratio between the first two eigenvalues, which had a value of 1.4. The results are summarized in Table 1.

**Figure 4:**
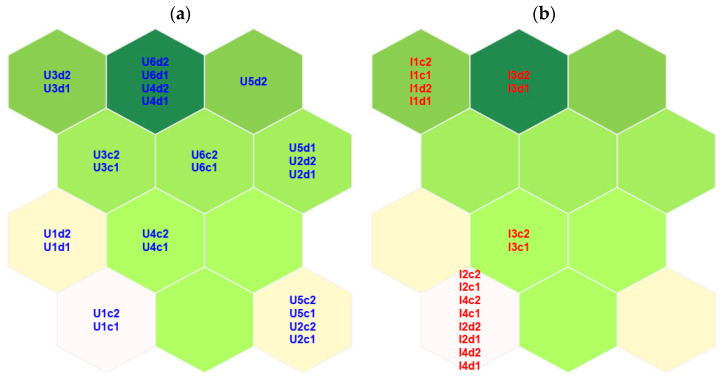
SOM model representation by 2D hexagonal maps. (**a**) The urban samples are depicted in blue on the map. (**b**) The projected industrial samples are depicted in red on the map. The Euclidean distance among the nodes is depicted from green to white for increasing values.

The trade-off among the quality parameter results led us to choose the 4 × 3 map, thus composed of 12 nodes. Each node is a vector containing a modeled value for each experimental variable and can be interpreted as a recurrent signature commonly found in urban WTPs. The nodes are depicted as hexagons in a 2D map shown in Figure 4. The Euclidean distance among the nodes is depicted from green to white for increasing values. Thus, for example, the node in the bottom left of the map is less similar with respect to its neighbors, and the dark green node on the top of the map is more similar to its neighbors. In the map represented in Figure 4a, the distribution of the urban samples on the map is shown. The samples are depicted on the node that is less distant (Euclidean distance) from the sample; the assigned node is called the best matching unit (BMU). Thus, the samples that are more different from the others are U1 (both replicates and diluted ones) and U2 with U5 (not diluted samples only for both sites).

Considering the SOM model as a list of signatures commonly found in urban WTPs, the industrial samples were then projected into the model to detect possible differences, obtaining the distribution shown in Figure 4b. When data not used to build the model are projected onto the map, they are assigned to the less distant node (their BMU). It can be observed that the signatures of I2 and I4 are considered similar to those of concentrated U1 samples, and concentrated I3 is similar to concentrated U4, diluted I3 is similar to diluted U4, and I1 is similar to concentrated U3. Nevertheless, the degree of similarity has to be quantitatively evaluated as follows: the distance between a sample and its BMU is called “quantization error” (QE) and can be calculated both for samples used for SOM model building and for projected ones. Samples showing high QE values are considered possible outliers [36,37,38].

**Figure 5:**
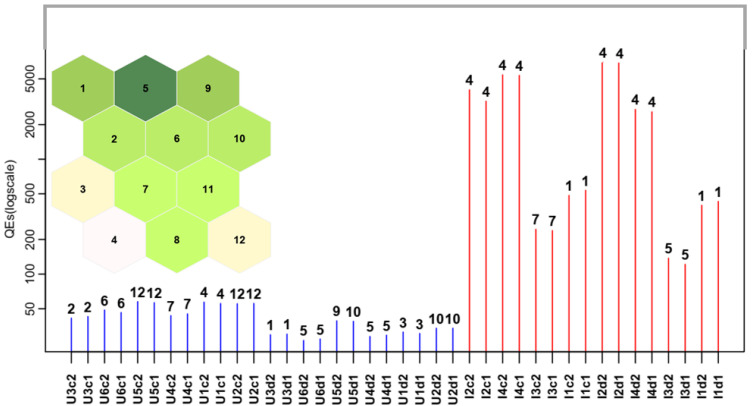
Quantization error (QE—logscale) plot for each sample. Urban samples are in blue, while industrial ones are in red. The corresponding Best Matching Unit (BMU) number is shown on the top of each bar. The BMU number code is shown in the SOM map and is represented in the top left part of the figure. In the map, the Euclidean distance among the nodes is depicted from green to white for increasing values.

In Figure 5, the QEs (logscale), together with the BMU assignment both for urban and industrial samples, are reported. The BMU number code is shown in the map and is represented in the top left of the plot. It can be observed that the industrial samples show very high values of QEs, even the diluted ones. Thus, their identification as outliers is very easy.

**Figure 6:**
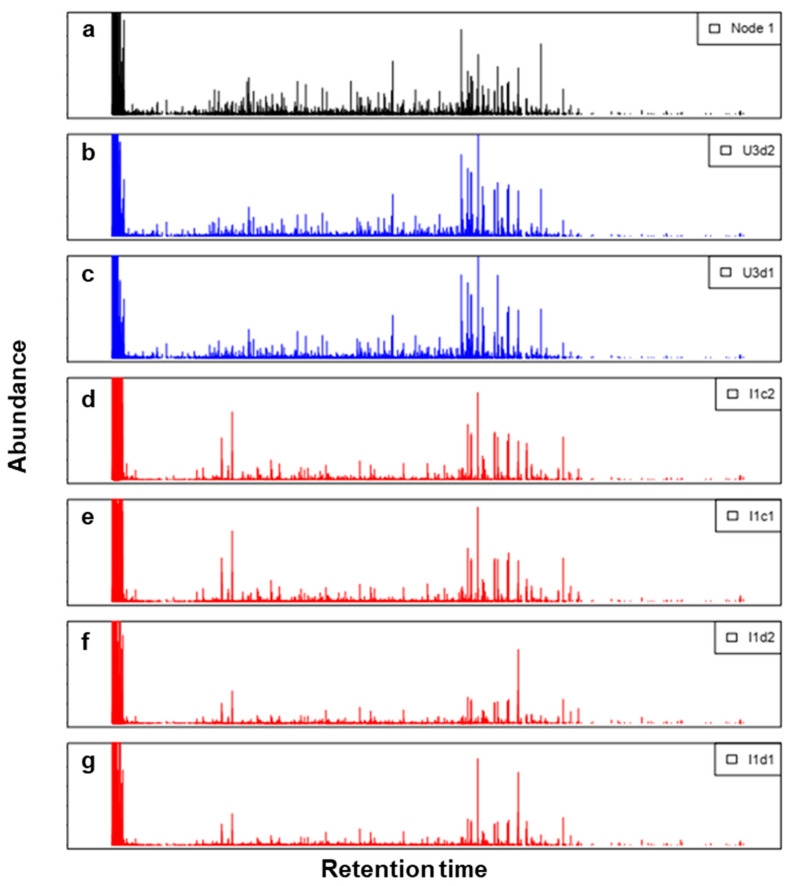
Stacked plots represent the following: (**a**) Node 1 signature recreated as a chromatogram (black). (**b**,**c**) Experimental urban signatures are assigned to that node (blue). (**d**–**g**) Industrial signatures are projected in that node (red).

Considering that the SOM model retains the experimental variables without creating new combined variables (as in PCA), the node signatures can be easily displayed as chromatograms. In Figure 6, an example is presented (the plots for the other nodes are reported in the Supplementary Materials). On the top, the Node 1 signature is recreated as a chromatogram. Below it, the experimental signatures assigned to that node, both urban and industrial ones, are represented. By solely examining the chromatograms, it is evident that there are discernible distinctions between the industrial and urban samples. However, accurately categorizing the extent of the overall dissimilarity is challenging. Using the QEs shown in Figure 5, the industrial samples can easily be detected as “very different” from the urban ones.

### 3.5. SOM and PCA Outcome Comparison

SOM and PCA models led to similar results in terms of sample grouping and outlier detection, but SOM model features allowed us to obtain more insight and useful information from the data. First of all, the outlier detection was very straightforward in the SOM model due to the presence of QEs in the usual output list of outcomes [20]. In contrast, for PCA, the distances presented in Figure 2 have to be calculated, and the appropriate number of PCs has to be selected. Although 2 PCs explained 52% of the variance, the difference among the samples could not be completely discerned (Figure 2b). Six PCs were necessary (Figure 3), but they cannot be depicted in a 2D plot. Moreover, as in PCA, new variables are calculated from the experimental ones, and it is impossible to reconstruct a chromatogram that displays the recurrent signatures in the urban WTP samples. However, this can be achieved using the SOM outcomes (Figure 6). Given a scenario where hundreds of samples need to be compared, the SOM model is easily able to deal with them in terms of computation, visualization, and outlier detection. In that situation, it can be very difficult to interpret both PCA visualization and outlier detection.

## 4. Conclusions

In this paper, we reported the first use of the SOM for the identification of industrial wastewater intrusion in urban wastewater treatment plants, building a model only considering recurrent urban wastewater “unknown common” compound signatures. The above-mentioned AI tool enabled us to explore a complex mass spectra dataset obtained by HRMS and detect outliers without an a priori knowledge of industrial signatures, outperforming most commonly used tools such as PCA. Moreover, the presented method does not require a high level of confidence in compound identification to be effective. Finally, it should be noted that the presence of diluted samples could not deceive the SOM tool into detecting industrial infiltration.

## Figures and Tables

**Table 1:** List of trained SOM models and their quality parameters. The chosen model is reported in bold.

Map Size	Dimension Ratio	Nodes without Hits	DME	QE	TE
6 × 4 (“regular”)	1.5	13 (54%)	0	32.8	0
4 × 2 (“small”)	2.0	0 (0%)	0	43.9	0
5 × 3	1.7	4 (27%)	0	38.9	0
**4 × 3**	**1.3**	**2 (17%)**	**0**	**41.6**	**0**

## Data Availability

The row data will be made available on request.

## Supplementary Materials

The following supporting information can be downloaded at https://www.mdpi.com/article/10.3390/toxics12020113/s1. Table S1: List of standards; Table S2: Mass spectrometer settings; Figure S1: Untargeted analysis workflow; Table S3: “unknown common” compound list; Figure S2: Comparison of the node and sample signatures [39,40].
