# Supplementary material for: Self-Organizing Maps: An AI Tool for Identifying Unexpected Source Signatures in Non-Target Screening Analysis of Urban Wastewater by HPLC-HRMS
Source: Toxics. 2024 Jan 29;12(2):113. doi: 10.3390/toxics12020113 (PMC10891566; doi:10.3390/toxics12020113)
Supplement: Supplementary file 1 [file toxics-12-00113-s001.zip › toxics-2803773-supplementary.pdf]

SUPPLEMENTARY MATERIAL

Self-Organizing Maps: an AI tool for identifying unexpected source signatures in urban wastewater non-target screening analysis by HPLC-HRMS

Vito Gelao [1], Stefano Fornasaro [2], Sara C. Briguglio [1], Michele Mattiussi [1], Stefano De Martin [1], Aleksander M. Astel [3], Pierluigi Barbieri [2] and Sabina Licen [2,*]

Table S1: List of standards
Table S2: Mass spectrometer settings
Figure S1: Untargeted analysis workflow
Table S3: "Unknown common" compound list
Figure S2: Comparison of the node and sample signatures

Table S1: List of standards

Table S1-1 – Pesticide mixtures

Reference material	Compounds				
Mix Custom 1<br>100 mg/l (ACN)<br>Neochema	Methoxyfenozide	Tebufenozide	Pirimicarb	Bupimirate	Mandipropamid
	Metrafenone	Diethofencarb	Profenfos	Chlorpyriphos-methyl	Monocrotophos
	Pyraclostrobin	Ethoprophos	Thiodicarb	Cyproconazole	Oxamyl
	Pyriproxyfen	Flufenoxuron	Triazophos	Pirimicarb-desmethyl	
	Spiroxamine	Phosphamidon	Benalaxyl	Fluopyram	
Mix Custom 2<br>100 mg/l (ACN)<br>Neochema	Cyprodinil	Fludioxonil	Phosalone	Tebunfenpyrad	Fipronil-sulfone
	Etofenprox	Hexaconazole	Propargite	Triadimenol	Propiconazole
	Fenamidone	Indoxacarb	Propham	Trifloxystrobin	Tetraconazole
	Fenazaquin	Mepanipyrim	Pyridaben	Spinosad (A+D)	
	Fipronil	Myclobutanil	Tebuconazole	Carbaryl	
Mix Custom<br>100 mg/l (ACN)<br>Neochema	Acephate	Demeton-S-methyl-sulfone	Fenamiphos	Fenthion-oxon	Oxadixyl
	Aldicarb	Diflubenzuron	Fenarimol	Flubendiamide	Paclobutrazol
	Aldicarb-sulfone	(E)-Diniconazole	Fenoxycarb	Fluxapyroxad	Proquinazid
	Cyazofamid	Ethirimol	Fenpropidin	Hexythiazox	Prosulfocarb
	Cyromazine	Etoxazole	Fenpyroximate	Malaoxon	Triflumuron
Mix Custom<br>100 mg/l (ACN)<br>Neochema	Permethrin	Clofentezine	Difenoconazole	Flutolanil	Thiabendazole
	Fenpropathrin	Dimethomorph	Epoxiconazole	Flutriafol	Zoxamide
	Toclofos-methyl	Fosthiazate	Fenbuconazole	Fluquinconazole	
	Fenpropimorph	Azoxystrobin	Fluopicolide	Imazalil	
	Lufenuron	Buprofezin	Flusilazole	Pencycuron	
Mix Custom 3<br>100 mg/l (ACN)<br>Neochema	Ametocradin	Fenamiphos-sulfone	Fenthion-sulfoxide	Phosmet-oxon	Fenthion-oxon-sulfone
	Aldicarb-sulfoxide	Fenthion-oxon-sulfoxide	Fenthion-sulfone	Teflubenzuron	Phosmet
	Fenamiphos-sulfoxide				
Mix Custom 4<br>100 mg/l (ACN)<br>Neochema	Methiocarb-sulfone				
	Methiocarb-sulfoxide				
Mix Custom 5<br>100 mg/l (ACN)<br>Absolute Standard	Iprovalicarb	Metalaxyl	Pendimethalin	Quinoxyfen	Thiacloprid
	Kresoxim methyl	Metolachlor	Piperonyl butoxide	Sebuthylazine	Thiamethoxam
	Lenacil	Metolachlor-ESA	Propazine	Simazine	Triallate
	MCPA	Metribuzin	Propyzamide	Terbuthylazine	
	Mecoprop	Penconazole	Pyrimethanil	Terbutryn	
Mix Custom 6<br>100 mg/l (ACN)<br>Absolute	2,4,5-T	Atrazine	Carbendazim	Cybutryne	Fenhexamid
	2,4-D	Azinphos ethyl	Chlorfenvinphos	Cymoxanil	Flufenacet
	Acetamiprid	Bentazon	Chloridazon	Deethylatrazine	Imidacloprid

Standard	Acetochlor	Boscalid	Chlotianidin	Terbuthylazine-desethyl	
	Alachlor	Bromacil	Cyanazine	Atrazine-desisopropyl	
Mix Custom 7<br>100 mg/l (ACN)<br>Absolute<br>Standard	Methidathion	Monolinuron	Omethoate	Pirimiphos-ethyl	Temephos
	Methiocarb	Monuron	Oxydemeton methyl	Pirimiphos-methyl	Tetrachlorvinphos
	Mevinphos	Nicosulfuron	Pethoxamid	Pyridaphenthion	Thiophanate-methyl
Mix Custom 8<br>100 mg/l (ACN)<br>Absolute<br>Standard	Azinphos methyl	Diazinon	Ethion	Heptenophos	Linuron
	Chlopyrifos	Dimethoate	Fenthion	Imazamox	Malathion
	Chlortoluron	Diuron	Fluazifop-butyl	Isoproturon	Methamidophos

Table S1-2 Perfluorinated compound (PFAC) mixture

Reference material	Compounds	
Mix PFAC<br>2mg/l<br>(MeOH 1% H_2O)<br>Wellington	PFBA	PFNS
	PFBS	PFDA
	PFPeA	PFDS
	PFPeS	PFUnA
	PFHxA	PFDoA
	PFHxS	PFDoS
	PFHpA	PFTrDA
	PFHpS	PFTeDA
	PFOA	PFHxDA
	PFOS	PFODA
	PFNA	

Table S1-3 PFAS

Reference material	Compound
Powder<br>Toronto Research Chemicals	2,3,3,3-tetrafluoro-2-(heptafluoropropoxy)propanoic acid (GenX)
Solution obtained by the manufacturer	2,2-difluoro-2-[[2,2,4,5-tetrafluoro-5-(trifluoromethoxy)-1,3-dioxolan-4-yl]oxy]acetic acid (cC6O4)

Table S1-4 - Pesticides

Reference material	Compound
100 mg/l (ACN+0,1% formic acid) HCP	6-Chloro-1,3,5-triazine-2,4-diamine
100 mg/l (ACN+0,1% formic acid) HCP	2-Hydroxyatrazine
100 mg/l (ACN+0,1% formic acid) HCP	2-Hydroxyterbutilazine
100 mg/l (ACN+0,1% formic acid) HCP	Carbofuran

100 mg/l (ACN+0,1% formic acid) HCP	Carbofuran-3-hydroxy
100 mg/l (ACN+0,1% formic acid) HCP	Chlorantraniprole
100 mg/l (ACN) HCP	Chlorpyrifos-methyl-desmethyl
100 mg/l (ACN) HCP	Dimoxystrobin
100 mg/l (ACN+0,1% formic acid) HCP	Famoxadone
100 mg/l (ACN) HCP	Ipconazole
100 mg/l (ACN) HCP	Metalflumizone
100 mg/l (ACN) HCP	Metconazole
100 mg/l (ACN) HCP	Metolachlor-OA
100 mg/l (ACN+0,1% formic acid) HCP	Methomyl
100 mg/l (ACN+0,1% formic acid) HCP	Prochloraz
100 mg/l (ACN+0,1% formic acid) HCP	Propoxur

Table S1-5 – PPCP (Pharmaceutical and Personal Care Products)

Reference material	Compound
Powder Toronot Research Chemicals	Amoxicillin
Powder Dr Ehrenstorfer	Atenolol
Powder Dr Ehrenstorfer	Carbamazepine
Powder Dr Ehrenstorfer	Clotrimazole
Powder Dr Ehrenstorfer	Diazepam
Powder Dr Ehrenstorfer	Diclofenac
Powder Dr Ehrenstorfer	Fluconazole
Powder Dr Ehrenstorfer	Ketoprofen
Powder Dr Ehrenstorfer	Miconazole
Powder PharmaA2S	O-Desmethylvenlafaxine
Powder Dr Ehrenstorfer	Paracetamol
100 mg/l (MeOH) Neochema	Quassin
Powder Biosynth Carbosynth	Sulfamethoxazole
Powder Biosynth Carbosynth	Trimethoprim
1000 mg/l (MeOH) Absolute Standard	Venlafaxine
100 mg/l (MeOH) Neochema	Teucrin A

Table S2: Mass spectrometer settings

Experiment	Parameter	ESI+	ESI-
**Global**	Polarity	positive	negative
	Spray voltage	3'300	3'400
	Capillary Temperature	290	300
	Sheath gas	44	44
	Aux gas	12	12
	Probe heater temperature	280	300
	S-Lens RF Level	50	50
	dd-MS^2	Discovery	Discovery
**Full-MS**	Resolution	70'000	70'000
	# Scan ranges	1	1
	Scan range	75 to 1'000 m/z	75 to 1'000 m/z
	AGC target	1e6	1e6
	Maximum IT	240 ms	240 ms
	Microscans	1	1
	Spectrum data type	Profile	Profile
**dd-MS^2 Discovery**	Resolution	17'500	17'500
	Isolation window	2.0 m/z	3.0 m/z
	Isolation offset	—	—
	(N)CE / stepped (N)CE	nce: 20, 40, 90	nce: 20, 40, 90
	Fixed first mass	—	—
	Default charge state	1	1
	AGC target	2e5	2e5
	Maximum IT	65 ms	65 ms
	Loop count	2	2
	Minimum AGC target	8.00e3	8.00e3
	Intensity threshold	1.2e5	1.2e5
	Apex trigger	—	—
	Dynamic exclusion	1.5 s	Auto
	Charge exclusion	—	—
	Exclude isotopes	on	on
	Spectrum data type	Profile	Profile

Figure S1: Untargeted analysis workflow

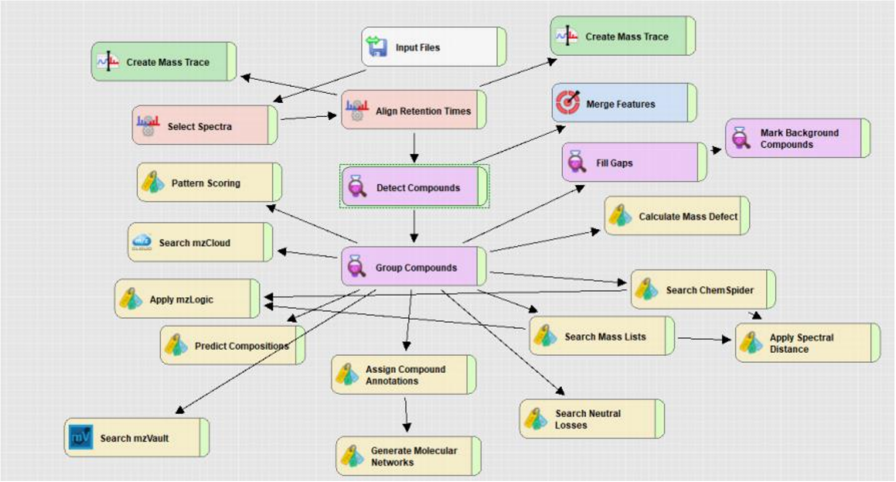

Workflow description:

"Input Files” and “Select Spectra” Node: selection of the recorded spectra for each sample and definition of the characteristics of the acquisitions such as resolution, polarity, mass fragmentation, the mass range and the retention time range to be subjected to processing.
“Align Retention Time”: Node alignment of the retention times of the sample peaks according to the protocol for non-target screening analysis [39, 40].
“Detection Compounds” Node: identification of m/z (“features”) and their related retention time, isotopic pattern, and ionic adduct.
“Mark background” Node: the features also present in the blacks are marked as background.
“Group Compounds”and “Fill gaps” Node: features grouping according to the m/z and retention time. In this step the MS2 fragmentation spectra are associated to the features and an overall chromatogram is generated filling the gaps with the data available from each sample. The result is a list of “Compounds”.
“Predict Composition” Node: the isotopic pattern accurate mass and, if present, the MS2 fragmentation are used for predicting the “Compounds” molecular formula.
The following nodes are used for compound identification in the following databases: Norman SusDat, Compound Database EFS Thermo Fisher, Chemspider), mzCloud, MassBank.
Finally for each Compound one or more structures are proposed (“annotations”) with a level of confidence.

Table S4: "Unknown common" compound list

Identification level	Name	Formula	Calc. MW	RT [min]
Level 1	Sulfamethoxazole	C10 H11 N3 O3 S	253.0521	9.189
Level 1	Terbutryn	C10 H19 N5 S	241.13609	18.001
Level 1	Diclofenac	C14 H11 Cl2 N O2	295.01685	18.602
Level 1	Trimethoprim	C14 H18 N4 O3	290.13798	8.139
Level 1	Atenolol acid	C14 H21 N O4	267.147	7.81
Level 1	Carbamazepine	C15 H12 N2 O	236.09503	14.606
Level 1	Ketoprofen	C16 H14 O3	254.09434	16.244
Level 1	O-Desmethylvenlafaxine	C16 H25 N O2	263.18852	9.625
Level 1	Diuron	C9 H10 Cl2 N2 O	232.01703	15.745
Level 1	Carbendazim	C9 H9 N3 O2	191.06968	9.323
Level 2	Adenosine	C10 H13 N5 O4	267.09661	5.58
Level 2	Hordenine	C10 H15 N O	165.11546	3.733
Level 2	1-Adamantanamine	C10 H17 N	151.13614	9.309
Level 2	Terbutryn-OH	C10 H19 N5 O S	257.13113	15.568
Level 2	Scoparone	C11 H10 O4	206.05807	11.014
Level 2	Sulfapyridine	C11 H11 N3 O2 S	249.05716	7.029
Level 2	4-Formylaminoantipyrine	C12 H13 N3 O2	231.10092	7.949
Level 2	N4-Acetylsulfamethoxazole	C12 H13 N3 O4 S	295.06275	10.953
Level 2	DEET	C12 H17 N O	191.13111	16.285
Level 2	Sotalol	C12 H20 N2 O3 S	272.11922	5.667
Level 2	Citroflex 2	C12 H20 O7	276.12087	13.895
Level 2	Memantine	C12 H21 N	179.16755	13.362
Level 2	PPG n4	C12 H26 O5	250.17778	11.459
Level 2	PPG n4	C12 H26 O5	250.17782	11.625
Level 2	PPG n4	C12 H26 O5	250.17801	11.777
Level 2	PPG n4	C12 H26 O5	250.17803	12.079
Level 2	PPG n4	C12 H26 O5	250.17791	12.287
Level 2	Fluconazole	C13 H12 F2 N6 O	306.10414	10.699
Level 2	N,N'-Diphenylguanidine	C13 H13 N3	211.1111	9.156
Level 2	4-Acetamidoantipyrine	C13 H15 N3 O2	245.11647	8.106
Level 2	Lacosamide	C13 H18 N2 O3	250.13176	10.212
Level 2	N,N'-Dicyclohexylurea	C13 H24 N2 O	224.18884	16.936
Level 2	N-Cyclohexyl-N-methylcyclohexanamine	C13 H25 N	195.1988	10.189
Level 2	N-Ethyl-4-menthane-3-carboxamide	C13 H25 N O	211.19365	17.912
Level 2	Niflumic acid	C13 H9 F3 N2 O2	282.0619	17.934
Level 2	Valsartan metabolite	C14 H10 N4 O2	266.08048	12.775
Level 2	4'-Hydroxydiclofenac	C14 H11 Cl2 N O3	311.01184	16.467
Level 2	Lidocaine	C14 H22 N2 O	234.17325	8.697
Level 2	Lidocaine N-oxide	C14 H22 N2 O2	250.16848	10.289
Level 2	Atenolol	C14 H22 N2 O3	266.16312	6.216
Level 2	Tapentadol	C14 H23 N O	221.178	10.577

Identification level	Name	Formula	Calc. MW	RT [min]
Level 2	Tetrakis(2-hydroxypropyl)ethylenediamine	C14 H32 N2 O4	292.23614	5.665
Level 2	Carbamazepine 10,11-epoxide	C15 H12 N2 O2	252.09007	12.474
Level 2	Oxcarbazepine	C15 H12 N2 O2	252.08998	13.196
Level 2	Carbamazepine 10,11-epoxide isomero	C15 H12 N2 O2	252.08985	13.301
Level 2	Clopidogrel carboxylic acid	C15 H14 Cl N O2 S	307.04344	11.405
Level 2	10-Hydroxycarbazepine	C15 H14 N2 O2	254.10565	12.444
Level 2	10-Hydroxycarbazepine isomero	C15 H14 N2 O2	254.1057	13.996
Level 2	10,11-Dihydro-10,11-dihydroxycarbamazepine	C15 H14 N2 O3	270.10057	11.962
Level 2	Nevirapine	C15 H14 N4 O	266.11728	11.996
Level 2	3,5-di-tert-Butyl-4-hydroxybenzoic acid	C15 H22 O3	250.15681	18.611
Level 2	Alprenolol	C15 H23 N O2	249.17297	9.692
Level 2	N-Desmethyltramadol	C15 H23 N O2	249.17293	10.607
Level 2	O-Desmethyl-cis-tramadol	C15 H23 N O2	249.17283	8.008
Level 2	Sulpiride	C15 H23 N3 O4 S	341.14092	5.994
Level 2	Tiapride	C15 H24 N2 O4 S	328.14564	6.704
Level 2	Metoprolol	C15 H25 N O3	267.18352	10.215
Level 2	Hexamethoxymethyl melamine	C15 H30 N6 O6	390.22262	14.895
Level 2	PPG n5	C15 H32 O6	308.21995	14.057
Level 2	Pantoprazole	C16 H15 F2 N3 O4 S	383.07536	14.541
Level 2	Sitagliptin	C16 H15 F6 N5 O	407.11845	10.533
Level 2	Clopidogrel	C16 H16 Cl N O2 S	321.05903	19.79
Level 2	Benzoylecgonine	C16 H19 N O4	289.13137	9.367
Level 2	Linezolid	C16 H20 F N3 O4	337.14374	11.399
Level 2	Tramadol	C16 H25 N O2	263.18846	10.035
Level 2	N-Desmethylvenlafaxine	C16 H25 N O2	263.18862	12.41
Level 2	Ciprofloxacin	C17 H18 F N3 O3	331.13317	9.203
Level 2	Morphine	C17 H19 N O3	285.13667	4.61
Level 2	Flecainide	C17 H20 F6 N2 O3	414.13782	13.177
Level 2	Mycophenolic acid	C17 H20 O6	320.12598	16.397
Level 2	Dextrorphan	C17 H23 N O	257.17811	9.895
Level 2	2-(3,4-Dimethoxyphenyl)-5-methylamino-2-isopropylvaleronitrile	C17 H26 N2 O2	290.19957	12.522
Level 2	Venlafaxine	C17 H27 N O2	277.20435	12.31
Level 2	Amisulpride	C17 H27 N3 O4 S	369.1722	8.198
Level 2	Clozapine	C18 H19 Cl N4	326.1296	13.76
Level 2	2-[4-(Diethylamino)-2-hydroxybenzoyl]benzoic acid	C18 H19 N O4	313.13143	17.302
Level 2	Levofloxacin	C18 H20 F N3 O4	361.14362	8.747
Level 2	Codeine	C18 H21 N O3	299.15229	6.689
Level 2	Galaxolidone	C18 H24 O2	272.17755	20.304
Level 2	Bisoprolol	C18 H31 N O4	325.22541	12.249
Level 2	PPG n6	C18 H38 O7	366.26188	15.662
Level 2	Desmethylcitalopram	C19 H19 F N2 O	310.14812	13.134
Level 2	Raltegravir	C20 H21 F N6 O5	444.15564	14.785

Identification level	Name	Formula	Calc. MW	RT [min]
Level 2	2-Ethylidene-1,5-dimethyl-3,3-diphenylpyrrolidine (EDDP)	C20 H23 N	277.18307	13.083
Level 2	Cetirizine	C21 H25 Cl N2 O3	388.15544	16.461
Level 2	Methadone	C21 H27 N O	309.20931	15.083
Level 2	Propafenone	C21 H27 N O3	341.1992	14.977
Level 2	Losartan	C22 H23 Cl N6 O	422.16226	16.027
Level 2	Eprosartan	C23 H24 N2 O4 S	424.14588	14.403
Level 2	Olmesartan	C24 H26 N6 O3	446.20682	12.548
Level 2	Valsartan	C24 H29 N5 O3	435.22744	17.163
Level 2	Ranolazine	C24 H33 N3 O4	427.2472	12.752
Level 2	Irbesartan	C25 H28 N6 O	428.23253	17.196
Level 2	Darunavir	C27 H37 N3 O7 S	547.23528	15.776
Level 2	Melamine	C3 H6 N6	126.06533	1.225
Level 2	Fexofenadine	C32 H39 N O4	501.28826	15.107
Level 2	Telmisartan	C33 H30 N4 O2	514.23752	18.529
Level 2	Clarithromycin	C38 H69 N O13	747.47719	16.543
Level 2	Tris(2-chloroethyl) phosphate	C6 H12 Cl3 O4 P	283.95406	13.895
Level 2	Benzotriazole	C6 H5 N3	119.04838	8.53
Level 2	4-Hydroxybenzotriazole	C6 H5 N3 O	135.04333	6.088
Level 2	1-Methyladenine	C6 H7 N5	149.07026	5.471
Level 2	2-Amino-6-methylmercaptopurine	C6 H7 N5 S	181.04236	7.597
Level 2	Metronidazole	C6 H9 N3 O3	171.06458	6.037
Level 2	2-Benzothiazolesulfonic acid	C7 H5 N O3 S2	214.97121	7.777
Level 2	Pregabalin	C8 H17 N O2	159.12603	6.79
Level 2	Minoxidil	C9 H15 N5 O	209.12783	9.496
Level 2	Gabapentin	C9 H17 N O2	171.12601	6.789
Level 2	Pantothenate	C9 H17 N O5	219.11065	5.525
Level 2	Pantothenate	C9 H17 N O5	219.11065	5.697
Level 2	Tris(1-chloro-2-propyl)phosphate	C9 H18 Cl3 O4 P	326.00094	17.431
Level 2	CAPSO	C9 H19 N O4 S	237.1035	4.909
Level 2	Lamotrigine	C9 H7 Cl2 N5	255.00784	10.375
Level 2	Quinoline	C9 H7 N	129.05794	10.141
Level 3	1-Phenyl-3-methyl-5-pyrazolone	C10 H10 N2 O	174.07935	6.217
Level 3	4-Phenyl-3-buten-2-one	C10 H10 O	146.07318	19.726
Level 3	4-Phenylbutyric acid	C10 H12 O2	164.0839	10.225
Level 3	4-Phenylbutyric acid	C10 H12 O2	164.0839	10.4
Level 3	NP-019992	C10 H12 O4	196.07382	8.361
Level 3	NP-019992	C10 H12 O4	196.07381	9.165
Level 3	1-(3,4-dimethoxyphenyl)ethan-1-one oxime	C10 H13 N O3	195.08974	6.725
Level 3	trans-Zeatin	C10 H13 N5 O	219.1122	2.128
Level 3	trans-Zeatin	C10 H13 N5 O	219.1122	2.426
Level 3	trans-Zeatin	C10 H13 N5 O	219.11131	2.441
Level 3	2'-Deoxyadenosine	C10 H13 N5 O3	251.10199	5.74
Level 3	2'-Deoxyguanosine	C10 H13 N5 O4	267.09659	4.784

Identification level	Name	Formula	Calc. MW	RT [min]
Level 3	6-Pentyl-2H-pyran-2-one	C10 H14 O2	166.09943	16.731
Level 3	4-(1H-imidazol-1-yl)benzoic acid	C10 H8 N2 O2	188.05887	7.869
Level 3	6-Methoxyquinoline	C10 H9 N O	159.06844	12.959
Level 3	Methyl indole-3-acetate	C11 H11 N O2	189.07933	11.607
Level 3	4-methoxy-6-(prop-2-en-1-yl)-2H-1,3-benzodioxole	C11 H12 O3	192.07893	10.596
Level 3	Sinapinic acid	C11 H12 O5	224.06839	10.739
Level 3	Indole-3-acrylic acid	C11 H9 N O2	187.0635	6.103
Level 3	3-ethyl-4-hydroxy-1-methyl-1,2-dihydroquinolin-2-one	C12 H13 N O2	203.09503	8.907
Level 3	[3-({3-[(Cyclopropylmethyl)amino]-3-oxetanyl}methyl)-1,2-oxazol-5-yl]methanol	C12 H18 N2 O3	238.13162	6.044
Level 3	6-Phenoxynicotinic acid	C12 H9 N O3	215.05847	9.139
Level 3	2,6-Di-tert-butyl-1,4-benzoquinone	C14 H20 O2	220.14662	17.619
Level 3	Isobornyl methacrylate	C14 H22 O2	222.162	16.565
Level 3	9-acridinecarboxylic acid hydrate	C14 H9 N O2	223.0634	5.536
Level 3	2-Styryl-1,3-benzoxazole	C15 H11 N O	221.084	12.071
Level 3	2-Styryl-1,3-benzoxazole	C15 H11 N O	221.0841	14.619
Level 3	1,3-diphenyl-4,5-dihydro-1H-pyrazol-5-one	C15 H12 N2 O	236.09493	13.192
Level 3	5-Hydroxy-7-(hydroxymethyl)-2-methyl-2-(5-oxotetrahydro-2-furanyl)-2,3-dihydro-4H-chromen-4-one	C15 H16 O6	292.09487	13.364
Level 3	Tebutam	C15 H23 N O	233.17842	10.477
Level 3	Alprenolol	C15 H23 N O2	249.17294	8.565
Level 3	6-(7-methyloctyl)-1H,3H,4H,6H-furo[3,4-c]furan-1-one	C15 H24 O3	252.17238	17.128
Level 3	6-(7-methyloctyl)-1H,3H,4H,6H-furo[3,4-c]furan-1-one	C15 H24 O3	252.17232	17.454
Level 3	(5E)-7-methylidene-10-oxo-4-(propan-2-yl)undec-5-enoic acid	C15 H24 O3	252.17236	17.712
Level 3	6-(7-methyloctyl)-1H,3H,4H,6H-furo[3,4-c]furan-1-one	C15 H24 O3	252.17244	18.194
Level 3	6-(7-methyloctyl)-1H,3H,4H,6H-furo[3,4-c]furan-1-one	C15 H24 O3	252.17237	18.339
Level 3	3-[4-methyl-1-(2-methylpropanoyl)-3-oxocyclohexyl]butanoic acid	C15 H24 O4	268.16739	16.241
Level 3	Tramadol	C16 H25 N O2	263.18844	9.011
Level 3	(1R,2S,3R,4R)-3-[(Cyclopentylmethyl)amino]-4-phenyl-1,2-cyclopentanediol	C17 H25 N O2	275.18898	8.745
Level 3	Galaxolidone	C18 H24 O2	272.17758	20.079
Level 3	Galaxolidone	C18 H24 O2	272.17758	20.552
Level 3	Nalbuphine	C21 H27 N O4	357.19416	13.27
Level 3	4,4'-bis(Diethylamino)benzophenone	C21 H28 N2 O	324.2203	13.087
Level 3	PPG n7	C21 H44 O8	441.33035	16.875
Level 3	PPG n8	C24 H50 O9	499.37237	17.865
Level 3	Trimethyl phosphate	C3 H9 O4 P	140.02388	6.011

Identification level	Name	Formula	Calc. MW	RT [min]
Level 3	PPG n11	C33 H68 O12	673.4979	19.973
Level 3	4-Piperidone	C5 H9 N O	99.06841	4.677
Level 3	Diglyme	C6 H14 O3	134.09434	4.961
Level 3	Diglyme	C6 H14 O3	134.09433	5.806
Level 3	Diglyme	C6 H14 O3	134.09431	6.106
Level 3	Dipropylene glycol	C6 H14 O3	134.09434	6.279
Level 3	7-Methylxanthine	C6 H6 N4 O2	166.04907	2.411
Level 3	3-Hydroxy-2-methylpyridine	C6 H7 N O	109.05287	2.089
Level 3	3-Hydroxy-2-methylpyridine	C6 H7 N O	109.05287	3.002
Level 3	7-Methylguanine	C6 H7 N5 O	165.06509	5.215
Level 3	7-Methylguanine	C6 H7 N5 O	165.06516	6.146
Level 3	1,3-Phenylenediamine	C6 H8 N2	108.06873	1.895
Level 3	2-Hydroxysimazine	C7 H13 N5 O	183.11194	2.415
Level 3	Mesalamine	C7 H7 N O3	153.04263	2.991
Level 3	Paraxanthine	C7 H8 N4 O2	180.06485	6.542
Level 3	2-Anisidine	C7 H9 N O	123.06844	5.185
Level 3	2-Anisidine	C7 H9 N O	123.06846	5.637
Level 3	3-Hydroxy-5-(hydroxymethyl)-2-methylisonicotinaldehyde oxime	C8 H10 N2 O3	182.06925	5.29
Level 3	2-Methoxy-5-methylaniline	C8 H11 N O	137.08419	12.662
Level 3	NP-019722	C8 H13 N O4	187.0845	2.152
Level 3	Tropine	C8 H15 N O	141.11544	12.83
Level 3	3,3,5,5-Tetramethylpyrroline-N-oxide	C8 H15 N O	141.11549	13.216
Level 3	NP-018661	C8 H16 O3	160.10995	8.446
Level 3	3-(2,6-Dioxocyclohexyl)propanenitrile	C9 H11 N O2	165.07894	2.58
Level 3	2-Hydroxyphenylalanine	C9 H11 N O3	181.07436	6.899
Level 3	N-Benzylethanolamine	C9 H13 N O	151.0998	6.067
Level 3	2,2,6,6-Tetramethyl-4 piperidone	C9 H17 N O	155.13109	2.895
Level 3	4-Indolecarbaldehyde	C9 H7 N O	145.05295	10.766
Level 3	3-Methyl-2-quinoxalinol	C9 H8 N2 O	160.06379	6.652
Level 3	2-methyl-1H-benzimidazole-5-carboxylic acid	C9 H8 N2 O2	176.05861	3.414
Level 3	2-methyl-1H-benzimidazole-5-carboxylic acid	C9 H8 N2 O2	176.05866	5.028
Level 3	indoline-2-carboxylic acid	C9 H9 N O2	163.06341	7.763
Level 4	MFCD00209704	[13]C3 H3 N3 O3	132.02729	0.947
Level 4	4-Chloro-1,3-(~13~C_6_)benzenediol	[13]C6 H5 Cl O2	150.01825	0.941
Level 4	Sodium bromate	Br Na O3	149.89277	1.103
Level 4		C H Cl F2 N6 O3	217.9762	0.924
Level 4		C H Cl F5 P S	205.91435	0.944
Level 4		C H Cl N6 S3	227.91166	0.959
Level 4		C H Cl2 N6 O P S4	341.82073	0.984
Level 4		C H F N O3 P3 S3	282.83218	1.128
Level 4		C H F N4 P2 S2	213.91005	0.896
Level 4		C H F O10 P2	253.90247	1.092

Identification level	Name	Formula	Calc. MW	RT [min]
Level 4		C H F S4	159.89408	1.064
Level 4		C H F12 P3 S4	461.79793	0.963
Level 4		C H F14 O2 P3 S4	531.78478	1.169
Level 4		C H F14 O7 P3 S3	579.78746	1.096
Level 4		C H F2 N2 O17 P3 S2	507.78993	1.077
Level 4		C H F2 N8 O10 P3 S2	479.84297	1.189
Level 4		C H F2 O P S4	225.86192	1.121
Level 4		C H F3 N3 P S3	238.90232	1.097
Level 4		C H F3 O3 P2 S	211.90764	1.077
Level 4		C H F4 N O10 S4	390.84183	1.034
Level 4		C H F5 N O2 P S2	248.91075	0.953
Level 4		C H F5 N O2 P S2	248.9106	1.249
Level 4		C H F5 N P3 S	246.89613	1.028
Level 4		C H F5 O14 P2 S3	489.79244	0.904
Level 4		C H F6 N S3	236.91723	1.033
Level 4		C H F6 N2 O P S3	297.88951	1.121
Level 4		C H F6 N3 O3	216.9922	0.879
Level 4		C H F6 N3 O3 P2 S3	374.85591	1.037
Level 4		C H F6 O6 P3 S3	411.80527	0.998
Level 4		C H F6 O9 P3 S3	459.78963	1.038
Level 4		C H F7 N3 O9 P3 S	456.85371	1.053
Level 4		C H F7 O13 P2 S3	511.79454	1.178
Level 4		C H F7 O4 S4	337.86432	1.069
Level 4		C H F8 N2 O6 P3 S2	445.83611	1.035
Level 4		C H F8 N4 P3 S	345.90074	1.21
Level 4		C H F9 N2 O4 P2 S2	401.87079	1.108
Level 4		C H N2 O P	87.98284	0.95
Level 4		C H N2 O5 P3	213.90991	1.052
Level 4		C H N4 O17 P3 S3	529.77084	1.035
Level 4		C H N4 P S2	163.93764	1.076
Level 4		C H2 Br Cl F5 O2 P S2	349.8028	0.942
Level 4		C H2 Br F3 N O7 P3	368.81923	1.053
Level 4		C H2 Br N3 O5 S4	342.80547	1.119
Level 4		C H2 Ca O3	101.96285	0.951
Level 4		C H2 Cl F N2 O3	143.9735	1.021
Level 4		C H2 Cl F12 N2 O P3	413.88839	1.204
Level 4		C H2 Cl F3 O3 P2 S	247.88415	0.951
Level 4		C H2 Cl F5 N5 O P S5	420.8214	1.084
Level 4		C H2 Cl N2 P S3	203.88125	0.945
Level 4		C H2 Cl2 F N P2 S3	274.81811	1.079
Level 4		C H2 Cl2 F N P2 S3	274.81835	1.162
Level 4		C H2 Cl2 F2 N6 O	221.963	0.941
Level 4		C H2 Cl2 F9 N3 O9 P2	502.84962	1.052
Level 4		C H2 Cl2 N2 O3 P2	221.89184	0.944

Identification level	Name	Formula	Calc. MW	RT [min]
Level 4		C H2 F N O3 S2	158.94574	1.047
Level 4		C H2 F N O4 P2 S4	300.83247	1.092
Level 4		C H2 F N2 O11 P3 S2	393.83003	1.152
Level 4		C H2 F N2 O2 P3 S3	281.84734	1.217
Level 4		C H2 F N2 O3 P3 S2	265.8702	1.236
Level 4		C H2 F N5 S	135.00181	0.957
Level 4		C H2 F N6 O18 P3 S2	561.80686	0.868
Level 4		C H2 F N6 P3 S2	273.89799	0.882
Level 4		C H2 F N8 O17 P3 S3	605.78968	1.035
Level 4		C H2 F O5 P S2	207.90633	1.091
Level 4		C H2 F O6 P S3	255.87369	1.058
Level 4		C H2 F2 N4 P2 S2	233.91706	0.962
Level 4		C H2 F3 N O5 P2 S	258.90814	1.05
Level 4		C H2 F3 N3 P2 S2	238.91184	0.996
Level 4		C H2 F3 O3 P3 S	243.88907	0.95
Level 4		C H2 F4 N2 O15 P2 S3	515.80333	0.868
Level 4		C H2 F5 N6 P S5	383.86051	1.091
Level 4		C H2 F5 O13 P3	409.86392	1.179
Level 4		C H2 F6 N O5 P3 S2	378.84908	1.051
Level 4		C H2 F6 N2 O3	203.99706	0.859
Level 4		C H2 F6 N7 O2 P3 S3	446.85559	1.045
Level 4		C H2 N O P S	106.95944	1.025
Level 4		C H2 N3 O2 P	118.98879	0.921
Level 4		C H2 N3 O4 P	150.97807	29.489
Level 4		C H2 N4 O4 S2	197.95197	0.927
Level 4		C H2 N4 O8 P2 S3	355.85137	1.089
Level 4		C H2 N4 O9 S2	277.92644	1.091
Level 4		C H2 N6 S4	225.92197	0.983
Level 4		C H3 Br Cl O P S	207.85144	1.122
Level 4		C H3 Cl F3 N8 O P S5	425.84149	1.092
Level 4		C H3 Cl F6 N2 S5	351.84929	0.928
Level 4		C H3 Cl2 F S2	167.90336	1.11
Level 4		C H3 Cl2 N O4 S2	226.88847	1.029
Level 4		C H3 Cl3 F N3 O2 S	244.89904	1.067
Level 4		C H3 F N4 S	122.00644	0.949
Level 4		C H3 F2 N2 O12 P S3	399.85494	1.12
Level 4		C H3 F2 N2 O13 P3 S3	477.79769	1.126
Level 4		C H3 F2 N6 O7 P S3	375.8931	1.083
Level 4		C H3 F2 O17 P S	387.87969	1.094
Level 4		C H3 F2 P3 S3	241.85747	1.045
Level 4		C H3 F3 N2 O7 P2 S4	401.82621	1.056
Level 4		C H3 F4 O14 P3 S2	471.81133	1.234
Level 4		C H3 N O3 S	108.98355	0.969
Level 4		C H3 N2 O P S	121.97004	0.965

Identification level	Name	Formula	Calc. MW	RT [min]
Level 4		C H3 N2 O12 P3	327.89008	1.059
Level 4		C H3 N2 O2 P	105.99343	0.951
Level 4		C H3 N6 P S4	257.90365	1.06
Level 4		C10 H Cl3 F O P	291.88128	12.571
Level 4		C10 H F13 N7 O13 P3 S	798.83586	3.711
Level 4		C10 H O2 P S2	247.91554	1.065
Level 4		C10 H10 Cl6 N2 O2 S4	527.77471	1.157
Level 4		C10 H10 F N3 O5	271.05956	5.829
Level 4		C10 H10 N2 O3	206.06927	3.832
Level 4		C10 H10 N2 O3	206.06925	11.732
Level 4		C10 H10 N6 O2	246.08714	12.722
Level 4		C10 H10 O3	178.06305	10.737
Level 4		C10 H10 O4	194.05818	10.893
Level 4		C10 H11 N O2	159.06857	8.383
Level 4		C10 H11 N O2	177.07892	8.625
Level 4		C10 H11 N O2	177.07915	11.463
Level 4		C10 H11 N O3	193.07412	8.072
Level 4		C10 H11 N O4	209.06908	6.606
Level 4		C10 H11 N O4	209.06898	7.709
Level 4		C10 H11 N2 O3 P	238.05126	17.104
Level 4		C10 H11 N3 O3	221.08032	6.879
Level 4		C10 H11 N5 O3	249.08605	5.368
Level 4		C10 H12 Cl N5 O2	269.06802	8.959
Level 4		C10 H12 F3 N O2 S	267.05426	11.049
Level 4		C10 H12 N2 O	176.09499	8.303
Level 4		C10 H12 N2 O2	192.08999	4.636
Level 4		C10 H12 N2 O2	192.09006	5.802
Level 4		C10 H12 N2 O2	192.08994	6.222
Level 4		C10 H12 N2 O2	192.09007	6.307
Level 4		C10 H12 N2 O2	192.09006	7.056
Level 4		C10 H12 N2 O2	192.09004	7.221
Level 4		C10 H12 N2 O3	208.0851	5.915
Level 4		C10 H12 N4 O4	252.08613	8.195
Level 4		C10 H12 O	148.08885	8.072
Level 4		C10 H12 O4	196.07112	9.795
Level 4		C10 H13 Cl N2 O	212.0698	4.41
Level 4		C10 H13 Cl3 N6 O6	417.99662	9.132
Level 4		C10 H13 F2 N5 O3	289.09831	12.197
Level 4		C10 H13 N O2	179.09474	4.363
Level 4		C10 H13 N O2	179.09479	6.076
Level 4		C10 H13 N O2	179.09479	9.505
Level 4		C10 H13 N O3	217.07374	1.907
Level 4		C10 H13 N O3	195.08976	5.716
Level 4		C10 H13 N O3	195.08976	6.87

Identification level	Name	Formula	Calc. MW	RT [min]
Level 4		C10 H13 N O3	195.08983	6.986
Level 4		C10 H13 N O3	177.0791	9.978
Level 4		C10 H13 N O3	195.08985	10.476
Level 4		C10 H13 N O3	195.08973	10.922
Level 4		C10 H13 N O3 S	227.06163	7.318
Level 4		C10 H13 N3 O3	223.09589	7.112
Level 4		C10 H13 N5 O2	235.10683	3.987
Level 4		C10 H13 N5 O3	251.10177	5.616
Level 4		C10 H13 N8 O2 P S	340.06076	11.694
Level 4		C10 H14 F2 O3 S	252.06351	11.128
Level 4		C10 H14 N2	162.11579	6.864
Level 4		C10 H14 N2 O	178.11075	4.624
Level 4		C10 H14 N2 O	178.11072	9.55
Level 4		C10 H14 N2 O2	194.10568	5.232
Level 4		C10 H14 N2 O2	194.10593	10.752
Level 4		C10 H14 N2 O2	194.10591	11.637
Level 4		C10 H14 N2 O3	210.10052	5.72
Level 4		C10 H14 O3	164.0838	11.84
Level 4		C10 H14 O3	164.08387	11.944
Level 4		C10 H15 F	154.1155	1.096
Level 4		C10 H15 F	154.11543	1.417
Level 4		C10 H15 F N3 O4 P	291.07787	8.487
Level 4		C10 H15 F2 N S	219.08961	5.221
Level 4		C10 H15 F3 N6 O2	308.12087	10.876
Level 4		C10 H15 F3 O2	224.10266	12.335
Level 4		C10 H15 N O	165.11547	6.692
Level 4		C10 H15 N O	165.11559	15.975
Level 4		C10 H15 N O2	181.11039	16.837
Level 4		C10 H15 N O2	181.11036	18.053
Level 4		C10 H15 N O3	197.10542	5.931
Level 4		C10 H15 N O4	213.10024	6.331
Level 4		C10 H15 N3 O3 S	257.08333	5.13
Level 4		C10 H15 N5 O3	253.11739	5.257
Level 4		C10 H16 F3 N7	291.14183	6.133
Level 4		C10 H16 N2 O S2	244.0711	8.423
Level 4		C10 H16 N2 O2	196.12134	3.83
Level 4		C10 H16 N2 O2	196.12133	6.311
Level 4		C10 H16 N2 O3	212.1163	6.932
Level 4		C10 H16 N2 O3 S	244.08799	1.736
Level 4		C10 H16 N2 O3 S	244.08809	4.474
Level 4		C10 H16 N2 O4	228.11132	9.537
Level 4		C10 H16 N2 O4 S	260.08364	2.067
Level 4		C10 H16 N2 O4 S	260.08295	4.965
Level 4		C10 H16 N2 P2	226.07877	1.375

Identification level	Name	Formula	Calc. MW	RT [min]
Level 4		C10 H16 N6 O2	252.13406	13.479
Level 4		C10 H16 O4	200.10522	8.047
Level 4		C10 H16 O7	248.08968	10.295
Level 4		C10 H17 F N3 P S	261.08636	9.25
Level 4		C10 H17 F3 O3	242.11296	10.85
Level 4		C10 H17 F4 N5 O	299.13679	12.562
Level 4		C10 H17 N	134.10963	9.302
Level 4		C10 H17 N O	167.13105	13.842
Level 4		C10 H17 N O2	183.12601	8.891
Level 4		C10 H17 N O4	215.11613	2.094
Level 4		C10 H17 N O8 S2	343.03941	4.721
Level 4		C10 H17 N3 O3	227.12679	1.382
Level 4		C10 H17 N3 O5	259.11657	1.862
Level 4		C10 H18 Cl3 O10 P	433.9706	9.131
Level 4		C10 H18 F N2 O3 P	264.10426	1.592
Level 4		C10 H18 F N4 P S2	308.06958	13.712
Level 4		C10 H18 F2 O S	224.10505	18.611
Level 4		C10 H18 F3 N O2 S	273.10039	8.817
Level 4		C10 H18 F3 N2 O2 P S	318.0785	11.694
Level 4		C10 H18 F3 N5 O	281.14601	3.908
Level 4		C10 H18 N2	166.14699	8.616
Level 4		C10 H18 N2 O	182.1421	11.289
Level 4		C10 H18 N2 O4	230.1268	1.04
Level 4		C10 H18 N2 O4	230.12677	1.075
Level 4		C10 H18 N2 O4	230.1268	1.119
Level 4		C10 H18 N2 O5 P2	308.06959	12.434
Level 4		C10 H18 N6 O2	254.14914	1.044
Level 4		C10 H18 N6 O3	270.14402	11.499
Level 4		C10 H18 O	154.13579	15.483
Level 4		C10 H18 O2 S	202.10289	11.016
Level 4		C10 H18 O5	218.11523	7.601
Level 4		C10 H18 O6	234.1103	7.432
Level 4		C10 H19 Br4 O4 P	549.77501	1.01
Level 4		C10 H19 F2 N3 O S	267.12178	7.942
Level 4		C10 H19 F2 O2 P S	272.08121	15.389
Level 4		C10 H19 N O	169.14662	6.607
Level 4		C10 H19 N O	169.14673	7.515
Level 4		C10 H19 N O2	185.14163	7.752
Level 4		C10 H19 N O3	201.1365	5.187
Level 4		C10 H19 N O4	217.13152	5.918
Level 4		C10 H19 N O5	233.12649	7.049
Level 4		C10 H19 N3 O2	235.13229	14.326
Level 4		C10 H19 N3 O2	235.1324	14.714
Level 4		C10 H19 N9 S	297.14787	13.199

Identification level	Name	Formula	Calc. MW	RT [min]
Level 4		C10 H19 O3 P S	250.0792	8.458
Level 4		C10 H19 O3 P S	250.07919	9.103
Level 4		C10 H2 Br Cl2 N4 O7 P3 S	563.74175	1.045
Level 4		C10 H2 F O7 P3 S	377.87196	0.954
Level 4		C10 H2 N O2 P S2	262.92645	0.967
Level 4		C10 H20 F2 N6 O S	310.13953	9.137
Level 4		C10 H20 F5 N P2 S2	375.04461	9.802
Level 4		C10 H20 N2 O4	232.14229	1.058
Level 4		C10 H20 N6 O3	272.16005	12.118
Level 4		C10 H20 O3	188.14121	14.963
Level 4		C10 H20 O4	204.13629	10.658
Level 4		C10 H20 O4	204.13631	10.829
Level 4		C10 H20 O5	220.13121	10.635
Level 4		C10 H20 O5	220.13121	11.325
Level 4		C10 H20 O6	236.12604	5.873
Level 4		C10 H21 F3 O S	246.12631	16.715
Level 4		C10 H21 F5 N2 O2 S	328.12408	14.534
Level 4		C10 H21 F5 O S	284.12334	12.567
Level 4		C10 H21 N O S2	235.10678	2.222
Level 4		C10 H21 N O2	187.15731	4.97
Level 4		C10 H21 N O2	187.1575	12.529
Level 4		C10 H21 N O4	219.14698	2.112
Level 4		C10 H21 N O4	219.147	2.475
Level 4		C10 H21 N O5	235.1419	3.299
Level 4		C10 H21 N O5	235.14194	10.029
Level 4		C10 H21 N O6	251.13697	1.904
Level 4		C10 H22 N2 O3	218.163	1.082
Level 4		C10 H22 P2 S	236.09101	5.904
Level 4		C10 H23 N O4	221.1626	8.144
Level 4		C10 H23 N O4	221.16268	8.27
Level 4		C10 H23 N O4	221.16291	10.801
Level 4		C10 H23 N O5	237.15747	8.726
Level 4		C10 H23 N O5	237.1575	9.104
Level 4		C10 H24 F2 O2 S	246.14635	11.698
Level 4		C10 H24 N2 O2	204.18378	1.271
Level 4		C10 H24 N2 O3	220.17863	1.099
Level 4		C10 H25 F2 N O S	245.16294	8.046
Level 4		C10 H25 F2 N O S	245.16265	8.318
Level 4		C10 H25 F2 N O5 S	309.1421	1.972
Level 4		C10 H25 F2 N3 O S	273.16849	1.573
Level 4		C10 H25 F4 N O2 P2	329.12983	12.691
Level 4		C10 H25 F5 O3	288.17258	19.021
Level 4		C10 H25 F6 N2 O P	334.16036	7.552
Level 4		C10 H26 Cl F2 N10 O3 P	438.15712	14.969

Identification level	Name	Formula	Calc. MW	RT [min]
Level 4		C10 H26 Cl F3 N O P	299.14006	8.999
Level 4		C10 H26 F P S3	292.09242	10.675
Level 4		C10 H26 F2 O2 S	248.16216	11.054
Level 4		C10 H26 F2 O2 S	248.16232	14.391
Level 4		C10 H26 F3 N2 P S	294.15056	1.122
Level 4		C10 H26 N2 O S3	286.12111	15.824
Level 4		C10 H27 F N10 S	338.2133	12.727
Level 4		C10 H27 F O10	326.15773	7.016
Level 4		C10 H27 F2 N O2 S	263.17321	10.211
Level 4		C10 H27 F2 N O4 S	295.16327	10.234
Level 4		C10 H27 F2 N O4 S	295.16318	11.146
Level 4		C10 H27 F2 N O4 S2	327.13543	14.46
Level 4		C10 H27 F3 N10 O7 S2	520.14625	22.135
Level 4		C10 H27 F3 N3 O2 P	309.17874	6.183
Level 4		C10 H27 F3 O3 P2	314.13811	11.963
Level 4		C10 H27 F4 N4 O3 P	358.17568	15.504
Level 4		C10 H27 O6 P	274.15437	18.88
Level 4		C10 H28 F3 O5 P	316.1633	12.439
Level 4		C10 H28 F4 O7	336.1781	13.261
Level 4		C10 H28 F8 N6 O15 S	656.12053	22.139
Level 4		C10 H28 O S2	228.15845	8.241
Level 4		C10 H29 N4 O2 P	268.20369	18.507
Level 4		C10 H29 O7 P	292.16499	16.882
Level 4		C10 H30 F N O3 P2	293.16844	17.121
Level 4		C10 H30 F3 N O S	269.19924	17.707
Level 4		C10 H30 N2 O3 P2	288.17235	17.802
Level 4		C10 H31 N3 O4 S	289.20416	19.96
Level 4		C10 H32 F6 O3 S	346.1965	13.189
Level 4		C10 H32 N9 O P	325.24657	15.093
Level 4		C10 H33 F N2 O11	376.20688	13.4
Level 4		C10 H33 F4 N O S2	323.19447	14.406
Level 4		C10 H33 F5 N O2 P3	387.16442	5.161
Level 4		C10 H34 Cl F N9 P	365.23371	19.9
Level 4		C10 H35 F N2 O2 S4	362.15584	7.364
Level 4		C10 H4 Cl2 O2	225.95821	0.944
Level 4		C10 H4 N O3 P S2	280.93696	0.963
Level 4		C10 H5 F N2 O8 P2 S	393.92256	1.048
Level 4		C10 H6 N O4 P S2	298.94734	0.992
Level 4		C10 H7 F2 N3 O2	239.05086	9.059
Level 4		C10 H7 N O2	173.04783	8.345
Level 4		C10 H8 N2 O	172.06391	8.451
Level 4		C10 H8 N2 O	172.06379	8.671
Level 4		C10 H8 O3	176.04733	8.451
Level 4		C10 H9 F3 O2	218.05557	8.317

Identification level	Name	Formula	Calc. MW	RT [min]
Level 4		C10 H9 N O	159.06858	11.274
Level 4		C10 H9 N O2	175.0635	9.525
Level 4		C10 H9 N O2	175.06364	10.594
Level 4		C10 H9 N O2	175.06357	11.606
Level 4		C11 H10 N2 O2	202.07453	4.024
Level 4		C11 H10 N2 O2	202.0743	10.578
Level 4		C11 H10 N4 O	214.08166	7.004
Level 4		C11 H10 O2	174.06808	13.598
Level 4		C11 H11 N O	173.0843	5.798
Level 4		C11 H11 N O2	189.07897	4.932
Level 4		C11 H11 N3 O2	217.08517	3.943
Level 4		C11 H11 N3 O2	217.08523	4.806
Level 4		C11 H11 N5 O2	267.07573	13.362
Level 4		C11 H12 F2 O2 S	246.05274	12.495
Level 4		C11 H12 N2 O	188.09511	7.752
Level 4		C11 H12 N2 O2	204.09014	5.904
Level 4		C11 H12 N2 O2	226.07436	8.935
Level 4		C11 H12 N2 O2	226.07425	9.895
Level 4		C11 H12 O	160.08899	6.329
Level 4		C11 H13 F N2 O3	240.09	10.225
Level 4		C11 H13 F N2 O5	272.08115	14.19
Level 4		C11 H13 F N2 O5	272.08116	14.862
Level 4		C11 H13 N O	175.09999	12.733
Level 4		C11 H13 N O2	191.09464	5.194
Level 4		C11 H13 N O2	191.09475	10.015
Level 4		C11 H13 N O3	207.0899	5.518
Level 4		C11 H13 N3 O2	219.1009	4.482
Level 4		C11 H13 N3 O3	235.09558	4.871
Level 4		C11 H14 Cl F N2	228.08229	13.898
Level 4		C11 H14 F2 O S	232.07401	12.837
Level 4		C11 H14 N2	174.11598	8.916
Level 4		C11 H14 N2 O2	206.10574	6.066
Level 4		C11 H14 N2 O2	206.10585	7.952
Level 4		C11 H15 F N3 O3 P	287.08305	10.645
Level 4		C11 H15 F3 N3 P S	309.06718	6.032
Level 4		C11 H15 F3 O3	252.09731	5.943
Level 4		C11 H15 N O2	193.1104	5.389
Level 4		C11 H15 N O2	193.1104	11.096
Level 4		C11 H15 N O2 S	225.08246	6.245
Level 4		C11 H15 N O3	209.10524	8.017
Level 4		C11 H15 N O3	209.10547	8.518
Level 4		C11 H15 N O3	209.10543	10.209
Level 4		C11 H15 N3 O4	253.10625	7.032
Level 4		C11 H15 N5 O3	265.1173	6.766

Identification level	Name	Formula	Calc. MW	RT [min]
Level 4		C11 H15 N5 O4	281.11239	5.14
Level 4		C11 H16 F2 O2 S	250.08428	9.311
Level 4		C11 H16 N2 O2	208.1213	6.936
Level 4		C11 H16 N2 O2	208.12149	7.552
Level 4		C11 H16 N4 O4	290.09766	14.36
Level 4		C11 H16 O2	180.11501	18.832
Level 4		C11 H16 O3 S	228.08219	13.027
Level 4		C11 H16 O4	212.1024	8.267
Level 4		C11 H16 O4	212.10227	8.347
Level 4		C11 H17 F N3 O3 P	289.09836	12.426
Level 4		C11 H17 N O	179.1311	5.922
Level 4		C11 H17 N O	179.13108	8.077
Level 4		C11 H17 N O5 S	275.08291	9.313
Level 4		C11 H17 N5 O3	267.13296	5.452
Level 4		C11 H18 F2 O3 S	268.09368	11.583
Level 4		C11 H18 N2 O6	274.11626	1.99
Level 4		C11 H18 N6 O3	282.144	5.691
Level 4		C11 H18 O2 S	214.10304	11.68
Level 4		C11 H19 F2 O3 P3	330.05153	13.003
Level 4		C11 H19 F2 O6 P	316.08818	17.803
Level 4		C11 H19 N O2	197.14157	11.028
Level 4		C11 H19 N O3	213.1367	8.981
Level 4		C11 H19 N O3	213.13675	12.163
Level 4		C11 H19 N O5	245.12604	1.355
Level 4		C11 H19 N O5	245.12602	1.565
Level 4		C11 H19 N3 O2	225.14793	10.389
Level 4		C11 H2 Cl F O6 P2	345.90085	1.002
Level 4		C11 H20 F N4 P S2	322.08521	14.436
Level 4		C11 H20 N2 O4	244.14226	11.634
Level 4		C11 H20 N2 O5	260.1372	2.835
Level 4		C11 H20 N2 O6	276.13187	1.815
Level 4		C11 H20 O S	200.12349	13.608
Level 4		C11 H20 O2 S	216.11904	11.202
Level 4		C11 H20 O6	248.12616	8.784
Level 4		C11 H20 O6	248.12601	8.981
Level 4		C11 H21 F2 N2 O2 P	282.13136	5.96
Level 4		C11 H21 F2 N3 O S	281.13758	5.937
Level 4		C11 H21 F3 N3 P S	315.11419	11.541
Level 4		C11 H21 F3 N3 P S	315.11409	11.77
Level 4		C11 H21 F3 N3 P S	315.11416	13.161
Level 4		C11 H21 N O	183.16246	16.914
Level 4		C11 H21 N O4	253.13154	6.829
Level 4		C11 H21 N O5	247.14225	8.079
Level 4		C11 H21 N O5	247.14205	10.534

Identification level	Name	Formula	Calc. MW	RT [min]
Level 4		C11 H21 N3	195.17368	13.02
Level 4		C11 H22 F2 N O8 P	365.10441	8.562
Level 4		C11 H22 F4 N3 O P	319.14429	1.089
Level 4		C11 H22 N6 O4	302.17036	11.498
Level 4		C11 H22 O2 S	218.13448	14.278
Level 4		C11 H22 O4	218.15174	10.88
Level 4		C11 H22 O4	218.15174	11.666
Level 4		C11 H22 O5	234.14679	9.478
Level 4		C11 H22 O5	234.14672	13.39
Level 4		C11 H22 O8	282.13155	6.059
Level 4		C11 H23 F N5 O4 P	339.14734	12.374
Level 4		C11 H23 F2 N O2 S2	303.11439	10.86
Level 4		C11 H23 F2 N O3 S2	319.10918	9.961
Level 4		C11 H23 F2 N S	239.15214	13.844
Level 4		C11 H23 F3 N3 P S	317.12988	14.289
Level 4		C11 H23 N O2	201.17296	12.458
Level 4		C11 H23 N O5	249.15747	7.602
Level 4		C11 H23 N O6	265.1525	8.786
Level 4		C11 H23 N O6	265.15255	8.982
Level 4		C11 H24 F N O6 S	317.12978	13.45
Level 4		C11 H24 F3 N3 O9	399.14656	8.314
Level 4		C11 H24 N2 O3	232.17858	6.337
Level 4		C11 H24 N9 P S	345.16107	15.792
Level 4		C11 H24 O4	220.16759	11.064
Level 4		C11 H24 O5	236.16228	10.278
Level 4		C11 H24 O5	236.16255	10.604
Level 4		C11 H24 O5	236.16255	10.723
Level 4		C11 H25 F O5	256.16765	15.149
Level 4		C11 H25 F3 N2 O6 P2	400.11437	19.273
Level 4		C11 H25 N O4	235.17837	5.545
Level 4		C11 H25 N O4	235.17819	8.274
Level 4		C11 H25 N O5	251.17307	7.89
Level 4		C11 H25 N O5	251.17313	8.452
Level 4		C11 H25 N O5	251.17313	9.106
Level 4		C11 H25 N O8	299.15816	6.066
Level 4		C11 H26 F N4 O3 P S	344.14452	11.846
Level 4		C11 H26 F6 N2 O5	380.17358	18.102
Level 4		C11 H27 F2 N O4 P2	337.13865	6.297
Level 4		C11 H27 F2 N O8 S	371.14307	6.251
Level 4		C11 H27 F3 N2 O2 S	308.17408	11.972
Level 4		C11 H27 F3 N3 O3 P	337.17487	4.693
Level 4		C11 H27 F3 S	248.17754	14.964
Level 4		C11 H27 N O4	237.19396	9.578
Level 4		C11 H27 N O4	237.19377	9.722

Identification level	Name	Formula	Calc. MW	RT [min]
Level 4		C11 H27 N O7	285.1788	6.622
Level 4		C11 H27 N7 S	289.20439	20.549
Level 4		C11 H28 F N O8	321.17899	4.598
Level 4		C11 H28 F2 O3 S	278.17316	14.02
Level 4		C11 H28 F3 N3 O4	323.20273	13.535
Level 4		C11 H29 F N2 S2	272.17543	19.742
Level 4		C11 H29 F N6 O2	296.23332	19.708
Level 4		C11 H29 F4 O5 P S	380.14212	13.388
Level 4		C11 H3 F N O14 P S2	486.8718	1.03
Level 4		C11 H3 O2 P S2	261.93124	1.061
Level 4		C11 H30 Cl2 F N O2 S2	361.10784	16.782
Level 4		C11 H30 F N O8	323.19454	11.228
Level 4		C11 H30 F3 N4 O4 P	370.19655	17.331
Level 4		C11 H30 F5 O6 P	384.17106	21.999
Level 4		C11 H31 F N2 O7	322.21099	6.426
Level 4		C11 H31 F2 N O2 S	279.20473	13.576
Level 4		C11 H31 F2 N O3 S	295.19924	14.098
Level 4		C11 H31 F2 N O6 S	343.1842	7.017
Level 4		C11 H31 F3 N3 O3 P	341.20516	10.84
Level 4		C11 H31 N O2 P2 S	303.15526	16.18
Level 4		C11 H32 F P3	276.17007	18.513
Level 4		C11 H32 N P3 S	303.14681	15.345
Level 4		C11 H32 N2 S3	288.17288	17.039
Level 4		C11 H33 F N8 S2	360.22546	20.502
Level 4		C11 H33 N P2 S	273.18086	20.549
Level 4		C11 H33 N10 P S2	400.20747	16.707
Level 4		C11 H34 F10 N O P	417.22144	8.16
Level 4		C11 H34 O6 P2	324.1837	11.392
Level 4		C11 H35 F4 N S2	321.21509	15.451
Level 4		C11 H36 F O3 P3	328.18627	16.819
Level 4		C11 H36 F3 N O5 S	351.22561	6.006
Level 4		C11 H36 F3 N O5 S	351.22569	14.843
Level 4		C11 H36 F5 N O3 S	357.2339	22.032
Level 4		C11 H37 F8 N O	351.27496	19.969
Level 4		C11 H7 Br F N O2 S	314.9364	1.054
Level 4		C11 H7 F N2 O4	250.03934	17.085
Level 4		C11 H8 N2 O2	200.05883	8.219
Level 4		C11 H9 N O3	203.05832	5.59
Level 4		C12 Br F9 O	409.89887	1.099
Level 4		C12 H Br Cl6 O2	465.73047	0.951
Level 4		C12 H10 Br2 S	343.88645	1.155
Level 4		C12 H10 N4 O2	242.07669	7.116
Level 4		C12 H10 N4 O2	220.09472	7.132
Level 4		C12 H10 N4 O2	242.07654	8.093

Identification level	Name	Formula	Calc. MW	RT [min]
Level 4		C12 H10 N4 O2	220.09488	8.192
Level 4		C12 H10 N4 O2	242.07648	8.213
Level 4		C12 H11 Cl N2 O2	250.05088	9.32
Level 4		C12 H11 F3 O2	244.07112	9.123
Level 4		C12 H11 N O	185.0843	12.297
Level 4		C12 H11 N O2	201.07911	9.146
Level 4		C12 H11 N O3	217.07402	5.098
Level 4		C12 H11 N3	197.09535	5.707
Level 4		C12 H11 N3 O	213.09017	5.315
Level 4		C12 H11 N3 O2	229.08505	6.981
Level 4		C12 H12 N2 O	200.09501	12.436
Level 4		C12 H12 N2 O	200.09527	12.91
Level 4		C12 H12 N2 O2	216.08977	7.046
Level 4		C12 H12 N4	212.10621	5.298
Level 4		C12 H12 N4	230.11679	5.3
Level 4		C12 H12 N4 O2 S	276.06811	5.491
Level 4		C12 H12 O4	220.07389	13.44
Level 4		C12 H12 O5	253.09502	9.308
Level 4		C12 H12 O5	236.06861	9.313
Level 4		C12 H13 N O2	203.0948	13.355
Level 4		C12 H13 N O3	219.08962	6.411
Level 4		C12 H13 N O4	235.08445	5.415
Level 4		C12 H13 N3 O2	231.10082	5.132
Level 4		C12 H13 N3 O2	231.10081	5.533
Level 4		C12 H14 F2 N O11 P	417.02727	1.046
Level 4		C12 H14 N2	186.11595	16.196
Level 4		C12 H14 N2 O	202.11071	7.493
Level 4		C12 H14 N2 O2	218.10579	8.454
Level 4		C12 H14 N4 O2	246.11173	11.588
Level 4		C12 H14 O2	190.0994	15.379
Level 4		C12 H14 O3	206.09195	12.077
Level 4		C12 H14 O4	222.08921	8.42
Level 4		C12 H14 O4 S3	318.00515	8.313
Level 4		C12 H15 F N4 O3	282.11217	11.245
Level 4		C12 H15 N O2	205.11035	12.873
Level 4		C12 H15 N O5	253.09507	12.53
Level 4		C12 H15 N3 O	217.12157	11.024
Level 4		C12 H15 N5 O2 S	293.09441	2.606
Level 4		C12 H15 N5 O3	277.11748	4.11
Level 4		C12 H15 N5 O3 S	309.08999	2.084
Level 4		C12 H16 F N O	209.12174	8.144
Level 4		C12 H16 F2 N2 S	258.1006	8.171
Level 4		C12 H16 F2 S	230.09442	16.742
Level 4		C12 H16 F6 O3	322.10031	11.027

Identification level	Name	Formula	Calc. MW	RT [min]
Level 4		C12 H16 N2 O2	220.12141	12.588
Level 4		C12 H16 N2 O3	236.11621	8.281
Level 4		C12 H16 N4	216.13765	8.212
Level 4		C12 H16 N4 O2	248.12732	13.384
Level 4		C12 H16 O2	192.11536	16.328
Level 4		C12 H16 O4	224.10498	10.344
Level 4		C12 H17 F N2 S2	272.08115	13.79
Level 4		C12 H17 F O5	260.10536	13.551
Level 4		C12 H17 N O2	207.12594	4.779
Level 4		C12 H17 N O2	207.12602	9.832
Level 4		C12 H17 N2 O2 P3 S	346.02236	17.178
Level 4		C12 H17 N5 O2	263.13807	3.947
Level 4		C12 H17 N5 O2	263.13807	4.29
Level 4		C12 H17 N5 O3	279.13318	7.657
Level 4		C12 H18 F6 N O P S	369.07614	14.336
Level 4		C12 H18 N2 O	206.14205	7.691
Level 4		C12 H18 N2 O	206.14202	10.431
Level 4		C12 H18 N2 O2	222.13674	5.459
Level 4		C12 H18 N2 O2	222.1368	6.327
Level 4		C12 H18 N2 O2	222.13689	13.52
Level 4		C12 H18 N2 O3	220.12117	4.1
Level 4		C12 H18 N2 O3	220.1213	12.269
Level 4		C12 H18 N2 O3 S	270.10405	5.999
Level 4		C12 H18 N2 O3 S	270.10396	7.518
Level 4		C12 H18 N6	246.15947	18.569
Level 4		C12 H18 O2	194.13084	15.549
Level 4		C12 H18 O3	210.12326	9.009
Level 4		C12 H18 O3	210.12306	12.679
Level 4		C12 H18 O4	208.11021	11.591
Level 4		C12 H18 O4	248.10486	12.603
Level 4		C12 H18 O4	208.11011	12.971
Level 4		C12 H18 O5	280.07385	14.967
Level 4		C12 H19 F3 N3 O2 P	325.11744	13.029
Level 4		C12 H19 F3 O4	284.12383	10.529
Level 4		C12 H19 N O	193.14694	8.027
Level 4		C12 H19 N O3	225.13657	8.007
Level 4		C12 H19 N O4 S	273.10361	9.616
Level 4		C12 H19 N O5 S	289.09856	10.504
Level 4		C12 H19 N O5 S	289.09848	10.953
Level 4		C12 H19 N O5 S	289.09856	11.232
Level 4		C12 H19 N O5 S	289.09861	11.324
Level 4		C12 H19 N O5 S	289.09846	12.103
Level 4		C12 H19 N O6	273.12135	6.642
Level 4		C12 H2 Br2 F6 O2	449.83275	1.083

Identification level	Name	Formula	Calc. MW	RT [min]
Level 4		C12 H2 F3 N8 P	346.00872	29.356
Level 4		C12 H20 Cl F N2 O3	294.11378	16.134
Level 4		C12 H20 F P3	276.07619	16.24
Level 4		C12 H20 F2 N O4 P	311.10919	12.389
Level 4		C12 H20 F3 N O6	331.12428	2.437
Level 4		C12 H20 N2 O3	240.14727	10.327
Level 4		C12 H20 O	180.15155	18.607
Level 4		C12 H20 O2	196.14646	16.872
Level 4		C12 H21 F2 N O S	265.13162	7.044
Level 4		C12 H21 F2 N O2 S	281.12656	9.313
Level 4		C12 H21 N	162.14097	13.35
Level 4		C12 H21 N O2	211.15719	11.506
Level 4		C12 H21 N O4	243.14719	13.188
Level 4		C12 H21 N3 O5	287.14822	5.173
Level 4		C12 H22 Cl F3 N4 O3	362.134	16.673
Level 4		C12 H22 Cl3 N O8	413.04126	9.131
Level 4		C12 H22 F2 O2 S2	300.10348	13.706
Level 4		C12 H22 F3 P S	286.11399	10.964
Level 4		C12 H22 N2 O2	226.16819	7.427
Level 4		C12 H22 N2 O6	290.14754	4.816
Level 4		C12 H22 N2 O6	290.14768	6.099
Level 4		C12 H22 N4	222.1843	1.185
Level 4		C12 H22 N4 O2	254.17403	6.702
Level 4		C12 H22 O6	284.1235	10.053
Level 4		C12 H22 O6	262.14165	10.177
Level 4		C12 H22 O6	284.12369	10.391
Level 4		C12 H22 O6	262.14144	10.531
Level 4		C12 H22 O6	262.14137	10.669
Level 4		C12 H22 O7	278.13645	10.238
Level 4		C12 H22 O7	278.13643	10.605
Level 4		C12 H22 O7	278.13636	11.029
Level 4		C12 H23 F2 N O2 S2	315.11415	13.549
Level 4		C12 H23 F2 N S	251.15199	19.079
Level 4		C12 H23 F2 O2 P S	300.11233	7.96
Level 4		C12 H23 F3 N4 O3 S	360.1436	10.794
Level 4		C12 H23 N	181.18346	8.612
Level 4		C12 H23 N O8	309.14214	2.425
Level 4		C12 H23 N3 O S2	289.12895	10.25
Level 4		C12 H24 Cl F3 N4 O P2	394.10666	19.266
Level 4		C12 H24 F2 N2 S	266.16207	11.722
Level 4		C12 H24 N2 O	212.18891	8.01
Level 4		C12 H24 N2 O	212.18875	8.727
Level 4		C12 H24 N2 O3	244.17886	6.138
Level 4		C12 H24 N2 O8 S	356.12522	12.404

Identification level	Name	Formula	Calc. MW	RT [min]
Level 4		C12 H24 N2 S2	260.13867	16.975
Level 4		C12 H24 O5	248.16213	10.676
Level 4		C12 H24 O5	248.16231	12.194
Level 4		C12 H24 O5	270.14429	14.394
Level 4		C12 H24 O6	264.15703	10.575
Level 4		C12 H24 O6	264.15703	10.881
Level 4		C12 H24 O6	264.15705	11.077
Level 4		C12 H24 O6	264.15728	11.312
Level 4		C12 H24 O6	264.1571	11.498
Level 4		C12 H24 O6	264.15737	11.592
Level 4		C12 H24 O6	264.15704	11.666
Level 4		C12 H24 O6	264.15734	11.906
Level 4		C12 H25 F N3 O3 P S2	373.10631	18.535
Level 4		C12 H25 F2 N P2 S	315.11417	12.845
Level 4		C12 H25 F2 N S	253.16778	16.702
Level 4		C12 H25 N O5	263.17327	4.683
Level 4		C12 H25 N O6	279.16799	2.471
Level 4		C12 H25 N O6	279.16784	10.163
Level 4		C12 H25 N O6	279.16789	10.469
Level 4		C12 H25 N O6	279.16826	10.671
Level 4		C12 H25 N O6	279.16829	11.052
Level 4		C12 H25 N O7	295.16333	10.595
Level 4		C12 H25 N O7	295.16299	11.337
Level 4		C12 H25 N3 O4	275.18429	1.369
Level 4		C12 H25 N5 O2	271.20069	5.99
Level 4		C12 H25 N5 O2	271.20053	6.95
Level 4		C12 H26 F3 N8 O5 P	450.17177	8.158
Level 4		C12 H26 O S2	250.14255	18.209
Level 4		C12 H26 O5	250.17781	11.158
Level 4		C12 H27 F N O P	251.18126	11.455
Level 4		C12 H27 F N O P	251.18122	12.138
Level 4		C12 H27 N O5	265.18855	11.369
Level 4		C12 H27 N O6	281.18363	11.717
Level 4		C12 H27 N O7	297.17851	4.704
Level 4		C12 H28 F N10 O P S2	442.16072	14.12
Level 4		C12 H28 F3 O3 P	308.17345	7.861
Level 4		C12 H28 F3 O3 P	308.1736	12.979
Level 4		C12 H28 F4 N O P	309.1841	14.405
Level 4		C12 H28 N2 O3	248.21	5.349
Level 4		C12 H28 S2	236.16255	10.933
Level 4		C12 H29 F2 N O S	273.19438	15.156
Level 4		C12 H29 F4 N O2 P2	357.16084	16.323
Level 4		C12 H29 N O5	267.20431	12.459
Level 4		C12 H29 N2 O P S3	344.11903	11.229

Identification level	Name	Formula	Calc. MW	RT [min]
Level 4		C12 H30 F N O8	335.19461	12.822
Level 4		C12 H30 F N O9	351.18953	13.331
Level 4		C12 H31 F2 N O3 S	307.19942	5.051
Level 4		C12 H31 F2 N O3 S	307.19946	14.406
Level 4		C12 H31 F7 O3 S2	420.16066	7.258
Level 4		C12 H31 N3 O S3	329.16273	7.083
Level 4		C12 H32 F3 N2 O4 P	356.20449	8.343
Level 4		C12 H33 F N10 P2	398.23491	13.573
Level 4		C12 H33 F4 N7 O2	383.26405	7.063
Level 4		C12 H34 F4 O7	366.22545	14.059
Level 4		C12 H34 N2 O S3	318.1836	18.29
Level 4		C12 H35 F2 N4 O4 P S	400.20719	14.566
Level 4		C12 H36 F4 S2	320.21994	16.848
Level 4		C12 H36 N10 S2	384.25592	14.035
Level 4		C12 H38 F N7 O3 S2	411.24667	7.585
Level 4		C12 H38 N5 O6 P	379.257	17.41
Level 4		C12 H39 F6 N3 O7	451.26991	21.362
Level 4		C12 H40 N P S2	293.23326	19.238
Level 4		C12 H40 N5 O3 P	333.28782	20.502
Level 4		C12 H40 N5 O6 P	381.27249	15.015
Level 4		C12 H41 F6 N O P2	391.25692	8.372
Level 4		C12 H42 N O P3 S2	373.19237	17.688
Level 4		C12 H6 Br4 O4	529.69945	1
Level 4		C12 H6 N2 O8 P2 S	399.9316	0.866
Level 4		C12 H8 F2 N2 O3 P2 S2	391.94235	1.075
Level 4		C12 H9 Cl4 N2 O2 P3	445.86346	12.568
Level 4		C12 H9 F N2 O	216.0704	13.264
Level 4		C12 H9 N5	223.08468	7.933
Level 4		C13 H12 F2 N2 O S	282.06429	12.285
Level 4		C13 H12 F6 N2 S2	374.03403	10.359
Level 4		C13 H12 N2 O	212.09511	9.341
Level 4		C13 H12 N2 O2	228.09002	7.834
Level 4		C13 H12 N2 O2	228.09013	8.968
Level 4		C13 H12 N4 O2	256.09646	14.132
Level 4		C13 H13 N O	199.09965	5.058
Level 4		C13 H13 N O	199.09986	8.343
Level 4		C13 H13 N3 O3 S	291.06789	8.377
Level 4		C13 H14 F2 N2 S	268.08501	13.947
Level 4		C13 H14 N2 O3	246.10041	10.577
Level 4		C13 H15 Cl F4 N2 O S	358.05401	14.961
Level 4		C13 H15 F S	222.08684	23.796
Level 4		C13 H15 F4 N3 S	321.09248	13.624
Level 4		C13 H15 F5 N10 O	422.13414	15.325
Level 4		C13 H15 N O3	233.10282	11.099

Identification level	Name	Formula	Calc. MW	RT [min]
Level 4		C13 H15 N3	213.12658	5.319
Level 4		C13 H16 Cl N3 O	265.09976	5.707
Level 4		C13 H16 N2 O2	232.12144	16.272
Level 4		C13 H16 N4 O S2	308.07682	8.328
Level 4		C13 H16 N4 O2	260.12718	7.465
Level 4		C13 H16 O	188.12013	17.366
Level 4		C13 H16 O3	220.11007	15.372
Level 4		C13 H17 N O	203.131	16.812
Level 4		C13 H17 N O2	219.12608	5.151
Level 4		C13 H17 N O2	219.12611	9.322
Level 4		C13 H17 N O5	267.1107	6.079
Level 4		C13 H17 N3 O2	247.1319	8.259
Level 4		C13 H17 N3 O2	247.1319	9.095
Level 4		C13 H17 N5 O	259.14226	6.317
Level 4		C13 H18 F O P	240.10801	1.306
Level 4		C13 H18 F O3 P	272.09819	10.91
Level 4		C13 H18 N O3 P	267.10199	7.23
Level 4		C13 H18 N2 O	218.14194	16.108
Level 4		C13 H18 N2 O	218.14189	16.271
Level 4		C13 H18 N2 O2	234.13704	6.012
Level 4		C13 H18 N2 O2	234.13678	13.366
Level 4		C13 H18 O	190.13595	19.141
Level 4		C13 H18 O2	206.13087	10.359
Level 4		C13 H18 O3	222.12593	17.337
Level 4		C13 H18 O5 S	286.0877	12.967
Level 4		C13 H18 O5 S	286.08753	13.704
Level 4		C13 H19 F N P	239.1239	1.328
Level 4		C13 H19 N O2	221.14157	7.295
Level 4		C13 H19 N O2	221.14159	9.878
Level 4		C13 H19 N O3	237.13651	5.538
Level 4		C13 H19 N O4	253.13148	7.935
Level 4		C13 H19 N O6 S	317.09364	8.386
Level 4		C13 H19 N3 O	233.15288	5.434
Level 4		C13 H19 N5 O	261.15888	5.914
Level 4		C13 H19 N5 O3	293.14865	8.566
Level 4		C13 H20 Cl N O	241.12348	12.095
Level 4		C13 H20 F P	226.12877	1.295
Level 4		C13 H20 F2 N4 O S	318.13273	10.188
Level 4		C13 H20 N2 O2	236.15245	6.504
Level 4		C13 H20 N6	260.17514	20.151
Level 4		C13 H20 O	192.15173	15.144
Level 4		C13 H20 O S2	256.09461	8.141
Level 4		C13 H20 O2	208.1463	16.761
Level 4		C13 H20 O3	206.13088	13.578

Identification level	Name	Formula	Calc. MW	RT [min]
Level 4		C13 H20 O4	240.13625	13.545
Level 4		C13 H20 O4	240.1361	15.916
Level 4		C13 H20 O6	254.11258	6.34
Level 4		C13 H21 F O2	228.15167	17.493
Level 4		C13 H21 F O2	228.15184	18
Level 4		C13 H21 F4 N5 O3	371.15796	12.256
Level 4		C13 H21 N	191.16737	6.862
Level 4		C13 H21 N O	207.16245	5.981
Level 4		C13 H21 N O3	239.15234	6.069
Level 4		C13 H21 N O3	239.15212	15.041
Level 4		C13 H21 N O4 S	287.11919	10.604
Level 4		C13 H21 N O4 S	287.1193	11.345
Level 4		C13 H21 N O5 S	303.11421	11.705
Level 4		C13 H21 N O5 S	303.11418	11.909
Level 4		C13 H21 N O5 S	303.11414	12.154
Level 4		C13 H21 N O5 S	303.11399	12.638
Level 4		C13 H21 N O5 S	303.11417	12.955
Level 4		C13 H21 N O5 S	303.11426	13.7
Level 4		C13 H22 F3 P S2	330.0857	9.456
Level 4		C13 H22 N2 O2	260.15265	11.761
Level 4		C13 H22 N2 O2	238.16815	13.22
Level 4		C13 H22 N6 O	278.1858	6.721
Level 4		C13 H22 O3	226.15689	16.755
Level 4		C13 H22 O3	248.13867	16.765
Level 4		C13 H22 O3	208.14646	17.902
Level 4		C13 H23 F N2 O4	290.16338	8.145
Level 4		C13 H23 F O3	246.16218	15.366
Level 4		C13 H23 F O4	262.15717	12.752
Level 4		C13 H23 F2 N S	263.15215	5.999
Level 4		C13 H23 F3 N3 O3 P	357.14216	12.64
Level 4		C13 H23 N O2	225.17274	7.457
Level 4		C13 H23 N O2	207.16247	10.764
Level 4		C13 H24 Cl N9 O	357.17843	16.67
Level 4		C13 H24 F2 N4 S2	338.14019	19.266
Level 4		C13 H24 F2 S	250.15705	16.696
Level 4		C13 H24 F2 S	250.15688	17.038
Level 4		C13 H24 F2 S	250.15682	18.862
Level 4		C13 H24 N2 O	224.1888	5.68
Level 4		C13 H24 N2 O6	304.16339	7.012
Level 4		C13 H24 N7 O2 P	341.17381	15.606
Level 4		C13 H24 O2 S2	276.12232	6.995
Level 4		C13 H25 F N2 O6	324.16893	11.174
Level 4		C13 H25 F O4	264.17269	18.463
Level 4		C13 H25 F2 N2 O3 P	326.15776	7.112

Identification level	Name	Formula	Calc. MW	RT [min]
Level 4		C13 H25 F3 O2 S	302.15208	16.668
Level 4		C13 H25 N O	211.19368	9.849
Level 4		C13 H25 N O4	259.1783	7.746
Level 4		C13 H25 N6 O P S	344.15559	12.441
Level 4		C13 H26 F5 N O2 P2	385.13713	9.636
Level 4		C13 H26 N6 O4	330.20184	14.058
Level 4		C13 H26 O14	406.13203	21.945
Level 4		C13 H26 O6	278.17308	13.965
Level 4		C13 H26 O7	294.16791	10.672
Level 4		C13 H27 F N P S	279.15874	9.129
Level 4		C13 H27 F N2 O S2	310.15454	18.639
Level 4		C13 H27 F O3	250.19354	17.597
Level 4		C13 H27 F2 N O3 S2	347.14062	9.69
Level 4		C13 H27 F3 N3 O P S	361.15633	11.474
Level 4		C13 H27 F5 O P2	356.14488	13.33
Level 4		C13 H27 N O10	357.16355	6.578
Level 4		C13 H27 N O7	309.17883	9.768
Level 4		C13 H27 N4 O P	286.1931	19.335
Level 4		C13 H27 N5 O3	301.21145	8.513
Level 4		C13 H28 F P S	266.16301	8.406
Level 4		C13 H28 F2 N3 P	295.1989	10.879
Level 4		C13 H28 O8	312.17841	7.558
Level 4		C13 H28 O8	312.17867	7.684
Level 4		C13 H29 F2 N O S	285.19404	17.08
Level 4		C13 H29 F2 N O2 S	301.18882	16.586
Level 4		C13 H29 F2 N O2 S	301.18886	20.138
Level 4		C13 H29 F2 N S	269.19904	17.126
Level 4		C13 H29 F4 O3 P	340.17848	9.537
Level 4		C13 H29 N O7	311.19454	10.687
Level 4		C13 H29 N4 O4 P	336.19387	13.444
Level 4		C13 H30 F N O3	267.22001	17.597
Level 4		C13 H30 N2 O5 S2	358.16064	13.371
Level 4		C13 H31 F2 N S	271.21446	18.833
Level 4		C13 H31 N3 O S3	341.16263	16.329
Level 4		C13 H32 F P S	270.19404	5.794
Level 4		C13 H32 O2 S4	348.12771	19.73
Level 4		C13 H33 F2 N O5 S	353.20519	13.547
Level 4		C13 H34 F2 O6 S	356.20412	8.29
Level 4		C13 H34 F6 N2 O5	412.23628	10.643
Level 4		C13 H35 F2 N O3 S	323.23046	16.924
Level 4		C13 H35 F3 N3 P	321.25165	20.306
Level 4		C13 H36 F2 P2 S	324.19811	11.426
Level 4		C13 H37 F4 N O6	379.25661	16.731
Level 4		C13 H37 F4 N O7	395.2519	6.781

Identification level	Name	Formula	Calc. MW	RT [min]
Level 4		C13 H37 F5 N O6 P	429.22674	12.873
Level 4		C13 H37 N4 O2 P	312.26643	20.515
Level 4		C13 H38 F N O11	403.24191	7.976
Level 4		C13 H38 F12 N6 O10	666.24584	17.801
Level 4		C13 H38 F4 O15	510.21514	11.454
Level 4		C13 H38 F5 N5 O3	407.28825	18.273
Level 4		C13 H39 F N9 O6 P	467.27349	15.773
Level 4		C13 H39 F2 N O S3	359.21507	7.105
Level 4		C13 H39 F2 N O S3	359.21566	7.873
Level 4		C13 H39 F2 N10 O4 P	468.28663	14.791
Level 4		C13 H39 F2 N7 O	347.31876	16.902
Level 4		C13 H39 P3	288.22727	13.001
Level 4		C13 H4 Cl F2 N5	303.01222	10.47
Level 4		C13 H40 F2 N10 O4 S	470.29286	15.849
Level 4		C13 H41 F6 N3 O2 S	417.28285	20.218
Level 4		C13 H41 N3 S3	335.24622	21.395
Level 4		C13 H41 N7 O7 S	439.27816	14.967
Level 4		C13 H43 N7 O2 S3	425.26257	15.972
Level 4		C13 H44 F5 N O7 S	453.2759	14.677
Level 4		C13 H44 N5 O P S2	381.27284	17.231
Level 4		C13 H6 Br4 Cl2 O2	579.6483	17.847
Level 4		C13 H6 F3 N5 O	305.05267	11.195
Level 4		C13 H8 N2 O2	224.0587	14.178
Level 4		C13 H8 O3	212.04764	11.112
Level 4		C13 H9 N5 O3	283.07053	11.195
Level 4		C14 H10 F16 N6	566.07115	18.612
Level 4		C14 H10 F3 N2 P	294.054	11.222
Level 4		C14 H10 N2 O2	238.0741	17.297
Level 4		C14 H12 F2 N10 S	390.09257	11.039
Level 4		C14 H12 O3	228.07869	16.712
Level 4		C14 H12 O4	244.07357	14.611
Level 4		C14 H13 N S	227.07677	1.38
Level 4		C14 H13 N3	245.09128	8.409
Level 4		C14 H13 N3 O S	271.07801	11.388
Level 4		C14 H14 Cl N O S	279.04863	12.04
Level 4		C14 H14 F4 N9 O12 P	607.04357	17.635
Level 4		C14 H14 N2 O3	258.10045	11.643
Level 4		C14 H14 N4 O	254.1167	6.704
Level 4		C14 H14 O4	246.08713	13.059
Level 4		C14 H14 O4	246.08915	13.21
Level 4		C14 H14 O4	246.08908	13.332
Level 4		C14 H14 O5	262.0841	10.218
Level 4		C14 H15 Cl N2 O2	278.08232	10.336
Level 4		C14 H15 F3 O	256.10742	11.566

Identification level	Name	Formula	Calc. MW	RT [min]
Level 4		C14 H15 N O2	229.11034	14.841
Level 4		C14 H15 N O3	245.10539	6.672
Level 4		C14 H15 N3 O	263.10156	4.325
Level 4		C14 H15 N3 O7	337.09091	8.6
Level 4		C14 H15 N7 S3	377.0544	12.402
Level 4		C14 H15 O3 P	262.07545	11.947
Level 4		C14 H16 F N4 O2 P	322.10031	10.605
Level 4		C14 H16 F7 N S2	395.06124	10.618
Level 4		C14 H16 F9 N4 O P	458.09132	19.393
Level 4		C14 H16 N2 O2	244.12152	12.391
Level 4		C14 H16 N2 O2	244.1212	15.626
Level 4		C14 H16 N2 O3	260.11633	11.024
Level 4		C14 H16 N4 O2	272.12743	6.545
Level 4		C14 H16 N4 O4	304.11352	6.074
Level 4		C14 H17 Cl F2 O3 S	338.05616	13.785
Level 4		C14 H17 N O4	263.11604	11.292
Level 4		C14 H17 N3 O2	259.13209	6.374
Level 4		C14 H17 N3 O5	307.11671	5.204
Level 4		C14 H18 F O8 P	364.07111	10.899
Level 4		C14 H18 F2 N3 O P	313.11574	9.674
Level 4		C14 H18 F4 O3	310.11831	13.539
Level 4		C14 H18 N2 O2	246.13684	6.533
Level 4		C14 H18 N2 O2	246.137	13.155
Level 4		C14 H18 N2 O3	262.13202	10.113
Level 4		C14 H18 N2 O4	278.12649	11.668
Level 4		C14 H18 N4 O4	306.13319	10.577
Level 4		C14 H18 O3	234.12574	18.828
Level 4		C14 H18 O6	282.11065	11.483
Level 4		C14 H19 N O2	233.14159	5.946
Level 4		C14 H19 N O2	233.1415	7.442
Level 4		C14 H19 N O7	313.11612	11.191
Level 4		C14 H19 N9 O S2	393.11594	17.075
Level 4		C14 H20 F2 N2 O S	302.12657	11.803
Level 4		C14 H20 O	204.15135	14.451
Level 4		C14 H20 O	204.1516	17.007
Level 4		C14 H20 O5	268.1313	13.361
Level 4		C14 H21 Cl N5 O6 P	421.09299	15.546
Level 4		C14 H21 F3 O2	278.14947	14.912
Level 4		C14 H21 F3 O2	278.14924	17.646
Level 4		C14 H21 N O	219.16246	8.173
Level 4		C14 H21 N O	219.16255	18.698
Level 4		C14 H21 N O	219.16241	19.399
Level 4		C14 H21 N O2	235.15721	8.483
Level 4		C14 H21 N O2	235.15737	20.612

Identification level	Name	Formula	Calc. MW	RT [min]
Level 4		C14 H21 N O3	251.15194	6.844
Level 4		C14 H21 N O3	251.15199	14.922
Level 4		C14 H21 N O5 S	315.11414	8.305
Level 4		C14 H21 N O5 S	315.11407	13.002
Level 4		C14 H21 N3 O	247.1684	7.249
Level 4		C14 H21 N3 O2	263.16371	6.771
Level 4		C14 H21 N9 O S2	395.13155	16.826
Level 4		C14 H22 F2 S	260.14162	15.515
Level 4		C14 H22 N2 O	234.17324	12.119
Level 4		C14 H22 O	206.16713	16.344
Level 4		C14 H22 O	206.16713	16.808
Level 4		C14 H22 O	206.16718	17.565
Level 4		C14 H22 O	206.16727	17.683
Level 4		C14 H22 O	206.16731	18.698
Level 4		C14 H22 O	206.16723	19.912
Level 4		C14 H22 O2	222.16215	13.789
Level 4		C14 H22 O2	222.16218	14.488
Level 4		C14 H22 O2	222.16209	14.71
Level 4		C14 H22 O2	222.16199	18.979
Level 4		C14 H22 O3	238.15693	15.173
Level 4		C14 H22 O3	238.15679	15.484
Level 4		C14 H22 O3	238.15679	16.033
Level 4		C14 H22 O3	238.15678	17.159
Level 4		C14 H22 O6	268.13109	11.188
Level 4		C14 H22 O6	268.13108	11.427
Level 4		C14 H22 O6	268.1311	13.113
Level 4		C14 H23 Br2 Cl4 O4 P S	615.81685	1.029
Level 4		C14 H23 F N2 O5	318.15835	13.843
Level 4		C14 H23 F O	226.17237	20.553
Level 4		C14 H23 F O9	354.13277	10.809
Level 4		C14 H23 N O	221.17784	6.813
Level 4		C14 H23 N O	221.17799	18.838
Level 4		C14 H23 N O2	237.17286	14.895
Level 4		C14 H23 N O2	237.17281	16.844
Level 4		C14 H23 N O4 S	301.13492	10.406
Level 4		C14 H23 N O5 S	317.12999	13.646
Level 4		C14 H23 N O5 S	317.12984	13.935
Level 4		C14 H23 N8 P S3	430.09535	18.609
Level 4		C14 H24 F N3 O3	301.17926	8.898
Level 4		C14 H24 F2 N O10 P	435.11024	11.007
Level 4		C14 H24 F2 O S	278.15194	19.392
Level 4		C14 H24 F2 S	262.15716	12.624
Level 4		C14 H24 F7 N3 O3	415.16907	7.086
Level 4		C14 H24 N2 O2	252.18367	8.447

Identification level	Name	Formula	Calc. MW	RT [min]
Level 4		C14 H24 O	208.18271	18.507
Level 4		C14 H24 O3	262.15318	5.28
Level 4		C14 H24 O3	240.17248	16.074
Level 4		C14 H24 O3	240.17246	16.596
Level 4		C14 H24 O3	222.16199	18.544
Level 4		C14 H24 O3	222.16203	19.237
Level 4		C14 H25 F2 N S2	309.13981	17.394
Level 4		C14 H25 F3 N3 O3 P	371.1579	10.62
Level 4		C14 H25 F4 N5	339.20466	10.676
Level 4		C14 H25 N4 O P	296.17542	20.286
Level 4		C14 H26 F N3 O5	335.18474	7.721
Level 4		C14 H26 F P S	276.14769	10.444
Level 4		C14 H26 F2 N2 O2 S	324.16894	10.984
Level 4		C14 H26 N6 O5	358.19628	14.896
Level 4		C14 H26 O4	258.18311	14.56
Level 4		C14 H26 O6	290.17312	14.405
Level 4		C14 H27 F4 N O9	429.16352	10.763
Level 4		C14 H27 N O	225.2093	18.471
Level 4		C14 H27 N O4	273.19393	11.862
Level 4		C14 H27 N O7	321.17885	4.366
Level 4		C14 H27 P S	258.15778	1.67
Level 4		C14 H28 F3 N3	295.22344	19.635
Level 4		C14 H28 N2 O4	288.20479	5.802
Level 4		C14 H28 N2 S2	288.17	16.711
Level 4		C14 H28 N4 O2	284.22116	10.574
Level 4		C14 H29 F2 N S	281.19922	14.884
Level 4		C14 H29 F2 N3 O2	309.22289	14.05
Level 4		C14 H29 F4 N2 O P	348.19473	13.765
Level 4		C14 H29 N O4 S	307.18162	8.298
Level 4		C14 H29 N O7	323.19445	6.146
Level 4		C14 H29 N O7	323.19448	11.417
Level 4		C14 H29 N O8	339.18947	11.555
Level 4		C14 H29 N5 O3	315.22697	10.024
Level 4		C14 H30 F2 N4 O2	324.23384	16.992
Level 4		C14 H30 F4 O10	434.1767	9.054
Level 4		C14 H30 F4 O3	322.21216	21.093
Level 4		C14 H30 N2 O4	290.22075	5.711
Level 4		C14 H30 N2 O4	290.22071	5.881
Level 4		C14 H30 N2 O4	290.22033	8.627
Level 4		C14 H30 O6	294.20444	12.745
Level 4		C14 H30 O9	342.18897	7.099
Level 4		C14 H32 F2 N3 P	311.23062	13.047
Level 4		C14 H32 F3 P	288.21993	17.999
Level 4		C14 H32 N2 O4	292.23615	5.911

Identification level	Name	Formula	Calc. MW	RT [min]
Level 4		C14 H32 N2 O6	324.22637	5.96
Level 4		C14 H33 F6 N2 O4 P S	470.18044	7.812
Level 4		C14 H34 F N O14	459.19523	7.827
Level 4		C14 H36 F N O9	381.23627	13.392
Level 4		C14 H36 F N O9	381.23615	15.827
Level 4		C14 H36 N2 O S3	344.19865	20.118
Level 4		C14 H37 F2 N O5 S	369.23649	13.474
Level 4		C14 H37 F3 N3 O2 P	367.25687	13.913
Level 4		C14 H38 F N O12	431.23682	8.53
Level 4		C14 H38 N O P3 S	361.18892	18.484
Level 4		C14 H38 N2 O S4	378.18661	8.335
Level 4		C14 H39 F7 O S2	420.23363	15.504
Level 4		C14 H4 N2 P2	261.98488	0.91
Level 4		C14 H40 F4 O7 P2	458.21855	1.159
Level 4		C14 H41 F O S3	340.22966	13.418
Level 4		C14 H41 F5 N3 O2 P	409.28543	20.457
Level 4		C14 H41 N7 O8 S	467.27311	7.579
Level 4		C14 H42 F N7 O3 S2	439.27828	7.464
Level 4		C14 H42 F O P3	338.24335	20.502
Level 4		C14 H43 N7 O8 S	469.28932	14.154
Level 4		C14 H45 F3 N2 O P2 S2	440.23907	14.136
Level 4		C14 H47 F10 N9 S	563.35271	17.493
Level 4		C14 H48 F3 N3 O4 S2	443.30397	13.404
Level 4		C14 H49 F2 N2 O2 P S2	410.29378	20.596
Level 4		C14 H49 F2 N7 O5 S2	497.32041	16.236
Level 4		C14 H6 F N O3	255.03277	14.552
Level 4		C14 H8 O3	224.04754	13.504
Level 4		C14 H9 N O	207.06848	13.806
Level 4		C14 H9 N O2	223.0633	13.88
Level 4		C15 H10 F8 O	358.06012	10.361
Level 4		C15 H11 N3 O	249.08619	3.861
Level 4		C15 H12 F N O2 S	289.05774	13.359
Level 4		C15 H12 F2 N4	286.10265	9.001
Level 4		C15 H12 N2 O	236.09504	12.443
Level 4		C15 H12 N6	276.1114	9.636
Level 4		C15 H12 N6	276.11246	16.97
Level 4		C15 H13 F O	228.09753	7.884
Level 4		C15 H13 F O	228.09745	8.145
Level 4		C15 H13 F O	228.09762	8.467
Level 4		C15 H13 F O	228.0976	8.726
Level 4		C15 H13 F O	228.09728	9.119
Level 4		C15 H13 N O2	239.09463	4.799
Level 4		C15 H13 N3 O3	278.1366	11.333
Level 4		C15 H14 Cl N O3 S	323.03863	12.809

Identification level	Name	Formula	Calc. MW	RT [min]
Level 4		C15 H14 N2 O	238.11071	8.654
Level 4		C15 H14 N2 O3	270.10071	10.106
Level 4		C15 H14 N4 O2	282.1106	7.979
Level 4		C15 H15 N O	225.11541	16.865
Level 4		C15 H15 N O3	257.10532	6.762
Level 4		C15 H15 N3 O4	301.1064	12.096
Level 4		C15 H16 F N7 S3	409.06178	18.114
Level 4		C15 H16 F2 N4	290.1339	6.595
Level 4		C15 H16 N2 O	240.12642	14.725
Level 4		C15 H16 N2 O2	294.07861	5.071
Level 4		C15 H16 N2 O2	256.1211	14.628
Level 4		C15 H16 N4 O3	300.11864	10.233
Level 4		C15 H16 N4 O3	300.11867	10.589
Level 4		C15 H16 N4 O3	300.11858	11.146
Level 4		C15 H16 N4 O3	278.13641	11.148
Level 4		C15 H16 N4 O3	300.11834	11.335
Level 4		C15 H16 O3	244.11005	16.596
Level 4		C15 H17 Cl N2 O2	292.09797	17.397
Level 4		C15 H17 F2 N O5 S	361.08071	12.4
Level 4		C15 H17 F3 N2 O4	346.11405	12.055
Level 4		C15 H17 F9 O10 P2	590.01531	15.336
Level 4		C15 H17 N O2	243.12622	6.889
Level 4		C15 H17 N O3 S	291.09658	5.035
Level 4		C15 H17 N3 O5	319.11679	9.767
Level 4		C15 H18 N2 O2	258.13676	10.635
Level 4		C15 H18 N2 O4	290.1267	7.287
Level 4		C15 H18 N2 O4	290.12666	7.531
Level 4		C15 H18 N3 O P	287.11932	11.532
Level 4		C15 H18 N3 O2 P	303.11382	11.325
Level 4		C15 H18 N4 O2	286.13884	11.125
Level 4		C15 H18 N4 O2	286.13926	11.505
Level 4		C15 H18 N6 O S2	362.09747	10.739
Level 4		C15 H18 O5	260.10216	6.908
Level 4		C15 H18 S	230.11289	8.263
Level 4		C15 H19 Cl N2 O2	294.11392	15.006
Level 4		C15 H19 N3 O4 S	337.10972	11.873
Level 4		C15 H19 N4 P	286.13376	17.374
Level 4		C15 H19 N5 O3	317.14876	11.769
Level 4		C15 H19 N6 O P S	362.108	18.61
Level 4		C15 H19 P S	262.09529	5.307
Level 4		C15 H20 F2 O S	286.12053	16.245
Level 4		C15 H20 F3 N3 S	331.13317	11.513
Level 4		C15 H20 N2 O2	260.15264	11.49
Level 4		C15 H20 N2 O3	276.14761	11.636

Identification level	Name	Formula	Calc. MW	RT [min]
Level 4		C15 H20 O2	232.14652	15.682
Level 4		C15 H20 O2	232.14643	18.438
Level 4		C15 H21 F3 O	274.1546	17.703
Level 4		C15 H21 F3 O2	290.14911	16.733
Level 4		C15 H21 N O2	247.15719	8.47
Level 4		C15 H21 N O3	263.15217	4.657
Level 4		C15 H21 N3 O4 S	339.12541	12.621
Level 4		C15 H22 N2 O	268.15218	6.6
Level 4		C15 H22 N2 O	268.1522	6.72
Level 4		C15 H22 N2 O S2	310.11669	5.227
Level 4		C15 H22 N2 O2	262.16815	6.158
Level 4		C15 H22 N2 O2	262.16813	6.691
Level 4		C15 H22 N2 O5	332.13484	9.591
Level 4		C15 H22 O3	272.13722	4.938
Level 4		C15 H22 O3	250.1566	11.549
Level 4		C15 H22 O3	250.15688	16.304
Level 4		C15 H22 O3	272.14138	16.319
Level 4		C15 H22 O3	268.16737	16.725
Level 4		C15 H22 O4	266.15174	17.299
Level 4		C15 H22 O6	298.13973	5.903
Level 4		C15 H23 F4 N5 O5	429.16338	10.642
Level 4		C15 H23 N O2	249.17285	7.701
Level 4		C15 H23 N O3	265.16792	6.126
Level 4		C15 H23 N O3	265.168	6.727
Level 4		C15 H23 N O5	297.1578	7.443
Level 4		C15 H23 N O5 S	329.12982	13.92
Level 4		C15 H23 N O7 S	361.11955	9.859
Level 4		C15 H24	204.18772	20.934
Level 4		C15 H24 F2 S	274.15702	18.932
Level 4		C15 H24 N4 O3	308.18355	12.14
Level 4		C15 H24 O S	252.15477	18.777
Level 4		C15 H24 O2	236.17753	17.342
Level 4		C15 H24 O2	218.16723	17.771
Level 4		C15 H24 O2	236.17749	18.775
Level 4		C15 H24 O3	270.18293	17.129
Level 4		C15 H24 O3	220.14614	18.2
Level 4		C15 H24 O4	268.16736	17.077
Level 4		C15 H24 O4	268.16737	17.722
Level 4		C15 H25 F O3	272.17758	19.061
Level 4		C15 H25 F O4	288.17308	19.349
Level 4		C15 H25 F3 N P3	369.11473	11.979
Level 4		C15 H25 F3 O2	294.18093	19.35
Level 4		C15 H25 F3 O5	342.1653	12.558
Level 4		C15 H25 N O	235.19354	8.018

Identification level	Name	Formula	Calc. MW	RT [min]
Level 4		C15 H25 N O2	251.1886	18.995
Level 4		C15 H25 N O5 S	331.14528	15.054
Level 4		C15 H25 N6 O P	336.18374	8.556
Level 4		C15 H26 N2 O3	282.1943	9.417
Level 4		C15 H26 O3	254.18807	18.832
Level 4		C15 H26 O3	236.17741	19.876
Level 4		C15 H26 O3	254.18812	19.876
Level 4		C15 H26 O4	308.13894	18.342
Level 4		C15 H26 O4	292.16498	18.358
Level 4		C15 H26 O8	334.16269	10.739
Level 4		C15 H27 F O3	274.1935	19.462
Level 4		C15 H27 F2 N O4 P2	385.13716	11.042
Level 4		C15 H27 N O S3	333.12488	8.078
Level 4		C15 H27 N O4	285.19398	16.606
Level 4		C15 H27 N O4	285.19391	16.745
Level 4		C15 H27 N O5	301.18885	17.11
Level 4		C15 H27 N3 O7	361.18477	4.07
Level 4		C15 H27 N3 O7	361.18478	5.228
Level 4		C15 H28 O7	320.1831	12.81
Level 4		C15 H28 O7	342.16519	13.015
Level 4		C15 H28 O8	336.17809	12.652
Level 4		C15 H28 O8	336.17812	12.813
Level 4		C15 H28 O8	336.1785	13.007
Level 4		C15 H28 O8	336.17813	13.378
Level 4		C15 H29 N O8	351.18937	5.09
Level 4		C15 H29 N O8	351.18937	12.723
Level 4		C15 H30 F N P2	305.18359	13.021
Level 4		C15 H30 F P3	322.15324	11.111
Level 4		C15 H30 F2 N4 O3 S	384.1995	7.267
Level 4		C15 H30 O10	370.1839	7.832
Level 4		C15 H30 O6	306.20393	13.288
Level 4		C15 H30 O6	306.20423	15.969
Level 4		C15 H30 O6	306.20422	16.695
Level 4		C15 H30 O6	306.20422	16.803
Level 4		C15 H30 O7	322.19944	13.421
Level 4		C15 H30 O7	322.19907	13.555
Level 4		C15 H31 F N2 O7	370.21173	16.363
Level 4		C15 H31 N O11	401.1899	7.429
Level 4		C15 H31 N O7	337.21004	12.797
Level 4		C15 H31 N O7	337.21019	13.018
Level 4		C15 H31 N O8	353.20515	12.646
Level 4		C15 H31 N O8	353.20507	12.816
Level 4		C15 H31 N O8	353.20464	13.011
Level 4		C15 H31 N O8	353.20454	13.281

Identification level	Name	Formula	Calc. MW	RT [min]
Level 4		C15 H32 F N O9	389.20657	11.429
Level 4		C15 H32 N2 O2 S	304.21842	8.83
Level 4		C15 H32 O9	356.20454	8.468
Level 4		C15 H33 F P2	294.20418	12.594
Level 4		C15 H33 F2 N O2 S	329.21974	7.883
Level 4		C15 H33 N O10	387.21049	7.835
Level 4		C15 H33 N O6	323.23083	13.289
Level 4		C15 H33 N O6	323.23093	16.696
Level 4		C15 H33 N O6	323.23093	16.806
Level 4		C15 H33 N O7	339.22543	13.298
Level 4		C15 H33 N O7	339.22589	13.525
Level 4		C15 H33 N O7	339.22597	13.884
Level 4		C15 H33 N O7	339.22599	14.459
Level 4		C15 H34 F N P2	309.21491	10.881
Level 4		C15 H34 F N2 O2 P	324.23431	16.897
Level 4		C15 H34 F N4 P S	352.22258	17.813
Level 4		C15 H34 F3 N2 P	330.24114	18.095
Level 4		C15 H35 N O2 S2	325.21027	12.191
Level 4		C15 H35 N O6	325.24646	14.078
Level 4		C15 H35 N O9	373.23117	8.344
Level 4		C15 H35 N O9	373.23119	8.468
Level 4		C15 H36 F N O10	409.23144	14.472
Level 4		C15 H37 F N9 O4 P S3	553.18448	8.662
Level 4		C15 H37 F2 N O3 S	349.24651	17.303
Level 4		C15 H37 F2 N O4 S	365.24123	6.54
Level 4		C15 H37 N O S2	311.23113	12.692
Level 4		C15 H38 F N O8	379.25702	17.133
Level 4		C15 H39 F2 N O4 P2	397.23137	13.571
Level 4		C15 H39 F3 N3 O4 P	413.26232	13.242
Level 4		C15 H39 F5 O7	426.26021	18.079
Level 4		C15 H40 F3 N P2 S	385.23121	10.346
Level 4		C15 H40 F3 N4 O2 P	396.28481	10.854
Level 4		C15 H40 N9 O5 P	457.28874	13.652
Level 4		C15 H43 F3 N2 O4 S	404.29042	20.59
Level 4		C15 H43 F6 N5 S	439.3141	20.943
Level 4		C15 H44 F O2 P S3	402.22341	15.329
Level 4		C15 H45 F3 N9 P3 S	533.26883	9.303
Level 4		C15 H46 N2 O12	446.30482	20.648
Level 4		C15 H46 N5 O3 P S2	439.2782	16.938
Level 4		C15 H48 N6 O S4	456.2769	15.109
Level 4		C15 H6 Cl2 F O3 P3 S	447.86131	12.565
Level 4		C15 H6 F3 N8 O10 P	545.9893	15.067
Level 4		C16 H10 Br3 Cl2 N5	578.78618	0.861
Level 4		C16 H10 N2 O2	262.07445	13.342

Identification level	Name	Formula	Calc. MW	RT [min]
Level 4		C16 H13 F2 P	274.0717	12.463
Level 4		C16 H14 N2 O3	282.10051	9.431
Level 4		C16 H14 N2 O3	282.10081	12.808
Level 4		C16 H14 N4 O S	332.07192	5.086
Level 4		C16 H14 O2	238.09946	15.396
Level 4		C16 H15 F2 N3 Si	315.10438	12.731
Level 4		C16 H15 F2 O P	292.08255	11.963
Level 4		C16 H15 F2 P	276.08754	12.447
Level 4		C16 H16 S	240.09696	10.001
Level 4		C16 H17 N O3	271.12068	16.247
Level 4		C16 H17 N O4	287.1156	4.774
Level 4		C16 H17 N O4	287.11585	5.73
Level 4		C16 H17 N O4	287.116	7.01
Level 4		C16 H17 N3 O4	337.10411	9.816
Level 4		C16 H18 N2 O2	270.1369	7.257
Level 4		C16 H18 N3 O4 P	347.10398	8.633
Level 4		C16 H18 O2	242.13101	15.656
Level 4		C16 H19 N	225.15167	9.374
Level 4		C16 H20 F2 O S	298.12047	14.031
Level 4		C16 H20 O3	260.14155	15.173
Level 4		C16 H20 O4	298.11631	2.493
Level 4		C16 H21 F P2	294.11031	14.826
Level 4		C16 H21 N O	243.16269	8.524
Level 4		C16 H21 N O	243.16247	10.218
Level 4		C16 H21 N O2	259.15724	12.914
Level 4		C16 H21 N O4	291.14719	6.636
Level 4		C16 H21 N5 O S	331.14555	14.176
Level 4		C16 H22 N2 O3	290.16363	8.274
Level 4		C16 H22 N2 O4	306.15837	6.981
Level 4		C16 H22 N2 O4	306.15831	11.269
Level 4		C16 H22 N2 O4	306.15859	12.01
Level 4		C16 H22 N2 O4	288.14758	14.767
Level 4		C16 H22 O	230.16728	17.166
Level 4		C16 H22 O2	246.1622	14.16
Level 4		C16 H22 O2	246.16213	14.472
Level 4		C16 H22 O2	246.16208	14.733
Level 4		C16 H22 O3	262.1572	13.277
Level 4		C16 H22 O3	262.15691	15.188
Level 4		C16 H22 O5	294.14702	9.693
Level 4		C16 H23 F N O6 P	375.12554	13.954
Level 4		C16 H23 F2 N O S	315.14734	6.691
Level 4		C16 H23 F2 O4 P S	380.10232	16.755
Level 4		C16 H23 F3 O	288.17018	19.051
Level 4		C16 H23 F3 O2	304.16518	14.843

Identification level	Name	Formula	Calc. MW	RT [min]
Level 4		C16 H23 N O	245.17826	6.661
Level 4		C16 H23 O P S	294.12072	18.61
Level 4		C16 H24 F N3 O2	309.18447	17.942
Level 4		C16 H24 N2 O2	276.18403	11.314
Level 4		C16 H24 N2 O2	276.18384	11.782
Level 4		C16 H24 N2 O5	324.16898	12.373
Level 4		C16 H24 O	232.18281	19.141
Level 4		C16 H24 O	232.18283	19.745
Level 4		C16 H24 O3	264.17264	15.067
Level 4		C16 H24 O3	286.15476	15.367
Level 4		C16 H24 O3	264.17245	18.613
Level 4		C16 H24 O4	302.15206	15.826
Level 4		C16 H24 O4	302.15201	16.181
Level 4		C16 H24 O4	302.15212	16.946
Level 4		C16 H24 O5	318.14718	14.547
Level 4		C16 H24 O5	318.14727	14.688
Level 4		C16 H25 N O3	279.18347	7.81
Level 4		C16 H25 N O3	279.18323	10.312
Level 4		C16 H25 N3 O4	323.18456	11.665
Level 4		C16 H26 F2 N O8 P	429.13652	12.426
Level 4		C16 H26 F2 N6 O2 P2 S	466.12872	8.971
Level 4		C16 H26 F3 N3 O2 S2	413.14096	11.718
Level 4		C16 H26 F3 O2 P	338.16298	10.3
Level 4		C16 H26 N2 O5	308.17399	11.756
Level 4		C16 H26 N2 O5	308.174	12.674
Level 4		C16 H26 O2	250.19327	16.642
Level 4		C16 H26 O2	250.19331	19.294
Level 4		C16 H26 O3	248.1776	15.694
Level 4		C16 H26 O3	248.17762	16.229
Level 4		C16 H26 O3	248.1776	16.68
Level 4		C16 H26 O3	248.17767	16.782
Level 4		C16 H26 O3	248.1776	17.044
Level 4		C16 H26 O3	248.17757	17.403
Level 4		C16 H26 O3	248.17765	17.831
Level 4		C16 H26 O3	266.18806	18.24
Level 4		C16 H26 O4	299.20996	14.217
Level 4		C16 H26 O4	264.17258	14.844
Level 4		C16 H26 O5	298.17802	17.752
Level 4		C16 H27 N O	249.2095	9.239
Level 4		C16 H27 N O2	265.20429	16.779
Level 4		C16 H27 N O2	265.20433	17.364
Level 4		C16 H28 F N O11	429.16357	12.453
Level 4		C16 H28 F N10 O4 P	474.20165	12.63
Level 4		C16 H28 O3	268.20369	18.831

Identification level	Name	Formula	Calc. MW	RT [min]
Level 4		C16 H28 O4	284.1987	17.105
Level 4		C16 H29 F2 N O S	321.19439	16.738
Level 4		C16 H29 F3 O S2	358.16054	13.545
Level 4		C16 H29 N O3	283.21465	16.979
Level 4		C16 H30 Cl F2 N P2	371.15119	12.085
Level 4		C16 H30 Cl F3 N2 O2 P2	436.14156	13.752
Level 4		C16 H30 F N P2	317.18383	16.787
Level 4		C16 H30 F2 N2 O4 P2	414.16579	13.914
Level 4		C16 H30 F2 O S	308.19892	16.76
Level 4		C16 H31 F2 N S	307.21468	19.047
Level 4		C16 H31 N S2	301.18884	18.027
Level 4		C16 H32 F2 S	294.21973	17.653
Level 4		C16 H32 O6	320.21982	14.78
Level 4		C16 H33 F S3	340.17337	7.355
Level 4		C16 H33 N O8	367.22062	12.033
Level 4		C16 H33 N O9	383.21559	12.388
Level 4		C16 H33 N7 O4 S	419.23075	20.73
Level 4		C16 H34 F2 N3 O5 P3	479.16782	7.316
Level 4		C16 H35 N	241.27691	12.096
Level 4		C16 H38 F3 N O6 P2 S	491.18394	12.626
Level 4		C16 H39 F2 N O S3	395.21589	13.626
Level 4		C16 H39 N O S2	325.24646	15.207
Level 4		C16 H4 F2 N2 S2	325.97775	5.59
Level 4		C16 H4 N8	308.05646	11.956
Level 4		C16 H40 F11 N3	483.30462	15.234
Level 4		C16 H41 F2 N O7 P2	459.23165	7.642
Level 4		C16 H41 F2 N O8 S	445.25226	8.401
Level 4		C16 H42 F N O13	475.26314	9.135
Level 4		C16 H42 F2 O P2 S	382.24004	13.385
Level 4		C16 H44 N9 O2 P S2	489.27852	9.164
Level 4		C16 H46 F3 N O7 S	453.29378	15.939
Level 4		C16 H46 N8 S	382.35598	20.104
Level 4		C16 H49 F10 N9 O S	605.36223	14.274
Level 4		C16 H49 F5 N O7 P	493.31755	21.321
Level 4		C16 H49 F6 N O10 P2	591.2744	9.499
Level 4		C16 H49 N7 O3 S3	483.30474	17.014
Level 4		C16 H50 F5 N O8	479.34601	11.391
Level 4		C16 H51 F N9 O8 P	547.35684	10.534
Level 4		C16 H51 F7 N3 O5 P	529.34538	15.862
Level 4		C16 H51 F7 N3 O7 P	561.33583	13.969
Level 4		C16 H51 F7 N3 O8 P	577.33146	10.106
Level 4		C16 H53 N7 O6 S3	535.32108	9.686
Level 4		C16 H54 Cl F6 N9 S2	585.35478	15.577
Level 4		C16 H55 F3 N10 O6 S	572.39672	18.288

Identification level	Name	Formula	Calc. MW	RT [min]
Level 4		C16 H56 N O7 P3	467.32729	21.262
Level 4		C16 H56 N P S4	421.30468	17.216
Level 4		C16 H8 Br2 S4	485.78826	1.051
Level 4		C17 H11 F O	250.07902	7.888
Level 4		C17 H11 F3	272.0811	14.542
Level 4		C17 H12 Cl N5 O2	353.06435	13.57
Level 4		C17 H13 F8 N O	399.08671	12.853
Level 4		C17 H15 F N2 S	298.09316	11.643
Level 4		C17 H15 F9	390.10356	19.389
Level 4		C17 H15 N5 O	305.12776	9.247
Level 4		C17 H16 N2 O6	344.10076	15.203
Level 4		C17 H17 N3 O S	311.10927	10.986
Level 4		C17 H17 N3 O4 S	359.09398	11.773
Level 4		C17 H18 N2 O6	346.11629	15.927
Level 4		C17 H18 N2 O6	314.0901	15.927
Level 4		C17 H19 Cl N2 S	318.09271	5.527
Level 4		C17 H19 N O	253.14304	12.612
Level 4		C17 H19 N S2	301.09855	11.63
Level 4		C17 H19 N5 O	309.15759	9.433
Level 4		C17 H20 F7 N O S	419.11514	12.325
Level 4		C17 H20 N2 O	268.15341	9.138
Level 4		C17 H20 N2 O S	300.12981	10.992
Level 4		C17 H21 N3 O3	315.15834	11.964
Level 4		C17 H22 F5 N P2 S	429.08567	10.644
Level 4		C17 H22 N4 O4 S	378.13261	19.265
Level 4		C17 H22 O	242.16696	19.235
Level 4		C17 H22 O2	258.16228	18.292
Level 4		C17 H23 N O2	273.17305	8.28
Level 4		C17 H23 N O4	305.16258	8.3
Level 4		C17 H24 F2 S	298.15672	14.07
Level 4		C17 H24 O8	356.15026	19.271
Level 4		C17 H25 N O2	275.18897	8.572
Level 4		C17 H25 N O5	323.17302	7.48
Level 4		C17 H25 N3 O4	357.16867	10.849
Level 4		C17 H26 F2 S	300.1726	13.441
Level 4		C17 H27 F4 N5 O6	473.18978	13.02
Level 4		C17 H27 N O2	277.20434	10.327
Level 4		C17 H27 N O2	277.20465	10.774
Level 4		C17 H27 N3 O3	321.2053	7.23
Level 4		C17 H27 N5 O4	365.20486	5.654
Level 4		C17 H28 N2 S2	324.16895	12.523
Level 4		C17 H28 O3	302.18867	16.023
Level 4		C17 H29 F3 O7	402.18671	13.577
Level 4		C17 H29 N O5 S	359.17656	16.779

Identification level	Name	Formula	Calc. MW	RT [min]
Level 4		C17 H29 N5 O4	367.22066	6.907
Level 4		C17 H31 N O5	329.21987	7.612
Level 4		C17 H32 F N O2 P2	363.18938	5.316
Level 4		C17 H32 O4	300.23009	21.093
Level 4		C17 H32 O4	300.23014	21.312
Level 4		C17 H33 N O4	315.24086	17.121
Level 4		C17 H33 N O8	379.2206	6.247
Level 4		C17 H33 O2 P S2	364.16564	16.752
Level 4		C17 H34 O4	324.22768	21.654
Level 4		C17 H35 F O4 P2	384.19979	7.397
Level 4		C17 H35 F3 N2 O5	404.25107	7.751
Level 4		C17 H35 N O12	445.21619	8.152
Level 4		C17 H35 N9 O4 P2	491.22852	12.628
Level 4		C17 H36 F N O3 P2	383.21559	12.512
Level 4		C17 H37 N O8	383.25204	14.02
Level 4		C17 H38 F N P2	337.24624	17.158
Level 4		C17 H38 F2 N O5 P	405.24652	7.081
Level 4		C17 H38 N2 O7	382.26905	7.738
Level 4		C17 H39 F N10 S4	530.22224	18.01
Level 4		C17 H39 F2 N O6 S	423.24679	6.945
Level 4		C17 H40 N9 O6 P	497.28404	8.152
Level 4		C17 H42 F7 N O4 P2	519.24689	16.695
Level 4		C17 H42 N9 O6 P	499.29941	15.387
Level 4		C17 H43 F2 N O4 P2	425.26252	13.798
Level 4		C17 H43 F8 N7 O2	529.33629	17.229
Level 4		C17 H43 N8 O3 P S	470.29286	16.172
Level 4		C17 H46 N9 P S2	471.30471	14.669
Level 4		C17 H48 F7 N O10 S	591.28917	12.91
Level 4		C17 H49 F6 N O7 P2	555.28969	8.473
Level 4		C17 H50 F8 N4 O8	590.3498	15.343
Level 4		C17 H51 N10 O P3	504.34606	21.346
Level 4		C17 H53 F2 N5 O10	525.37521	9.699
Level 4		C17 H6 F O4 P3 S3	481.86339	1.041
Level 4		C18 H12 F4 O2	336.07723	17.26
Level 4		C18 H13 F N6 O2	364.10749	16.909
Level 4		C18 H13 F N6 O3	380.10231	17.066
Level 4		C18 H13 N3 O6	367.0801	15.497
Level 4		C18 H15 N O2	277.11033	11.958
Level 4		C18 H15 N3 O3	321.11479	10.801
Level 4		C18 H18 F3 N O S	353.10495	12.552
Level 4		C18 H19 Cl N2 O2 S	379.10907	13.865
Level 4		C18 H19 F5	330.14001	12.534
Level 4		C18 H19 N O4	295.12089	16.611
Level 4		C18 H20 N O4 P	345.11278	12.662

Identification level	Name	Formula	Calc. MW	RT [min]
Level 4		C18 H20 N4 O S	362.11893	6.577
Level 4		C18 H20 O6	332.12604	15.493
Level 4		C18 H21 F3	294.15956	20.554
Level 4		C18 H21 F3 O	310.15444	19.203
Level 4		C18 H21 F3 O	310.15434	19.347
Level 4		C18 H21 N O2	283.15727	14.594
Level 4		C18 H21 O6 P	364.10751	17.086
Level 4		C18 H22 B	249.18146	17.436
Level 4		C18 H22 N2 O	282.17315	17.019
Level 4		C18 H22 N2 O4	330.15825	15.845
Level 4		C18 H22 N4 O4	358.16047	12.646
Level 4		C18 H22 N4 O4	358.16051	12.817
Level 4		C18 H22 N4 O4	358.16054	13.009
Level 4		C18 H22 N4 O4	358.16012	13.258
Level 4		C18 H22 O2	270.15972	17.371
Level 4		C18 H22 O3	286.15712	16.562
Level 4		C18 H22 O3	286.15677	18.283
Level 4		C18 H22 O4	302.15166	16.493
Level 4		C18 H22 O4	302.15223	17.238
Level 4		C18 H22 O5	300.13341	9.198
Level 4		C18 H23 F3	296.17554	20.161
Level 4		C18 H23 N O4	317.1629	6.505
Level 4		C18 H23 N O4	317.1628	7.014
Level 4		C18 H24 Cl N3 O2	349.15564	8.63
Level 4		C18 H24 Cl N3 O3	365.15121	9.552
Level 4		C18 H24 O	256.18275	20.288
Level 4		C18 H24 O3	288.17282	17.511
Level 4		C18 H24 O3	305.19914	18.636
Level 4		C18 H24 O3	288.17261	18.9
Level 4		C18 H24 O3	305.19916	19.213
Level 4		C18 H25 F O8 S	420.12433	7.078
Level 4		C18 H25 N O4	319.17835	15.903
Level 4		C18 H26	242.20359	21.403
Level 4		C18 H26 Cl F N O P	357.14217	10.741
Level 4		C18 H26 F2 O4	344.18096	13.697
Level 4		C18 H26 O	258.19868	16.89
Level 4		C18 H26 O3	290.18871	16.879
Level 4		C18 H26 O5	304.16762	18.293
Level 4		C18 H27 N O2	289.20397	19.004
Level 4		C18 H27 N O2	289.20422	19.095
Level 4		C18 H27 N O3	305.19906	19.344
Level 4		C18 H27 N O4	321.19407	16.838
Level 4		C18 H28 F N O6	373.18944	9.409
Level 4		C18 H28 O3	274.19343	18.388

Identification level	Name	Formula	Calc. MW	RT [min]
Level 4		C18 H28 O4	308.199	16.883
Level 4		C18 H29 N O2	291.21959	19.731
Level 4		C18 H30 N2 O2 S4	434.11889	12.452
Level 4		C18 H30 O5	343.23612	16.577
Level 4		C18 H30 O5	348.1916	16.764
Level 4		C18 H30 O5	343.23626	16.765
Level 4		C18 H30 O5	343.23626	16.883
Level 4		C18 H30 O5	348.1916	16.883
Level 4		C18 H31 F3 N3 O6 P	473.18985	11.354
Level 4		C18 H32 F3 N5 O8	503.22154	8.468
Level 4		C18 H32 N2 O4	340.23617	15.245
Level 4		C18 H32 O4	156.11505	19.653
Level 4		C18 H32 O4	334.21224	19.656
Level 4		C18 H34 F2 N2 O9	460.2223	14.355
Level 4		C18 H34 O5	312.23046	19.634
Level 4		C18 H36 O4	316.26166	20.501
Level 4		C18 H36 O8	380.241	15.208
Level 4		C18 H37 N O8	395.25189	14.54
Level 4		C18 H37 N O9	411.2467	14.551
Level 4		C18 H37 N O9	411.24639	14.909
Level 4		C18 H37 N5 O2 S3	451.21126	8.106
Level 4		C18 H38 F N O P2	365.2412	17.024
Level 4		C18 H39 N O8	397.26775	15.122
Level 4		C18 H4 F17 O9 P3 S4	907.76959	26.642
Level 4		C18 H41 N O7	383.28834	15.62
Level 4		C18 H42 F N O S3	403.24191	7.847
Level 4		C18 H42 F N O16	547.24802	9.007
Level 4		C18 H44 N10 O8	528.33409	17.212
Level 4		C18 H45 F3 N3 O8 P	519.28956	9.663
Level 4		C18 H48 F N O S4	441.25902	6.793
Level 4		C18 H48 F2 N3 O3 P3	485.28591	7.223
Level 4		C18 H56 F2 N5 O7 P S	555.36161	9.418
Level 4		C18 H56 N4 O17	600.36578	15.659
Level 4		C18 H57 F O5 S3	468.33592	21.341
Level 4		C18 H57 F5 N9 O P S2	605.37783	11.264
Level 4		C18 H60 F2 N8 O14	650.41953	14.821
Level 4		C19 H18 O3	294.12327	17.648
Level 4		C19 H19 F4 N O2 S	401.10798	8.631
Level 4		C19 H19 N3 O2 S	353.11964	14.948
Level 4		C19 H19 N3 O3	337.14272	8.998
Level 4		C19 H19 N3 O3 S	369.11471	11.738
Level 4		C19 H19 N3 O4 S	385.11061	12.591
Level 4		C19 H19 N3 O4 S	385.11016	12.788
Level 4		C19 H20 N2 O2	308.15248	8.844

Identification level	Name	Formula	Calc. MW	RT [min]
Level 4		C19 H21 Cl N2 O2 S	376.0983	5.981
Level 4		C19 H21 Cl N2 O2 S	376.09833	6.252
Level 4		C19 H21 N O	279.16231	12.002
Level 4		C19 H21 N3 O2	323.1637	11.924
Level 4		C19 H21 N5 O	335.17444	15
Level 4		C19 H22 Cl N5 O2	387.14623	13.088
Level 4		C19 H22 N2 O3 S	358.135	10.957
Level 4		C19 H22 O3	298.15716	16.367
Level 4		C19 H23 F3 O2	340.16484	15.75
Level 4		C19 H24 O S	300.15523	13.983
Level 4		C19 H25 F3 O2	342.18072	17.108
Level 4		C19 H25 N3 O	311.1997	18.458
Level 4		C19 H26 N2 O	298.20522	18.649
Level 4		C19 H26 O4	318.18313	13.163
Level 4		C19 H26 O4	318.18316	15.507
Level 4		C19 H27 F4 O2 P	394.16959	16.069
Level 4		C19 H27 N O	285.20969	10.689
Level 4		C19 H28 O7	406.14537	7.43
Level 4		C19 H29 F2 N O2 S	373.18898	8.433
Level 4		C19 H29 F2 N O4 S2	437.15116	12.595
Level 4		C19 H29 N5 O5	407.21566	6.198
Level 4		C19 H3 Br N2 O2 P2	431.8857	1.229
Level 4		C19 H30 N4 O5	384.17577	14.908
Level 4		C19 H30 N4 O5	416.20182	14.911
Level 4		C19 H31 F3 O4	380.21733	20.137
Level 4		C19 H31 N O2	305.2356	12.877
Level 4		C19 H34 O14	486.19576	17.002
Level 4		C19 H37 F O3 P2	394.21995	14.93
Level 4		C19 H37 F2 N5 O3 S	453.25757	7.138
Level 4		C19 H37 F3 N3 P3	457.21563	1.067
Level 4		C19 H38 F4 N10 O2 S2	578.25505	9.265
Level 4		C19 H39 F N2 O4 S2	442.23342	17.218
Level 4		C19 H39 F O5 P2	428.22555	8.103
Level 4		C19 H39 N O10	441.25743	14.105
Level 4		C19 H39 N3 O S3	421.22515	20.796
Level 4		C19 H4 Cl F2 N2 O4 P3 S2	553.84711	1.044
Level 4		C19 H41 F5 P2	426.26014	17.216
Level 4		C19 H41 N O3 S2	395.25188	16.687
Level 4		C19 H51 F4 N O11	545.34143	14.329
Level 4		C19 H53 F N5 O3 P3	511.33593	17.075
Level 4		C19 H55 N7 O14	605.37857	14.585
Level 4		C19 H58 F6 N2 O10 P2	650.34739	9.863
Level 4		C19 H63 F3 N10 O6 S	616.45885	19.389
Level 4		C19 H64 F5 N10 P S3	654.41465	11.338

Identification level	Name	Formula	Calc. MW	RT [min]
Level 4		C2 Cl F5 S	185.933	0.926
Level 4		C2 H F N3 O9 P S2	324.88762	1
Level 4		C2 H F N4 O2 S	163.98042	0.992
Level 4		C2 H F N4 O3 P2 S5	369.81048	0.945
Level 4		C2 H F N4 S	131.99085	0.941
Level 4		C2 H F N7 O P3	250.94376	0.955
Level 4		C2 H F N9 O P3 S	310.92146	1.034
Level 4		C2 H F O S2	123.94482	1.087
Level 4		C2 H F O11 P2	281.89752	1.044
Level 4		C2 H F O7	155.97065	1.099
Level 4		C2 H F10 P3 S4	435.80136	0.967
Level 4		C2 H F2 N O3 S4	252.87987	1.004
Level 4		C2 H F2 N2 O6 P3 S3	375.81743	1.111
Level 4		C2 H F2 N4 O9 P3 S3	451.80872	1.063
Level 4		C2 H F3 N2 P2 S2	235.90119	1.054
Level 4		C2 H F3 O3 P2 S3	287.85203	1.055
Level 4		C2 H F3 S	113.97494	0.986
Level 4		C2 H F6 O4 P3 S2	359.8426	1.148
Level 4		C2 H F7 N7 O2 P3 S3	476.84535	1.021
Level 4		C2 H N2 O2 P	115.97774	0.96
Level 4		C2 H N4 O4 P3 S3	333.83757	1.026
Level 4		C2 H N4 O6 P3	269.91112	1.126
Level 4		C2 H N4 O7 P S3	319.8745	1.032
Level 4		C2 H2 Cl F3 N O5 P S	274.90301	0.955
Level 4		C2 H2 Cl N O3 P2 S3	280.83521	1.107
Level 4		C2 H2 Cl2 O S	143.92018	1.083
Level 4		C2 H2 Cl3 F2 O4 P	263.87298	1.082
Level 4		C2 H2 F N O4 S2	186.94053	1.016
Level 4		C2 H2 F O15 P3 S3	473.77452	0.994
Level 4		C2 H2 F O4 P S2	203.91153	0.998
Level 4		C2 H2 F O4 P3 S	233.88651	1.065
Level 4		C2 H2 F3 N2 O7 P3 S2	379.84673	0.942
Level 4		C2 H2 F3 N3	125.02009	1.205
Level 4		C2 H2 F4 N O4 P3 S2	336.85675	1.057
Level 4		C2 H2 F5 N2 O15 P3 S3	577.77489	1.092
Level 4		C2 H2 F5 O3 P	199.96649	0.905
Level 4		C2 H2 F6 N4 O16 P2 S3	609.80086	1.016
Level 4		C2 H2 F7 N O S	220.97444	0.979
Level 4		C2 H2 F9 N4 O4 P S3	443.88193	1.09
Level 4		C2 H2 N O10 P3	292.88923	1.044
Level 4		C2 H2 N O5 P3 S2	276.85811	1.087
Level 4		C2 H2 N2 O17 S2	389.87947	1.049
Level 4		C2 H2 N3 O P	114.9938	0.939
Level 4		C2 H2 N3 O4 P3	224.9257	0.927

Identification level	Name	Formula	Calc. MW	RT [min]
Level 4		C2 H2 N4 O P2 S2	223.91454	1.042
Level 4		C2 H2 N6 S	142.00617	1.09
Level 4		C2 H2 O14 P2 S2	375.83666	1.017
Level 4		C2 H3 Br F4 O12	373.87438	1.021
Level 4		C2 H3 Cl2 O P3 S2	269.82156	0.976
Level 4		C2 H3 Cl3 N2 O	175.93095	1.063
Level 4		C2 H3 Cl4 F4 N P2	318.843	1.08
Level 4		C2 H3 F N2 O5 S2	217.94663	0.914
Level 4		C2 H3 F N2 O5 S2	217.947	0.992
Level 4		C2 H3 F N2 S	106.00015	0.95
Level 4		C2 H3 F O2 S2	141.95546	1.089
Level 4		C2 H3 F O8 S3	269.89745	1.081
Level 4		C2 H3 F2 N4 O15 P3 S2	517.82182	1.137
Level 4		C2 H3 F3 N O P S3	240.9063	0.989
Level 4		C2 H3 F4 N2 O8 P3 S3	447.81996	1.117
Level 4		C2 H3 F5 N O P S	214.95922	1.024
Level 4		C2 H3 F6 O10 P3 S3	489.80049	1.007
Level 4		C2 H3 F7 N2 O2	220.00845	29.362
Level 4		C2 H3 N2 O4 P S	181.95507	0.878
Level 4		C2 H3 N3 O	85.02752	1.08
Level 4		C2 H3 N6 O15 P3 S2	507.83146	1.028
Level 4		C2 H3 N8 P3 S2	295.9133	1.025
Level 4		C2 H3 O7 P3 S	263.8813	1.146
Level 4		C2 H4 Br F4 O2 P	245.90684	1.111
Level 4		C2 H4 F N5 S	149.01739	0.945
Level 4		C2 H4 F3 O14 P3 S3	497.79275	0.867
Level 4		C2 H4 F5 N O10 P2 S3	454.83928	1.096
Level 4		C2 H4 F5 O2 P S	217.95931	0.94
Level 4		C2 H4 F5 O3 P S2	265.92621	1.082
Level 4		C2 H4 F5 O4 P3 S2	343.86783	1.204
Level 4		C2 H4 F5 O7 P3 S3	423.82491	1.128
Level 4		C2 H4 N3 O2 P	133.00439	0.942
Level 4		C2 H4 N4	84.04354	1.167
Level 4		C2 H4 N4 O12 P2 S3	433.84633	1.253
Level 4		C2 H4 O4 P2 S2	217.90313	0.979
Level 4		C2 H5 Cl N2 O2	124.00409	0.94
Level 4		C2 H5 F N4 S	136.02218	0.932
Level 4		C2 H5 F2 N2 O16 P S3	477.85115	1.059
Level 4		C2 H5 F3 O8 S3	309.91051	1.1
Level 4		C2 H5 F4 O11 P S2	375.89475	1.102
Level 4		C2 H5 N2 O2 P	120.00912	0.95
Level 4		C2 H5 N2 O4 P S2	215.94314	0.848
Level 4		C2 H5 N3 O2	103.03809	1.226
Level 4		C2 H5 N4 P3 S	209.94478	0.951

Identification level	Name	Formula	Calc. MW	RT [min]
Level 4		C2 H6 Br Cl Sn	263.83647	1.09
Level 4		C2 H6 Cl2 F4 O2 S2	271.91188	1.176
Level 4		C2 H6 F2 N3 O14 P S3	460.87178	1.076
Level 4		C2 H6 F3 N O5 S	212.99193	12.034
Level 4		C2 H6 N3 O3 P	151.01494	0.939
Level 4		C2 H6 N3 P S	135.00178	1.108
Level 4		C2 H6 N4 O	102.05411	1.076
Level 4		C2 H6 P2 S	123.96637	0.95
Level 4		C2 H7 F N4 O4 S2	233.98927	0.919
Level 4		C2 H7 F2 O P3 S3	273.88369	1.063
Level 4		C2 H7 F2 O3 P3 S3	305.87367	1.1
Level 4		C2 H7 N O4 S	141.00983	1.028
Level 4		C2 H7 N O6	141.02757	0.949
Level 4		C2 H7 N2 O3 P S2	201.96364	0.916
Level 4		C2 H7 N2 O3 P3 S2	263.91113	1.044
Level 4		C2 H7 N2 O6 P3 S3	343.86803	1.115
Level 4		C20 H15 F4 N O2	377.10463	12.858
Level 4		C20 H18 S	290.11284	13.369
Level 4		C20 H20 N2 O	304.15732	13.764
Level 4		C20 H20 N2 O2	320.15246	11.921
Level 4		C20 H20 O5	340.129	12.904
Level 4		C20 H21 F N2 O	324.1639	13.051
Level 4		C20 H21 F N5 O2 P	413.14087	12.057
Level 4		C20 H21 N O2	307.15364	7.06
Level 4		C20 H22 N2 O	328.15013	14.415
Level 4		C20 H24 N2 O4	356.17335	10.052
Level 4		C20 H25 F3 O	338.18622	21.08
Level 4		C20 H28 F8 N4	476.21754	14.425
Level 4		C20 H37 O10 P	468.21278	20.82
Level 4		C20 H39 N O S2	373.24647	8.865
Level 4		C20 H40 F3 N5 O3	455.30959	16.457
Level 4		C20 H41 N O10	455.27316	15.068
Level 4		C20 H42 F N O S3	427.24171	8.623
Level 4		C20 H43 F2 O4 P3	478.23339	12.38
Level 4		C20 H43 N O2 S2	393.2727	17.572
Level 4		C20 H44 F N O P2	395.28826	17.91
Level 4		C20 H50 F3 N2 O5 P	486.34193	7.922
Level 4		C20 H51 F2 N S3	439.31461	18.098
Level 4		C20 H52 F3 N5 O15 P2 S2	785.23296	17.088
Level 4		C20 H52 N8 O15 P2	706.3026	6.14
Level 4		C20 H59 F5 N2 O13	630.39414	9.65
Level 4		C20 H60 F7 N2 O2 P3	586.37566	17.987
Level 4		C21 H16 F2 O	322.11609	19.391
Level 4		C21 H16 N2 O S	344.10073	14.845

Identification level	Name	Formula	Calc. MW	RT [min]
Level 4		C21 H17 N5 O2	371.13703	16.369
Level 4		C21 H18 O4	334.1164	5.856
Level 4		C21 H20 N6 O2	348.17351	21.565
Level 4		C21 H21 F3 N4 O S3	498.08321	18.616
Level 4		C21 H21 N3 O3 S	395.13031	16.204
Level 4		C21 H23 N3 O3 S	397.14595	14.78
Level 4		C21 H23 N3 O4 S	413.14147	15.084
Level 4		C21 H23 N3 O5 S	429.13663	12.024
Level 4		C21 H25 Cl N2 O4	404.15055	16.705
Level 4		C21 H25 F O S	344.1624	13.949
Level 4		C21 H29 F8 P	464.18703	7.649
Level 4		C21 H30 F N O9	459.19079	14.427
Level 4		C21 H30 S	314.20651	7.883
Level 4		C21 H31 F N2 O7	442.21174	15.284
Level 4		C21 H31 F N2 O7	442.21187	15.641
Level 4		C21 H32 F3 N4 O P	444.22767	15.294
Level 4		C21 H33 N O3	347.24621	9.933
Level 4		C21 H35 F N2 O8	462.23825	14.273
Level 4		C21 H37 F3 O5	426.26039	17.038
Level 4		C21 H37 F5 N O2 P	461.24816	8.833
Level 4		C21 H37 N O	319.28753	15.132
Level 4		C21 H37 N O P2	381.2361	6.469
Level 4		C21 H40 O10	452.26197	16.193
Level 4		C21 H41 Cl N O8 P3	563.17284	12.746
Level 4		C21 H41 N S3	403.24136	8.59
Level 4		C21 H42 O9	438.28281	16.522
Level 4		C21 H45 F2 N3 O3 S	457.3149	16.515
Level 4		C21 H50 F N O16	591.31124	9.666
Level 4		C22 H18 N2 S	342.1191	11.802
Level 4		C22 H22 N6 O	386.18539	14.928
Level 4		C22 H23 N7 O2	417.19026	9.668
Level 4		C22 H24 N6 O2	404.19604	13.488
Level 4		C22 H25 F2 N O4	405.17461	2.184
Level 4		C22 H25 N O2	670.38024	12.752
Level 4		C22 H27 N3 O	349.21535	13.765
Level 4		C22 H29 F9 O2 P2	558.15013	14.71
Level 4		C22 H29 N O6	403.19939	16.26
Level 4		C22 H30 F3 N3 O	409.23122	6.442
Level 4		C22 H31 F N6 O3	446.2432	16.014
Level 4		C22 H34 F2 N3 O4 P3	535.17389	11.49
Level 4		C22 H34 F3 N9 O	497.28377	7.77
Level 4		C22 H40 N3 O5 P S	489.24253	8.772
Level 4		C22 H41 N5 O5	455.31005	16.168
Level 4		C22 H43 F O P2	404.27762	17.036

Identification level	Name	Formula	Calc. MW	RT [min]
Level 4		C22 H45 N O11	499.29903	7.906
Level 4		C22 H46 F N O4 P2	469.28881	15.854
Level 4		C22 H49 F2 N5 O S3	533.30553	9.609
Level 4		C22 H55 F2 N O5 P2	513.35217	17.144
Level 4		C23 H15 N O2	337.10971	12.511
Level 4		C23 H17 N O	323.13118	16.328
Level 4		C23 H21 F O4	380.14205	12.821
Level 4		C23 H28 O8 S	464.15089	7.822
Level 4		C23 H31 N3 O4	413.23161	11.448
Level 4		C23 H31 N3 O4	413.23155	11.795
Level 4		C23 H33 F3 N O6 P	507.1987	12.956
Level 4		C23 H34 F2 N6 O8 S	592.21345	5.44
Level 4		C23 H35 N O12	517.2163	11.804
Level 4		C23 H35 N4 O4 P	462.23845	13.998
Level 4		C23 H36 N9 O5 P	549.25753	16.7
Level 4		C23 H36 O S2	392.22027	21.035
Level 4		C23 H38 F2 N2 S3	476.21782	12.495
Level 4		C23 H46 Cl F N2 O4 P2	530.26017	5.822
Level 4		C23 H48 F N O7 S	501.31495	14.034
Level 4		C23 H48 F2 N3 O8 P	563.31574	10.133
Level 4		C23 H48 F4 N2 O5	508.34876	11.263
Level 4		C23 H5 F12 O12 P3	793.88048	3.727
Level 4		C23 H51 F2 N3 O11	583.34801	14.128
Level 4		C23 H55 F2 N O7 P2	557.34145	16.454
Level 4		C23 H58 F5 N O4 P2	569.3775	17.999
Level 4		C23 H75 N9 O8 S4	733.46237	16.541
Level 4		C24 H16 Br4 Si	647.77519	1.07
Level 4		C24 H18 F5 N O2	447.12567	16.66
Level 4		C24 H19 N O	337.14587	5.58
Level 4		C24 H24 Cl F3 O2	436.14146	15.898
Level 4		C24 H26 Cl F3 O2	438.15718	13.802
Level 4		C24 H26 O2 P2	408.13968	6.688
Level 4		C24 H31 N O8	461.20507	16.197
Level 4		C24 H33 N O7	447.22591	16.691
Level 4		C24 H33 N3 O5	443.24269	13.697
Level 4		C24 H34 O5	402.24079	22.579
Level 4		C24 H35 Cl F5 N O S2	547.17729	12.176
Level 4		C24 H4 Cl O2 P3 S5	611.77271	0.868
Level 4		C24 H40 F N O5	441.28789	16.736
Level 4		C24 H41 F3 N O2 P	463.28398	14.679
Level 4		C24 H43 N4 O6 P3	576.2396	5.442
Level 4		C24 H50 F N O5 P2	513.31532	16.275
Level 4		C24 H50 F2 N3 O4 P	513.35154	17.49
Level 4		C24 H50 O13	546.3239	15.065

Identification level	Name	Formula	Calc. MW	RT [min]
Level 4		C24 H51 F N2 O5 P2	528.32568	8.157
Level 4		C24 H56 N5 O9 P	589.38311	13.513
Level 4		C24 H57 F4 N4 O2 P S	572.38833	9.417
Level 4		C24 H75 F4 N10 P S4	738.47254	13.932
Level 4		C25 H25 N7 O3	471.20198	9.245
Level 4		C25 H26 O5	384.19944	7.837
Level 4		C25 H28 N6 O3	460.22251	13.974
Level 4		C25 H3 F5 N O17 P3	776.85332	3.719
Level 4		C25 H30 N6 O3	462.23826	14.525
Level 4		C25 H32 F5 N2 O5 P	566.19769	12.173
Level 4		C25 H32 O S	380.21726	20.6
Level 4		C25 H36 F N O6	465.25176	20.879
Level 4		C25 H39 N O13	561.24224	12.172
Level 4		C25 H41 F2 N O2 S	457.28345	13.481
Level 4		C25 H52 F N O5 P2	527.33089	17.031
Level 4		C25 H53 N	367.41761	23.502
Level 4		C25 H81 N9 O3 P2 S4	745.48241	19.364
Level 4		C26 H32 O13	569.21703	15.771
Level 4		C26 H36 F2 N7 O2 P	547.26303	12.609
Level 4		C26 H36 O7	460.2465	15.673
Level 4		C26 H40 F4 N O P	489.27874	8.178
Level 4		C26 H44 Cl2 N2 O	470.28354	1.49
Level 4		C26 H51 N O17	649.31644	9.615
Level 4		C26 H55 F3 N7 P	553.41963	16.692
Level 4		C26 H56 F N O5 P2	543.36222	16.746
Level 4		C26 H63 F N8 O2 S	570.47725	29.343
Level 4		C27 H15 N O	369.11521	15.892
Level 4		C27 H19 N2 O2 P	434.11892	10.763
Level 4		C27 H29 F3 N2 O S	486.19577	18.347
Level 4		C27 H38 N7 P	491.29438	9.157
Level 4		C27 H43 N O14	605.26848	13.945
Level 4		C27 H45 F3 N2 O	470.34709	9.939
Level 4		C27 H45 N O3 P2	493.28679	14.143
Level 4		C27 H45 N O5 S	495.30237	13.727
Level 4		C27 H46 N3 O6 P S	571.28444	5.44
Level 4		C27 H55 N O12	585.37253	17.814
Level 4		C27 H56 F15 P	696.38871	11.32
Level 4		C27 H56 O10	557.41432	18.696
Level 4		C27 H57 N O11	571.39327	18.327
Level 4		C27 H57 N O6 P2 S	585.33661	9.166
Level 4		C27 H58 F N O2 P2	509.39347	16.587
Level 4		C27 H58 F N O7 P2	589.36707	14.587
Level 4		C27 H59 F N2 O11	606.41005	9.668
Level 4		C27 H62 F N O14	643.41409	18.782

Identification level	Name	Formula	Calc. MW	RT [min]
Level 4		C27 H73 F8 N5 O7	731.53964	20.465
Level 4		C27 H76 F8 N7 O4 P S	777.52903	19.757
Level 4		C28 H41 F2 N O3 S	509.27812	20.928
Level 4		C28 H47 F2 N5 O S	539.34732	21.607
Level 4		C28 H53 F2 N O3 S	521.37178	21.944
Level 4		C28 H53 N O7 P2	577.3311	10.27
Level 4		C28 H58 F N O6 P2	585.37235	18.066
Level 4		C29 H35 N2 O4 P	506.23394	12.787
Level 4		C29 H38 N10	526.32726	17.152
Level 4		C29 H40 F N O6 S	549.2575	16.607
Level 4		C29 H43 F2 N9 O5	635.33703	9.884
Level 4		C29 H47 N O15	649.29445	12.783
Level 4		C29 H59 F2 N O P2 S	569.37803	18.585
Level 4		C29 H66 F3 N O3 P2 S	627.4192	18.773
Level 4		C29 H70 F14 N8 O4	860.53068	12.093
Level 4		C3 H Br Cl F O S2	249.83258	0.989
Level 4		C3 H Br Cl3 F8 N9 O6 S	626.78917	1.029
Level 4		C3 H Cl F3 O3 P S	239.9024	0.946
Level 4		C3 H Cl3 N2 O	185.91535	1.125
Level 4		C3 H F N2 O P2 S	193.927	1.026
Level 4		C3 H F N5 O8 P3	346.90218	1.183
Level 4		C3 H F2 N4 O13 P3 S2	495.81662	1.056
Level 4		C3 H F2 P3 S2	231.87074	1.02
Level 4		C3 H F3 O13 P2 S2	427.82853	1.03
Level 4		C3 H F3 O13 P2 S2	427.82931	1.15
Level 4		C3 H F4 O12 P3 S	429.83399	1.048
Level 4		C3 H F8 N O2 P2 S3	392.85167	0.868
Level 4		C3 H F8 P3 S3	377.83265	1.089
Level 4		C3 H F8 P3 S3	377.83257	1.148
Level 4		C3 H N2 O16 P S	383.87844	1.196
Level 4		C3 H N4 O2 P3 S3	313.84734	1.117
Level 4		C3 H O12 P3 S3	417.78421	20.196
Level 4		C3 H O17 P S2	403.83965	0.928
Level 4		C3 H10 Cl2 F2 O9 P2 S4	487.80287	0.902
Level 4		C3 H10 F N2 O4 P	188.03607	0.983
Level 4		C3 H10 F2 N6 O2	200.08314	6.276
Level 4		C3 H10 O3 P2 S	187.98247	0.854
Level 4		C3 H10 O7	158.04273	1.023
Level 4		C3 H2 Br2 O4	259.83204	1.03
Level 4		C3 H2 Cl3 F3 O3 P2 S	341.82079	0.929
Level 4		C3 H2 F N2 O13 P3 S3	481.79115	1.035
Level 4		C3 H2 F O12 P3 S3	437.79079	20.826
Level 4		C3 H2 F2 N3 O P3 S3	322.85443	1.046
Level 4		C3 H2 F2 N3 O2 P3	242.93264	1.021

Identification level	Name	Formula	Calc. MW	RT [min]
Level 4		C3 H2 F3 N O3 P2 S3	314.86188	0.997
Level 4		C3 H2 F3 O4 P S	221.93641	1.087
Level 4		C3 H2 F4 O7 S3	321.8899	1.149
Level 4		C3 H2 F5 N3 O2 P2	268.95428	0.955
Level 4		C3 H2 F6 N6 O7 P2 S3	505.85253	1.087
Level 4		C3 H2 F6 N6 O8 P2 S2	489.87496	1.193
Level 4		C3 H2 F8 N3 O11 P3 S2	564.8206	1.043
Level 4		C3 H2 N P3 S	176.91175	1.092
Level 4		C3 H2 N4 O P2	171.97088	0.888
Level 4		C3 H3 Cl N2 O2 S3	229.90521	1.082
Level 4		C3 H3 Cl N4 O P2	207.94746	0.944
Level 4		C3 H3 Cl O2 P	136.95559	0.953
Level 4		C3 H3 Cl O2 P	136.95551	1.148
Level 4		C3 H3 Cl2 O5 P3 S2	345.80118	1.06
Level 4		C3 H3 F N P3 S	196.91847	1.074
Level 4		C3 H3 F N3 O3 P3 S3	336.85311	1
Level 4		C3 H3 F N4 S	146.00647	0.94
Level 4		C3 H3 F N7 O P3	264.95947	1.127
Level 4		C3 H3 F2 O3 P3	217.92643	0.992
Level 4		C3 H3 F3 N2 O P2 S5	361.82824	0.944
Level 4		C3 H3 F3 O3 S2	207.94762	1.265
Level 4		C3 H3 F4 N O3 P2 S3	334.86839	1.075
Level 4		C3 H3 O13 P3 S2	403.82311	1.206
Level 4		C3 H3 O3 P3 S2	243.87389	1.062
Level 4		C3 H3 O4 P3 S2	259.86843	1.19
Level 4		C3 H4 Br Cl N2 S	213.89609	0.958
Level 4		C3 H4 Cl F3 N7 P S5	420.85082	1.008
Level 4		C3 H4 Cl N3 O	133.00436	1.167
Level 4		C3 H4 Cl2 F2 N4 O P2	281.92094	1.129
Level 4		C3 H4 Cl2 F2 N4 O P2 S4	409.80903	0.974
Level 4		C3 H4 Cl2 F3 O3 P	245.92234	1.088
Level 4		C3 H4 F N2 O P3 S3	291.86803	1.052
Level 4		C3 H4 F N3 O P2 S4	306.86928	1.019
Level 4		C3 H4 F15 O8 P3 S	577.86003	17.543
Level 4		C3 H4 F3 N O3 S3	254.93045	0.944
Level 4		C3 H4 F3 N2 O2 P S3	283.91158	0.963
Level 4		C3 H4 F3 P3	189.9477	0.881
Level 4		C3 H4 F9 O5 P3 S	415.88558	1.175
Level 4		C3 H4 N O15 P S3	420.84828	0.973
Level 4		C3 H4 N3 O2 P S2	208.94821	0.942
Level 4		C3 H4 O3 S3	183.9325	0.941
Level 4		C3 H5 Cl2 N2 O P S2	249.89657	12.568
Level 4		C3 H5 Cl2 O P3 S5	379.75399	1.085
Level 4		C3 H5 F10 O11 P S3	533.85723	1.075

Identification level	Name	Formula	Calc. MW	RT [min]
Level 4		C3 H5 F11 O13 P2 S3	615.81843	1.14
Level 4		C3 H5 F4 P3 S2	273.89808	0.861
Level 4		C3 H5 O10 P3 S3	389.82595	1.077
Level 4		C3 H5 O15 P3 S	405.85628	0.959
Level 4		C3 H5 O4 P3 S2	261.88444	1.089
Level 4		C3 H6 F N4 O2 P3 S2	305.91249	1.088
Level 4		C3 H6 F3 N2 O3 P S5	365.86735	1.127
Level 4		C3 H6 F5 N O4 S2	278.96587	1.059
Level 4		C3 H6 F5 O3 P S2	279.94181	1.105
Level 4		C3 H6 F6 N O11 P3 S2	502.84996	1.155
Level 4		C3 H6 N3 O2 P	147.02008	0.943
Level 4		C3 H7 Br F P	171.94487	1.131
Level 4		C3 H7 Br P2 S	215.89332	0.944
Level 4		C3 H7 F N O5 P S	218.97679	0.934
Level 4		C3 H7 F N2 O S	138.02638	0.95
Level 4		C3 H7 F2 N3 O3 P2	232.99249	0.94
Level 4		C3 H7 N2 P S2	165.97896	0.941
Level 4		C3 H7 N3 O2	117.0537	1.511
Level 4		C3 H7 N3 P2 S4	274.89995	1.083
Level 4		C3 H7 N5 O2	145.0599	1.312
Level 4		C3 H7 N7 O4	205.05586	10.567
Level 4		C3 H7 O6 P3 S3	327.862	0.987
Level 4		C3 H8 Cl2 F N4 O P	235.97884	29.346
Level 4		C3 H8 Cl2 F6 O P2 S	337.9059	1.038
Level 4		C3 H8 Cl2 Si	141.97687	0.948
Level 4		C3 H8 F6 N O9 P3 S3	504.84786	21.461
Level 4		C3 H9 Br O P2 S	233.90382	0.956
Level 4		C3 H9 Cl2 O P3	223.9249	0.941
Level 4		C3 H9 F N2 O3 P2 S2	265.95088	1.1
Level 4		C3 H9 F2 O18 P3 S2	527.84111	1.036
Level 4		C3 H9 N3	87.07956	1.319
Level 4		C30 H30 F2 O	444.22799	15.502
Level 4		C30 H31 F N4 O4	530.23242	18.123
Level 4		C30 H38 F3 N2 O4 P S	610.22376	13.946
Level 4		C30 H43 N O11	593.28371	16.791
Level 4		C30 H44 F4 N O3 P	573.29958	9.263
Level 4		C30 H55 N O5 P2	571.35701	17.254
Level 4		C30 H58 N2 O9	590.41432	8.267
Level 4		C30 H61 N O13	643.41466	18.567
Level 4		C30 H63 N O10	597.44587	16.792
Level 4		C30 H74 F4 N2 O14	762.50792	19.351
Level 4		C31 H56 N8 O7	652.42649	12.118
Level 4		C31 H60 N3 O4 P S	601.40367	18.507
Level 4		C31 H61 F8 N2 O6 P	740.41489	11.644

Identification level	Name	Formula	Calc. MW	RT [min]
Level 4		C31 H71 N7 O S4	685.46106	19.42
Level 4		C32 H26 N2 O2	470.20068	18.832
Level 4		C32 H47 N O12	637.30986	16.883
Level 4		C32 H47 N O12	637.30975	16.968
Level 4		C32 H57 F5 N O3 P	629.39877	18.065
Level 4		C32 H68 F6 O10	726.47194	13.327
Level 4		C32 H81 N7 O17	835.56945	20.331
Level 4		C33 H30 F N	459.2373	13.531
Level 4		C33 H30 F N3 O	503.23691	12.257
Level 4		C33 H30 N4 O2	257.11871	18.532
Level 4		C33 H38 F N3 O	511.29953	8.155
Level 4		C33 H42 N O P	499.29965	15.21
Level 4		C33 H52 O5 P2	590.32811	18.078
Level 4		C33 H61 F N3 O5 P	629.43497	19.034
Level 4		C33 H67 N O14	701.45576	19.364
Level 4		C34 H51 F2 N5 O2 P2 S	693.32048	13.043
Level 4		C34 H66 F5 N4 O3 P3 S	798.39956	13.499
Level 4		C34 H69 F6 N8 O2 P	766.51929	11.009
Level 4		C34 H83 F2 O10 P3 S	814.48822	12.551
Level 4		C34 H93 F2 N6 O11 P3	892.60814	20.713
Level 4		C34 H94 F N9 O10	807.71079	0.891
Level 4		C35 H49 F3 N2 O6 P2	712.30139	5.494
Level 4		C35 H53 F O10	652.36238	10.968
Level 4		C35 H58 F2 N10 O8	784.44123	11.944
Level 4		C35 H59 N O5 Si2	629.39134	17.725
Level 4		C35 H61 N5 O2 S	615.45589	19.388
Level 4		C35 H63 F5 N O4 P	687.4405	18.772
Level 4		C35 H72 F11 N2 O4 P3	886.45181	13.884
Level 4		C35 H73 F N2 O14	764.50435	13.63
Level 4		C35 H73 F12 O4 P	816.5048	11.828
Level 4		C35 H78 F8 O4 P2	776.52433	19.749
Level 4		C35 H91 F9 N8 O9	938.67655	21.385
Level 4		C36 H67 F N3 O6 P	687.47683	19.629
Level 4		C36 H72 F8 O P2	734.4924	12.401
Level 4		C36 H73 F10 N3 O5	817.53959	20.373
Level 4		C36 H73 N O15	759.49761	19.904
Level 4		C36 H74 S2	570.52342	17.521
Level 4		C36 H81 F N2 O12 P2	814.52494	13.794
Level 4		C36 H81 F4 N4 O12 P S	900.52458	13.574
Level 4		C36 H82 F2 N2 O13 S	820.55086	19.884
Level 4		C37 H62 F2 N10 O9	828.46741	12.219
Level 4		C37 H69 F N4 O15	828.47475	12.748
Level 4		C37 H83 F12 N3 O P2	875.58194	20.786
Level 4		C38 H54 O7	622.38862	10.627

Identification level	Name	Formula	Calc. MW	RT [min]
Level 4		C38 H59 F S	566.43147	27.782
Level 4		C38 H60 F3 N9 O4	763.47268	17.65
Level 4		C38 H66 F5 N7 O S3	827.44328	17.132
Level 4		C38 H71 F2 N4 O6 P	748.5084	12.155
Level 4		C38 H71 F5 N10 P2 S	856.4979	13.369
Level 4		C38 H73 F N2 O6 P2	734.49285	12.209
Level 4		C39 H33 F3	558.25408	18.314
Level 4		C39 H50 F8	670.37965	8.302
Level 4		C39 H62 F8 N P	727.44891	16.485
Level 4		C39 H63 F O9	694.44597	15.021
Level 4		C39 H80 F3 N3 O3 P2	757.56236	28.718
Level 4		C39 H93 F15 N8 O12	1150.66733	14.203
Level 4		C4 Cl6 Hg	459.78449	0.922
Level 4		C4 F4 O	139.98828	0.958
Level 4		C4 H Cl F3 O2 P S	235.90747	0.945
Level 4		C4 H Cl2 F10 O9 P3 S	577.77715	1.017
Level 4		C4 H Cl2 F4 N2 O5 P3 S2	459.78511	1.051
Level 4		C4 H F N3 P3 S2	266.8807	1.051
Level 4		C4 H F O13 P2 S	369.85956	0.922
Level 4		C4 H F O14 P2	353.88212	0.897
Level 4		C4 H F10 O3 P3 S3	475.81395	1.047
Level 4		C4 H F2 N O2 P2 S2	258.88963	0.978
Level 4		C4 H F2 N3 O5 S3	304.90462	1.046
Level 4		C4 H F3 N3 O2 P3 S3	368.83959	1
Level 4		C4 H F4 N P2 S	232.92407	0.935
Level 4		C4 H F4 O P S2	235.91414	1.214
Level 4		C4 H F4 O12 P3 S3	505.77613	1.05
Level 4		C4 H F5 N O4 P3 S2	378.84838	1.018
Level 4		C4 H F5 N2 O5 P2 S2	377.87228	1.064
Level 4		C4 H F5 N2 O6 P2 S3	425.83907	1.029
Level 4		C4 H F6 N2 O2 P S3	349.88423	1.303
Level 4		C4 H F6 O6 P3 S2	415.83351	0.943
Level 4		C4 H N2 O3 P3 S	249.89183	0.981
Level 4		C4 H N6 P	163.99973	1.029
Level 4		C4 H P3 S3	237.84605	0.966
Level 4		C4 H10 N3 P S	163.03307	0.916
Level 4		C4 H10 N6 O2	174.08651	1.317
Level 4		C4 H10 O2	90.06821	5.802
Level 4		C4 H10 O2	90.06804	6.275
Level 4		C4 H11 N O2 S	137.05089	1.129
Level 4		C4 H11 P S2	154.004	0.896
Level 4		C4 H12 F3 N2 O3 P	224.05406	7.916
Level 4		C4 H12 N2	88.09984	1.058
Level 4		C4 H13 F N3 O P3 S	262.99756	29.348

Identification level	Name	Formula	Calc. MW	RT [min]
Level 4		C4 H14 Cl O4 P3 S	285.95125	13.899
Level 4		C4 H14 F N O6	191.08069	7.923
Level 4		C4 H14 F2 N8 O	228.12643	7.382
Level 4		C4 H14 F3 N3 S	193.08568	8.962
Level 4		C4 H14 F3 N9 O5	325.10735	8.13
Level 4		C4 H14 F5 N3	199.11096	12.32
Level 4		C4 H2 Cl F8 P3 S	361.8658	12.558
Level 4		C4 H2 Cl4 O2	221.88155	1.169
Level 4		C4 H2 F N9 P2 S3	352.90589	1.148
Level 4		C4 H2 F O3 P S3	243.88877	1.122
Level 4		C4 H2 F2 N O12 P3 S3	482.79216	1.071
Level 4		C4 H2 F2 O3 P2 S2	261.88919	1.024
Level 4		C4 H2 F2 O8 P2 S3	373.83594	1.026
Level 4		C4 H2 F4 N2 O9 P2 S	391.88924	1.039
Level 4		C4 H2 N O11 P3 S	364.85668	0.866
Level 4		C4 H2 N4 S2	169.97187	0.951
Level 4		C4 H2 O11 P2 S3	383.82329	0.938
Level 4		C4 H3 Br Cl F4 N4 O8 P	455.8495	1.047
Level 4		C4 H3 Br F6	243.93266	0.935
Level 4		C4 H3 Cl F3 O4 P	237.94064	0.947
Level 4		C4 H3 Cl N5 O3 P	234.96631	0.953
Level 4		C4 H3 F N2 O6 P2 S2	319.8893	1.081
Level 4		C4 H3 F N2 O8 P2 S2	351.87914	0.989
Level 4		C4 H3 F2 N4 O2 P3 S2	333.88816	1.034
Level 4		C4 H3 F3 N O7 P3 S2	390.85164	1.019
Level 4		C4 H3 F3 O2	140.00888	0.948
Level 4		C4 H3 O10 P3 S3	399.81056	1.132
Level 4		C4 H3 O2 P3 S3	271.8511	1.089
Level 4		C4 H3 O5 P S3	257.8881	1.169
Level 4		C4 H4 Cl2	121.96937	0.953
Level 4		C4 H4 F5 N O3 P2 S3	366.87494	1.044
Level 4		C4 H4 F5 N2 P S2	269.9475	0.926
Level 4		C4 H4 N5 O4 P3 S3	374.86455	1.082
Level 4		C4 H5 Cl N4	144.02037	4.268
Level 4		C4 H5 Cl N4 O	160.0152	2.726
Level 4		C4 H5 F N3 O16 P3 S3	558.80284	1.029
Level 4		C4 H5 F2 N2 O13 P3 S3	515.81346	1.021
Level 4		C4 H5 F2 N2 O5 P3 S2	355.88204	1.075
Level 4		C4 H5 F3 N9 O5 P	347.01033	29.358
Level 4		C4 H5 F3 S	142.00626	0.939
Level 4		C4 H5 N O6 P2 S2	288.90325	1.049
Level 4		C4 H5 N2 O4 P3	237.94612	1.128
Level 4		C4 H5 N3 O	111.04323	1.158
Level 4		C4 H5 N5 O4 P2	248.98198	0.948

Identification level	Name	Formula	Calc. MW	RT [min]
Level 4		C4 H5 O10 P3 S3	401.82584	1.139
Level 4		C4 H6 F O18 P3 S2	517.81939	1.074
Level 4		C4 H6 F7 N10 O14 P S	613.94115	22.662
Level 4		C4 H6 N2 O S2	161.99207	4.656
Level 4		C4 H6 N2 O10 P2 S3	399.86667	1.109
Level 4		C4 H6 N2 S2	145.9973	9.308
Level 4		C4 H6 N3 O P S3	238.9415	0.942
Level 4		C4 H6 N6 O2	170.05567	4.689
Level 4		C4 H6 O5 P	164.99496	1.185
Level 4		C4 H7 Cl N2 O4 S	213.98118	0.918
Level 4		C4 H7 Cl2 P3 S4	345.80284	1.008
Level 4		C4 H7 F2 N2 O12 P3 S3	501.83353	1.062
Level 4		C4 H7 N2 O2 P3 S	239.94412	0.844
Level 4		C4 H7 N2 P3 S3	271.89837	1.094
Level 4		C4 H7 N3 O2	129.05382	1.173
Level 4		C4 H7 N5 O	141.06502	1.294
Level 4		C4 H7 N7 O	169.07145	2.185
Level 4		C4 H8 N4 O	128.06974	1.158
Level 4		C4 H8 N6 O	156.07634	4.964
Level 4		C4 H8 N6 O	156.07628	5.805
Level 4		C4 H8 N6 O	156.07628	6.29
Level 4		C4 H8 S	88.0346	1.953
Level 4		C4 H9 F N2 O S	152.0418	1.036
Level 4		C4 H9 N2 P S2	179.99467	0.93
Level 4		C4 H9 N3 O2 P2	193.01754	9.062
Level 4		C4 H9 N7	155.09189	1.239
Level 4		C40 H107 F N4 O9 P2	868.74974	24.093
Level 4		C40 H66 O7 S	690.45168	10.764
Level 4		C40 H78 N2	586.61614	18.103
Level 4		C40 H78 N2 O14 P2	872.49362	12.475
Level 4		C40 H85 F N2 O10 P2	834.56597	20.237
Level 4		C40 H89 N3 P2 S	705.62488	25.994
Level 4		C41 H49 N3 O2	615.38323	17.328
Level 4		C41 H89 N10 O4 P3	878.62878	20.993
Level 4		C42 H71 F O11	770.49774	13.568
Level 4		C42 H78 F10 N8 P2	946.56758	13.243
Level 4		C42 H87 F2 N4 O18 P	1004.57274	13.137
Level 4		C42 H95 F4 N5 O9 P2	951.65409	21.102
Level 4		C43 H62 O6	674.45542	10.833
Level 4		C43 H70 F2 O3 P2 S2	798.42057	10.722
Level 4		C43 H73 F10 O3 P	858.51476	12.8
Level 4		C43 H73 F5 N4 O11	916.51987	12.713
Level 4		C43 H79 F8 N6 O P	878.59274	20.336
Level 4		C44 H103 F2 N7 O6	863.79381	25.876

Identification level	Name	Formula	Calc. MW	RT [min]
Level 4		C44 H54 F16 N P	931.3737	17.12
Level 4		C44 H61 N9 O S	763.47184	14.367
Level 4		C44 H64 F2 N O4 P	739.45638	12.665
Level 4		C44 H64 F3 N2 O2 P3 S	834.38555	8.223
Level 4		C44 H77 N5 O6 S	803.55993	20.603
Level 4		C45 H107 F2 O14 P3 S	1034.65684	14.695
Level 4		C45 H51 F N8 O8	850.38075	5.903
Level 4		C45 H81 F2 N8 O6 P3	960.54644	12.933
Level 4		C45 H84 F16 N6 O4	1076.62986	14.271
Level 4		C46 H104 F11 N2 O13 P S	1164.6821	14.571
Level 4		C46 H105 F3 N2 O4 P2	868.75017	24.884
Level 4		C46 H64 F9 N3 O3 P2	939.42838	2.722
Level 4		C46 H93 F5 N10 O6 S	1008.69242	21.435
Level 4		C47 H66 N4 O6	782.49798	15.389
Level 4		C47 H76 F11 N4 O4 P3 S	1094.46208	6.236
Level 4		C47 H80 F5 N6 O P S	902.57767	14.191
Level 4		C47 H83 F5 N4 O12	990.59394	13.443
Level 4		C48 H110 F10 O6 P2 S	1066.73429	21.74
Level 4		C48 H75 F N4 O11	902.54096	13.03
Level 4		C49 H88 F N8 O7 P	950.65026	21.08
Level 4		C5 H Cl F2 N O P3	256.89249	0.954
Level 4		C5 H F N3 O2 P3 S	278.89833	1.025
Level 4		C5 H F O13 P2 S3	445.80409	1.047
Level 4		C5 H F O6 P2 S3	333.83897	0.899
Level 4		C5 H F10 O9 P3 S	519.8394	1.067
Level 4		C5 H F12 N2 O3 P3 S	489.87294	1.113
Level 4		C5 H F2 O5 P S2	273.89655	0.86
Level 4		C5 H F6 N2 P3 S3	391.84187	1.05
Level 4		C5 H N6 P	175.99968	0.91
Level 4		C5 H N7 O2 P2 S3	348.88274	1.035
Level 4		C5 H O9 P3 S	329.85522	0.92
Level 4		C5 H10 Cl O2 P3	229.95874	0.867
Level 4		C5 H10 F2 O S	156.04241	7.197
Level 4		C5 H10 F3 N5 O4	261.06834	15.103
Level 4		C5 H10 N2 O2	130.07417	1.079
Level 4		C5 H10 N2 O2	130.07425	5.693
Level 4		C5 H10 N2 O3	146.06905	1.371
Level 4		C5 H10 N2 O4	162.06409	11.462
Level 4		C5 H10 N4 O	142.08543	1.219
Level 4		C5 H10 N6 O2	186.08642	1.554
Level 4		C5 H11 Br N P S2	258.92548	1.182
Level 4		C5 H11 F2 N S2	187.03029	6.686
Level 4		C5 H11 N7 O	185.1024	1.363
Level 4		C5 H11 N8 O2 P3 S	339.99388	6.87

Identification level	Name	Formula	Calc. MW	RT [min]
Level 4		C5 H12 Cl N2 O2 P	198.03255	6.978
Level 4		C5 H12 Cl6 F2 O6	415.87297	0.865
Level 4		C5 H12 N O4 P	181.05047	3.974
Level 4		C5 H12 N3 P S	177.04878	29.342
Level 4		C5 H13 F N5 P	193.08944	12.442
Level 4		C5 H13 F3 O2 S	194.05818	10.344
Level 4		C5 H13 N O3	135.08953	1.054
Level 4		C5 H13 N2 P S	164.05347	0.966
Level 4		C5 H14 F2 N6 S	228.09732	7.767
Level 4		C5 H15 F4 N5 O	237.12093	8.216
Level 4		C5 H16 F3 N O S	195.08988	6.614
Level 4		C5 H16 N9 O2 P	265.1172	7.239
Level 4		C5 H17 Cl N6 S	228.09293	2.339
Level 4		C5 H17 F2 N O4	193.11228	1.037
Level 4		C5 H17 F2 N O4	193.11231	1.203
Level 4		C5 H17 F4 N5	223.14212	10.213
Level 4		C5 H17 N2 O8 P	264.0721	5.346
Level 4		C5 H17 N3 O5 S	231.08967	7.197
Level 4		C5 H2 Cl F2 O2 P S2	261.88938	1.044
Level 4		C5 H2 Cl F2 O4 P S2	293.87871	12.556
Level 4		C5 H2 Cl N10 O8 P	395.94903	29.35
Level 4		C5 H2 Cl N4 O P	199.9656	0.916
Level 4		C5 H2 Cl2 N2 O	175.95482	1.098
Level 4		C5 H2 Cl3 F5 N8 O2 P2 S4	595.76445	1.062
Level 4		C5 H2 F N O3 P2 S4	332.83678	1.023
Level 4		C5 H2 F2 O2 S	163.97454	0.952
Level 4		C5 H2 F3 N2 O2 P3 S3	367.84456	1.057
Level 4		C5 H2 F3 N2 O8 P S2	369.89421	1.04
Level 4		C5 H2 F4 N2	166.01532	0.962
Level 4		C5 H2 F4 N2	166.01538	1.311
Level 4		C5 H2 F4 N4 O3 P2 S2	367.89797	0.904
Level 4		C5 H2 F5 O3 P3 S3	393.82949	0.902
Level 4		C5 H2 F7 N O4 P2 S3	430.85039	1.059
Level 4		C5 H2 N O13 P3 S2	440.81892	1.054
Level 4		C5 H2 N O2 P3 S2	264.87434	0.989
Level 4		C5 H2 N8 O3 P2 S3	379.88897	1.199
Level 4		C5 H3 Cl F O5 P3 S5	449.74678	0.947
Level 4		C5 H3 Cl F2 O3 S4	311.86119	1.088
Level 4		C5 H3 Cl3 F8 N2 S5	507.78359	1.014
Level 4		C5 H3 F2 N2 O3 P S2	271.92926	0.998
Level 4		C5 H3 F5 N3 O P3 S3	404.85716	1.031
Level 4		C5 H3 F6 O14 P3 S	525.83548	1.031
Level 4		C5 H3 N O4 S2	204.9504	1.297
Level 4		C5 H4 Cl F5 N3 O P	282.97011	0.925

Identification level	Name	Formula	Calc. MW	RT [min]
Level 4		C5 H4 F2 O3 S	181.98512	0.951
Level 4		C5 H4 F3 N2 O2 P3 S3	369.85994	1.332
Level 4		C5 H4 N O4 S2	205.95882	0.986
Level 4		C5 H4 O7 S3	271.91188	1.058
Level 4		C5 H5 Cl F4 S3	271.91803	1.015
Level 4		C5 H5 F4 O P S	219.97373	1.04
Level 4		C5 H5 N O7 P2 S2	316.89819	1.02
Level 4		C5 H5 N3	107.04827	1.283
Level 4		C5 H5 N5	135.05471	5.732
Level 4		C5 H5 N5 O	151.04939	4.397
Level 4		C5 H6 F3 N2 O9 P3 S3	483.84004	1.06
Level 4		C5 H6 N2	94.05298	1.359
Level 4		C5 H6 N2 O2	126.04294	5.349
Level 4		C5 H6 O11 P2 S3	399.85472	1.04
Level 4		C5 H6 O5 P2 S2	271.91321	1.093
Level 4		C5 H6 S3 Se2	321.79535	0.988
Level 4		C5 H7 F2 N7 S	235.04575	7.728
Level 4		C5 H7 F3 O4	188.03022	12.32
Level 4		C5 H8 F3 N5	195.07315	1.282
Level 4		C5 H8 N2	96.0687	1.381
Level 4		C5 H8 N6 O2	184.07108	6.179
Level 4		C5 H9 N3 O	127.07447	1.375
Level 4		C5 H9 N3 O	127.07447	1.54
Level 4		C5 H9 N3 O2	143.06942	1.231
Level 4		C5 H9 O4 P	164.02362	4.666
Level 4		C5 N2 S4	215.8948	0.931
Level 4		C50 H110 F3 N O2 P2 S	907.76848	25.171
Level 4		C50 H110 F3 N O2 P2 S	907.76848	25.303
Level 4		C50 H90 O14 S	946.60444	14.371
Level 4		C51 H86 N10 O4 S	934.6551	21.274
Level 4		C52 H112 F N P2 S	863.79411	28.012
Level 4		C52 H76 N4 O4	820.58656	20.594
Level 4		C52 H89 F6 N10 O3 P S	1078.64671	13.802
Level 4		C52 H97 F9 N8 O3	1052.7545	21.97
Level 4		C53 H79 F6 N10 O3 P	1048.59892	13.329
Level 4		C54 H125 F4 N4 O12 P3 S2	1254.78803	15.352
Level 4		C55 H100 O6	856.75112	26.209
Level 4		C55 H101 N5 O2	863.79552	26.036
Level 4		C55 H81 F N4 O7 S2	992.55307	8.813
Level 4		C56 H59 F N2 O4	842.44662	11.07
Level 4		C57 H101 F3 O4	906.76474	23.862
Level 4		C57 H87 N2 O6 P3	988.57779	13.949
Level 4		C57 H94 F N2 O3 P S	936.6707	21.354
Level 4		C58 H94 F3 N6 O10 P	1122.67209	13.967

Identification level	Name	Formula	Calc. MW	RT [min]
Level 4		C6 H Cl N O5 P3	294.876	1.046
Level 4		C6 H Cl2 N2 P3	263.87276	1.18
Level 4		C6 H F2 N4 O4 P3 S2	387.86179	1.065
Level 4		C6 H F2 P3	203.92592	1.057
Level 4		C6 H F3 N2 O3	205.99391	1.013
Level 4		C6 H F3 N2 P2 S3	315.87306	1.153
Level 4		C6 H F7 O4 P2 S3	427.84017	1.012
Level 4		C6 H O4 P3	229.90886	0.963
Level 4		C6 H O6 P3 S2	325.84336	0.971
Level 4		C6 H P3 S2	229.87362	0.948
Level 4		C6 H10 Cl F5 O14	435.96794	9.126
Level 4		C6 H10 N2 O2	142.07422	2.865
Level 4		C6 H10 N2 O2	142.07425	9.767
Level 4		C6 H10 N6 O	182.09175	6.117
Level 4		C6 H10 N6 O3	214.08171	6.187
Level 4		C6 H10 N6 O3	214.08164	6.765
Level 4		C6 H10 O2	114.06812	8.313
Level 4		C6 H10 O2	114.06807	8.5
Level 4		C6 H10 O3	130.06303	8.725
Level 4		C6 H11 F N O6 P	243.03115	1.741
Level 4		C6 H11 F O S	150.0517	1.032
Level 4		C6 H11 F3 O	156.07641	6.101
Level 4		C6 H11 N O S	145.05625	7.567
Level 4		C6 H11 N O S2	177.02835	9.32
Level 4		C6 H11 N O2 S	161.05093	1.591
Level 4		C6 H11 N O4	161.06873	1.542
Level 4		C6 H11 N3 O	141.09025	2.654
Level 4		C6 H11 N3 O3	173.08	1.244
Level 4		C6 H11 N9	209.11378	2.172
Level 4		C6 H11 N9 O2	241.10343	2.533
Level 4		C6 H12 F N O4	181.07429	12.523
Level 4		C6 H12 N2	112.1	1.091
Level 4		C6 H12 N2 O	128.09488	1.321
Level 4		C6 H12 N2 O2	144.09009	5.883
Level 4		C6 H12 N2 O2	144.09013	9.536
Level 4		C6 H12 N4 O	156.10117	1.183
Level 4		C6 H12 N6 O	184.10695	1.815
Level 4		C6 H12 N8 O2 S	260.07969	10.214
Level 4		C6 H12 O2	116.0838	5.799
Level 4		C6 H12 O2	116.08372	6.284
Level 4		C6 H12 O2	116.08374	7.891
Level 4		C6 H12 O2	116.08377	8.06
Level 4		C6 H12 O2	116.08374	8.146
Level 4		C6 H12 O2	116.08374	8.275

Identification level	Name	Formula	Calc. MW	RT [min]
Level 4		C6 H12 O2	116.08374	8.7
Level 4		C6 H12 O2	116.08375	8.938
Level 4		C6 H12 O2	116.08374	9.107
Level 4		C6 H12 O2	116.08375	9.403
Level 4		C6 H12 O2	116.08374	9.571
Level 4		C6 H12 O2	116.08375	9.718
Level 4		C6 H12 O2	116.08375	10.882
Level 4		C6 H12 O3	132.07865	5.542
Level 4		C6 H12 O3	132.07865	7.528
Level 4		C6 H12 O3	132.07863	8.273
Level 4		C6 H12 O4	148.07359	4.923
Level 4		C6 H12 O4	148.07346	8.112
Level 4		C6 H12 O4	148.07358	11.721
Level 4		C6 H12 O5	164.06846	10.843
Level 4		C6 H13 Cl2 N O2	201.0322	1.44
Level 4		C6 H13 F N3 P S2	241.02651	9.932
Level 4		C6 H13 F2 N3 S	197.0799	5.246
Level 4		C6 H13 F3 O3 S2	254.02508	5.536
Level 4		C6 H13 N O S2	179.04398	10.7
Level 4		C6 H13 N O5	161.06882	1.717
Level 4		C6 H14 F N O5 S	231.05672	7.733
Level 4		C6 H14 F N5 O5	255.09746	16.247
Level 4		C6 H14 N2 O2	146.10542	1.305
Level 4		C6 H15 F11 N10 S	468.10225	19.269
Level 4		C6 H15 F3 N10 S	316.11522	9.767
Level 4		C6 H15 N S2	165.06503	1.609
Level 4		C6 H15 O7 P	230.05541	2.234
Level 4		C6 H16 F N O8	249.0864	6.742
Level 4		C6 H16 F N2 P S	198.07526	1.503
Level 4		C6 H16 F2 N2 O4 S	250.07916	8.729
Level 4		C6 H16 F2 N2 S	186.10044	1.126
Level 4		C6 H16 F2 N3 O5 P	279.07891	13.489
Level 4		C6 H16 F3 N S	191.09481	11.025
Level 4		C6 H16 F5 N O2	229.11032	6.037
Level 4		C6 H17 F N2 O4 S2	264.06078	8.324
Level 4		C6 H17 F3 N O3 P	239.09049	5.454
Level 4		C6 H17 F4 N5 O	251.137	1.716
Level 4		C6 H17 N O3	151.12098	5.8
Level 4		C6 H17 N4 O2 P S2	272.05217	7.109
Level 4		C6 H17 N9 O3	263.14472	10.175
Level 4		C6 H18 Cl F N2 O2 S4	331.99303	29.359
Level 4		C6 H18 F N O7	235.107	2.924
Level 4		C6 H18 F5 N O2	231.12596	17.446
Level 4		C6 H18 N4 S2	210.09735	1.713

Identification level	Name	Formula	Calc. MW	RT [min]
Level 4		C6 H18 S2 Sn3	513.79296	1.006
Level 4		C6 H19 F O2 S2	206.08066	8.798
Level 4		C6 H19 F2 N O S	191.11564	1.375
Level 4		C6 H19 F4 N5	237.15739	10.844
Level 4		C6 H19 N7 S	221.14158	9.355
Level 4		C6 H2 Cl F2 O P3	255.89723	1.103
Level 4		C6 H2 F N3 O P2 S2	276.90989	1.06
Level 4		C6 H2 F2 N O8 P3 S	378.86849	0.957
Level 4		C6 H2 F2 P2 S3	269.87628	1.063
Level 4		C6 H2 F3 N O15 S	416.90954	0.862
Level 4		C6 H2 F4 N2 O	194.01032	1.18
Level 4		C6 H2 N4 O P2 S3	303.88674	1.049
Level 4		C6 H20 F N6 O2 P	258.13687	16.155
Level 4		C6 H20 F3 N2 O4 P S	304.084	7.124
Level 4		C6 H20 F8	244.14372	18.983
Level 4		C6 H20 N O6 P	233.1028	10.775
Level 4		C6 H20 N10 O3	280.17128	10.479
Level 4		C6 H21 F2 N O S	193.13136	1.239
Level 4		C6 H21 F2 N S	177.13633	2.022
Level 4		C6 H21 F3 N3 O3 P	271.1278	1.621
Level 4		C6 H21 F5 O	204.15151	18.989
Level 4		C6 H21 N3 O P2	213.11565	16.304
Level 4		C6 H21 N3 P2	197.12065	18.797
Level 4		C6 H3 Br F6 N O6 P3	470.82453	1.035
Level 4		C6 H3 Cl F3 N O5	260.96454	0.847
Level 4		C6 H3 F O2 S2	189.95549	0.946
Level 4		C6 H3 F2 O5 P3 S	317.8883	0.883
Level 4		C6 H3 F3 O4 P2 S2	321.89	1.081
Level 4		C6 H3 F5 N O9 P3	420.89446	1.245
Level 4		C6 H3 N3 O P2 S3	290.89171	1.249
Level 4		C6 H3 N4 O7 P3 S2	399.86546	1.073
Level 4		C6 H4 Br Cl F2 N2	255.9222	1.068
Level 4		C6 H4 Cl3 F O8 S	359.86854	12.574
Level 4		C6 H4 F11 P S	347.95972	5.587
Level 4		C6 H4 F3 N O7 P2 S2	384.88553	1.012
Level 4		C6 H4 F5 N S2	248.97061	1.243
Level 4		C6 H4 F7 O4 P3 S3	461.83743	1.026
Level 4		C6 H4 F7 O6 P3	397.91091	1.227
Level 4		C6 H4 I N O	232.93334	0.944
Level 4		C6 H5 Cl Mg	135.99337	0.954
Level 4		C6 H5 Cl N5 O2 P	244.98704	0.91
Level 4		C6 H5 Cl2 F N O P S2	290.89162	1.142
Level 4		C6 H5 F5 N2 P2	261.98479	1.122
Level 4		C6 H5 I O4 S	299.89552	0.995

Identification level	Name	Formula	Calc. MW	RT [min]
Level 4		C6 H6 Cl F4 N4 O7 P S4	515.84807	12.564
Level 4		C6 H6 Cl N4 O2 P	231.99175	0.917
Level 4		C6 H6 N10 O	234.07293	6.094
Level 4		C6 H6 N2 O2	138.04294	1.895
Level 4		C6 H6 N3 O5 P3 S3	388.86621	1.045
Level 4		C6 H6 N4 O	150.05424	5.266
Level 4		C6 H6 O2	110.03675	16.737
Level 4		C6 H7 F N O11 P S3	414.8901	1.191
Level 4		C6 H7 F N O7 P3 S3	412.85787	1.177
Level 4		C6 H7 N O S	141.02494	5.861
Level 4		C6 H7 N O2	125.04758	1.757
Level 4		C6 H7 N O2 S	157.01981	5.397
Level 4		C6 H7 N3 O2	153.05378	2.604
Level 4		C6 H7 N3 O2	153.05377	2.913
Level 4		C6 H7 N5 O	165.06503	1.74
Level 4		C6 H7 N5 O S	197.03734	4.561
Level 4		C6 H7 N5 S	181.04224	7.456
Level 4		C6 H8 Cl N4 O7 P	313.98231	29.353
Level 4		C6 H8 F3 N O2	183.05068	1.477
Level 4		C6 H8 N2 O2	140.0587	2.037
Level 4		C6 H8 N4 O2 S	200.03662	2.598
Level 4		C6 H8 N6 O2	196.07123	9.952
Level 4		C6 H8 O4 S3	239.95827	1.138
Level 4		C6 H9 Br3 O	333.82084	1.108
Level 4		C6 H9 F3 O2	170.05558	4.946
Level 4		C6 H9 N O3	143.05821	1.665
Level 4		C6 H9 N3 O	139.07449	1.415
Level 4		C6 H9 N3 O	139.07459	3.513
Level 4		C6 H9 N3 O	139.07458	4.027
Level 4		C6 H9 N5 O	167.08076	3.91
Level 4		C60 H111 F13 N2 O9 P2	1312.75585	14.271
Level 4		C60 H118 F13 O13 P S	1356.78214	14.403
Level 4		C62 H84 N8 O2 P2	1034.62037	13.627
Level 4		C62 H97 F7 N2 O3	1050.73874	21.893
Level 4		C64 H86 F O2 P	936.63525	20.745
Level 4		C64 H87 F8 N4 O4 P3 S	1252.55284	7.103
Level 4		C7 H Br F N2 O2 P3 S	367.81333	0.938
Level 4		C7 H Cl2 F N2 S	233.92271	12.569
Level 4		C7 H Cl2 O7 P3	359.83103	0.936
Level 4		C7 H F N4 O2 S3	287.92455	1.146
Level 4		C7 H F2 O P3	231.92073	0.849
Level 4		C7 H F2 O P3	231.9207	1.18
Level 4		C7 H F6 N3 O4 P2 S2	430.87865	1.029
Level 4		C7 H F7 P2 S5	439.80336	1.063

Identification level	Name	Formula	Calc. MW	RT [min]
Level 4		C7 H F9 N6 O8 P2	529.91874	0.962
Level 4		C7 H N4 O18 P3 S2	585.79416	17.869
Level 4		C7 H10 F2 S	164.04765	10.017
Level 4		C7 H10 F3 O3 P	230.03255	13.61
Level 4		C7 H10 N2 O	138.07926	1.225
Level 4		C7 H10 N2 O	138.07946	6.53
Level 4		C7 H10 N2 O S	170.05149	6.771
Level 4		C7 H10 N2 O2	154.07411	1.411
Level 4		C7 H10 N2 O2	154.07412	1.824
Level 4		C7 H10 N2 O2	154.07415	5.904
Level 4		C7 H10 N2 O2 S	186.0465	2.089
Level 4		C7 H10 N2 O3	170.06911	2.565
Level 4		C7 H10 N4 O3	198.0754	4.861
Level 4		C7 H10 O3	142.06308	5.945
Level 4		C7 H11 Br F2 S	243.97397	1.068
Level 4		C7 H11 F3 O2	184.07084	8.003
Level 4		C7 H11 F4 N9	297.10741	5.504
Level 4		C7 H11 N O2	141.07911	4.905
Level 4		C7 H11 N O2	141.07915	5.947
Level 4		C7 H11 N3	137.09523	1.209
Level 4		C7 H11 N3 O	153.09041	6.123
Level 4		C7 H11 N3 O S	185.0622	1.488
Level 4		C7 H11 N3 O2	169.08502	1.511
Level 4		C7 H11 N3 O3	185.07992	1.431
Level 4		C7 H11 N9 O2	253.10335	1.687
Level 4		C7 H12 F4 N4	228.09976	17.109
Level 4		C7 H12 N10 O	252.11925	1.888
Level 4		C7 H12 N2 O	140.09487	1.205
Level 4		C7 H12 N2 O	140.0948	1.936
Level 4		C7 H12 N2 O	140.0952	7.717
Level 4		C7 H12 N2 O2 S	188.0621	9.058
Level 4		C7 H12 N4 O2	184.09611	2.846
Level 4		C7 H12 N4 O3	200.09091	1.289
Level 4		C7 H12 N6 O	196.10731	5.505
Level 4		C7 H13 F2 N O S	197.06894	10.734
Level 4		C7 H13 F2 N S	181.07436	12.371
Level 4		C7 H13 N O2	143.09471	5.619
Level 4		C7 H13 N2 S	157.07956	5.798
Level 4		C7 H13 N3 O4	203.09059	1.068
Level 4		C7 H14 N6 O2	214.11791	6.13
Level 4		C7 H14 O3	146.09444	7.582
Level 4		C7 H14 O4	162.08928	5.486
Level 4		C7 H14 O4	162.08932	7.995
Level 4		C7 H15 Cl2 N2 O2 P	260.02476	11.63

Identification level	Name	Formula	Calc. MW	RT [min]
Level 4		C7 H15 F N2 O3	194.10613	11.155
Level 4		C7 H15 F N5 P S2	283.04906	12.039
Level 4		C7 H15 F2 N O S	199.08424	1.395
Level 4		C7 H15 F2 N S2	215.06185	11.304
Level 4		C7 H15 F2 N3 O S	227.09037	1.97
Level 4		C7 H15 F2 N3 O2 S	243.08532	1.631
Level 4		C7 H15 F9 N2	298.10993	9.188
Level 4		C7 H15 N O S3	225.03169	9.921
Level 4		C7 H15 N9 O2	257.13465	1.895
Level 4		C7 H15 N9 O2	257.13451	3.052
Level 4		C7 H16 F P S	182.06943	1.414
Level 4		C7 H16 F2 N2 O S	214.09523	1.678
Level 4		C7 H16 N2 O3	176.116	1.168
Level 4		C7 H17 F N2 O2 S2	244.07118	8.719
Level 4		C7 H17 F N4 O2 S	240.10621	2.534
Level 4		C7 H17 F O2 S2	216.06493	7.896
Level 4		C7 H17 N O2	147.12588	1.311
Level 4		C7 H17 N O2 S	179.09786	1.1
Level 4		C7 H17 N O4	179.11563	1.655
Level 4		C7 H18 F2 N O2 P S	249.0768	11.098
Level 4		C7 H18 F2 N6 S	256.12866	12.864
Level 4		C7 H18 F3 N O8	301.09858	9.929
Level 4		C7 H18 F4 N4	234.14674	9.615
Level 4		C7 H19 Cl F2 O2 S	240.07651	7.879
Level 4		C7 H19 F N10	262.17809	16.3
Level 4		C7 H19 F O2 S2	218.08055	11.571
Level 4		C7 H19 F O7	234.11162	12.922
Level 4		C7 H19 F3 N3 O2 P	265.11733	7.482
Level 4		C7 H19 F4 N5	249.15754	7.283
Level 4		C7 H19 N3	145.15792	1.137
Level 4		C7 H19 N3	145.15786	1.251
Level 4		C7 H19 N7 O8	329.12969	7.801
Level 4		C7 H2 Cl F2 N	172.98447	1.155
Level 4		C7 H2 Cl N2 O2 P3	273.90178	1.238
Level 4		C7 H2 F2 N O2 P3 S3	358.84252	1.23
Level 4		C7 H2 F2 N P	168.98974	0.936
Level 4		C7 H2 F2 N P S2	232.93338	0.991
Level 4		C7 H2 F2 O2 P2	217.95032	1.343
Level 4		C7 H2 F3 O15 P3 S2	539.79872	1.038
Level 4		C7 H2 F5 N2 P3 S	333.90715	1.294
Level 4		C7 H2 P2 S3	243.87885	1.11
Level 4		C7 H20 F N8 O P S	314.11905	14.435
Level 4		C7 H20 F P S	186.10079	9.537
Level 4		C7 H20 F2 O S	190.12037	10.667

Identification level	Name	Formula	Calc. MW	RT [min]
Level 4		C7 H20 F2 O S	190.12065	12.775
Level 4		C7 H20 F4 N4 O	252.15769	8.002
Level 4		C7 H20 N4 O S2	240.10765	2.58
Level 4		C7 H20 N9 O P	277.1527	10.711
Level 4		C7 H21 F O3 S2	236.09116	5.441
Level 4		C7 H22 F N O3 S2	251.10187	8.667
Level 4		C7 H22 F N5 O3	243.1709	7.917
Level 4		C7 H22 F3 N O4 S	273.12248	12.86
Level 4		C7 H22 F5 N O	231.16264	7.537
Level 4		C7 H23 F2 N O4 S	255.13163	4.59
Level 4		C7 H23 F4 N5 O	269.18376	5.483
Level 4		C7 H23 F4 O2 P	246.13703	11.64
Level 4		C7 H23 F5 O5	282.14652	17.34
Level 4		C7 H3 Br F4	241.93542	0.956
Level 4		C7 H3 Br2 F2 N O2	328.85082	1.024
Level 4		C7 H3 Cl2 N O5 S	282.9101	1.049
Level 4		C7 H3 Cl2 O8 P3	377.84151	0.932
Level 4		C7 H3 F O S2	185.96061	0.949
Level 4		C7 H3 F2 N O S3	250.93445	1.251
Level 4		C7 H3 F2 O4 P3	281.92117	1.058
Level 4		C7 H3 N6 P3 S2	327.90697	1.064
Level 4		C7 H4 Cl F2 P3	253.91803	0.941
Level 4		C7 H4 Cl N6 O6 P3 S2	459.85328	1.21
Level 4		C7 H4 Cl P S3	249.89047	1.224
Level 4		C7 H4 F2 O S	173.99529	0.938
Level 4		C7 H6	90.04677	5.389
Level 4		C7 H6 F2 N4 O	200.05099	9.827
Level 4		C7 H6 F2 O3 P2	237.97587	29.351
Level 4		C7 H6 N2 O S	166.02022	7.148
Level 4		C7 H6 N2 S	150.02515	9.637
Level 4		C7 H6 N4 O3	194.04428	6.373
Level 4		C7 H7 Cl2 O3 P3 S3	397.8141	1.055
Level 4		C7 H7 N O S	153.0249	8.917
Level 4		C7 H7 N O3	153.04247	1.524
Level 4		C7 H7 N3	133.06413	9.585
Level 4		C7 H7 N3 O	149.05887	1.835
Level 4		C7 H7 N3 O	149.059	9.953
Level 4		C7 H7 N3 O	149.05897	10.194
Level 4		C7 H7 N5 O	177.06513	4.872
Level 4		C7 H8 Br Cl8 F2 O5 P S	631.64944	0.937
Level 4		C7 H8 Cl N3	169.04074	6.99
Level 4		C7 H8 Cl N4 O2 P	246.00741	0.902
Level 4		C7 H8 N2 O2	152.05855	1.419
Level 4		C7 H8 N2 O2	152.05858	7.079

Identification level	Name	Formula	Calc. MW	RT [min]
Level 4		C7 H8 N4 O	164.06993	4.789
Level 4		C7 H8 N4 O2	180.0649	4.705
Level 4		C7 H8 N4 O3	196.05976	6.861
Level 4		C7 H8 O4	156.04242	5.766
Level 4		C7 H8 O4	156.04239	13.895
Level 4		C7 H9 F N3 O P	201.04632	2.057
Level 4		C7 H9 F3 O2	182.05586	9.246
Level 4		C7 H9 F4 N5	239.07954	7.811
Level 4		C7 H9 N O S	155.04054	7.624
Level 4		C7 H9 N O2	139.06336	2.487
Level 4		C7 H9 N O2	139.06337	3.687
Level 4		C7 H9 N O2 S	171.03549	8.704
Level 4		C7 H9 N O2 S	171.03537	9.561
Level 4		C7 H9 N O3	155.05836	1.866
Level 4		C7 H9 N O3	155.05831	6.438
Level 4		C7 H9 N O3 S	187.03021	1.832
Level 4		C7 H9 N3 O	151.0746	2.694
Level 4		C7 H9 N5	163.08587	5.091
Level 4		C7 H9 N5 O	179.08073	2.232
Level 4		C7 H9 N5 O	179.08078	6.965
Level 4		C7 H9 N5 O S	211.05303	6.12
Level 4		C8 H F2 N2 O P3 S2	335.87205	1.056
Level 4		C8 H N2 O3 P3 S	297.89222	1.079
Level 4		C8 H O9 P3	333.88359	1.077
Level 4		C8 H10 Cl N5	211.06251	7.812
Level 4		C8 H10 N2 O	150.07924	1.427
Level 4		C8 H10 N2 O2	166.07439	1.464
Level 4		C8 H10 N2 O2	166.07437	1.992
Level 4		C8 H10 N2 O2	166.07438	2.37
Level 4		C8 H10 N2 O2	166.0744	6.868
Level 4		C8 H10 N2 O2	166.07442	7.248
Level 4		C8 H10 N2 O2	166.07434	9.246
Level 4		C8 H10 N2 O3	182.06923	5.12
Level 4		C8 H10 N4 O2 S	226.05243	8.171
Level 4		C8 H10 N4 O2 S	226.05247	9.185
Level 4		C8 H10 N4 O3	210.07542	7.711
Level 4		C8 H10 N4 O3 S	242.0475	5.53
Level 4		C8 H10 N6 O	206.09152	1.713
Level 4		C8 H10 O3 S	186.03535	7.917
Level 4		C8 H10 O3 S	186.03534	8.839
Level 4		C8 H10 O3 S	186.03528	9.308
Level 4		C8 H10 O4	170.05798	7.785
Level 4		C8 H11 N O2	153.07906	3.81
Level 4		C8 H11 N O2	153.07924	7.411

Identification level	Name	Formula	Calc. MW	RT [min]
Level 4		C8 H11 N O2 S	185.05121	5.887
Level 4		C8 H11 N O2 S	185.05112	6.695
Level 4		C8 H11 N O3	169.07395	1.253
Level 4		C8 H11 N O3	169.07424	3.625
Level 4		C8 H11 N O3	169.0739	5.395
Level 4		C8 H11 N O3 S	201.04623	2.259
Level 4		C8 H11 N5 O	193.09653	6.295
Level 4		C8 H11 N5 O	193.09652	8.239
Level 4		C8 H12 F3 N3 O	223.093	3.392
Level 4		C8 H12 N10	248.12463	3.865
Level 4		C8 H12 N2 O2 S2	232.03405	5.259
Level 4		C8 H12 N2 O3	184.08472	1.417
Level 4		C8 H12 N2 O3	184.08493	3.516
Level 4		C8 H12 N2 O3 S2	248.02917	2.255
Level 4		C8 H12 N4 O	180.10085	2.353
Level 4		C8 H12 N4 O3	212.09112	5.523
Level 4		C8 H12 N4 O3	212.09114	6.097
Level 4		C8 H12 N6 O	208.10736	8.087
Level 4		C8 H12 N6 O2	224.10264	12.959
Level 4		C8 H12 O3 S	188.05102	7.205
Level 4		C8 H12 O4	154.06312	8.96
Level 4		C8 H12 O5	188.06866	6.53
Level 4		C8 H13 N O	139.09969	9.729
Level 4		C8 H13 N O2	155.09485	6.355
Level 4		C8 H13 N O2	155.09477	7.555
Level 4		C8 H13 N O2	155.0947	9.429
Level 4		C8 H13 N O3	171.08966	7.382
Level 4		C8 H13 N O4	209.06909	4.367
Level 4		C8 H13 N O4	209.06886	8.077
Level 4		C8 H13 N3	151.11088	1.297
Level 4		C8 H13 N3 O	167.10607	6.474
Level 4		C8 H13 N3 O	167.10604	7.757
Level 4		C8 H13 N3 O	167.10606	7.977
Level 4		C8 H13 N3 O2	183.1008	1.115
Level 4		C8 H13 N3 O4 S	247.06091	13.804
Level 4		C8 H14 N2 O3	186.10041	1.204
Level 4		C8 H14 N2 O7	250.07921	8.124
Level 4		C8 H14 N4 O2	198.11174	5.6
Level 4		C8 H15 F2 N O S	211.08444	5.505
Level 4		C8 H15 F2 O6 P	276.05726	10.582
Level 4		C8 H15 F4 N5 S	289.09863	9.783
Level 4		C8 H15 N O	141.11545	1.47
Level 4		C8 H15 N O S	173.08754	12.877
Level 4		C8 H15 N O2	157.11019	1.064

Identification level	Name	Formula	Calc. MW	RT [min]
Level 4		C8 H15 N O2	157.11037	6.755
Level 4		C8 H15 N O2	157.11053	7.186
Level 4		C8 H15 N O3	173.1056	7.56
Level 4		C8 H15 N O5	205.09498	1.859
Level 4		C8 H15 N3 O3	201.11152	2.455
Level 4		C8 H16 F N2 P S	222.07535	7.802
Level 4		C8 H16 F N3 O3	221.11677	12.728
Level 4		C8 H16 F2 O S2	230.06159	10.454
Level 4		C8 H16 F2 S	182.09439	6.667
Level 4		C8 H16 N2 O2	172.12122	1.089
Level 4		C8 H16 N2 O3	188.11618	1.199
Level 4		C8 H16 N2 O4	204.11103	11.459
Level 4		C8 H16 N4 O3	216.12247	5.79
Level 4		C8 H16 O3	160.10994	7.959
Level 4		C8 H16 O3	160.10988	8.134
Level 4		C8 H16 O3	160.10992	8.922
Level 4		C8 H16 O3	160.10992	9.101
Level 4		C8 H16 O3	182.09432	16.975
Level 4		C8 H16 O3	182.09436	17.156
Level 4		C8 H16 O3	182.09436	17.646
Level 4		C8 H16 O5	192.09987	6.752
Level 4		C8 H16 O5	192.09987	7.007
Level 4		C8 H17 Cl F N2 O2 P3 S	351.98966	12.621
Level 4		C8 H17 F2 N O2 S	229.09494	3.643
Level 4		C8 H17 F3 N3 P S	275.08294	10.256
Level 4		C8 H17 F7 N4 O	318.12919	6.062
Level 4		C8 H17 N O2	159.1259	4.127
Level 4		C8 H17 N O3	175.12069	1.361
Level 4		C8 H17 N O5	207.11195	5.469
Level 4		C8 H18 F O2 P3	258.05043	9.307
Level 4		C8 H18 F4 N10	330.16548	12.203
Level 4		C8 H18 F4 N2 O S3	330.05137	13.704
Level 4		C8 H18 N2 O S2	222.08651	8.043
Level 4		C8 H18 N2 O2	174.13686	1.092
Level 4		C8 H18 N8 O2 S2	322.10022	10.234
Level 4		C8 H18 N8 O2 S2	322.1003	11.152
Level 4		C8 H19 F N2 O8 S	322.08535	13.949
Level 4		C8 H19 F2 N S	199.12071	2.383
Level 4		C8 H19 F3 O8	300.10351	12.966
Level 4		C8 H19 N O	145.14677	5.001
Level 4		C8 H19 N O2	161.1415	1.266
Level 4		C8 H19 O5 P	226.09704	7.911
Level 4		C8 H2 Br F2 O P	261.89982	0.958
Level 4		C8 H2 F3 N2 O9 P3 S	451.86458	1.074

Identification level	Name	Formula	Calc. MW	RT [min]
Level 4		C8 H20 F N O8	277.11752	12.762
Level 4		C8 H20 F N O8	277.11753	12.878
Level 4		C8 H20 F N5 O	221.16548	8.632
Level 4		C8 H20 F2 N2 O8	310.11832	13.665
Level 4		C8 H20 F2 N2 S	214.13156	1.42
Level 4		C8 H20 F2 O2 S	218.1155	10.031
Level 4		C8 H20 F2 O3 S	234.11022	5.474
Level 4		C8 H20 N2 O3	192.14735	1.28
Level 4		C8 H20 N8 O S	276.14763	11.244
Level 4		C8 H21 Cl F3 N S	255.10317	10.79
Level 4		C8 H21 F N2 O6	260.13884	16.029
Level 4		C8 H21 F N3 P	209.14534	14.315
Level 4		C8 H21 F2 N O S	217.13137	8.045
Level 4		C8 H21 F3 N O P	235.13198	8.302
Level 4		C8 H21 F4 N4 P	280.14397	16.781
Level 4		C8 H21 F4 N5 O2	295.16289	2.691
Level 4		C8 H21 N O3	179.1521	6.28
Level 4		C8 H21 N3	159.17348	1.142
Level 4		C8 H21 N3	159.17349	1.248
Level 4		C8 H21 N3 S3	255.0896	14.908
Level 4		C8 H21 N7 S	247.15746	7.243
Level 4		C8 H22 F N O2 S2	247.10724	9.493
Level 4		C8 H22 F P S	200.1162	1.095
Level 4		C8 H22 F3 N2 P	234.14672	13.571
Level 4		C8 H22 F6 N10 O S	420.1606	6.861
Level 4		C8 H22 O S2	198.11187	9.413
Level 4		C8 H23 Cl F5 N3	291.15059	15.067
Level 4		C8 H23 F N2 O5	246.15926	17.527
Level 4		C8 H23 F O3 S	218.13454	14.624
Level 4		C8 H23 F2 N O2 P2 S	297.09001	9.186
Level 4		C8 H23 F2 N O3 S	251.13689	7.278
Level 4		C8 H23 F2 N O4	235.16034	8.142
Level 4		C8 H23 F2 N O4 P2	297.10736	4.98
Level 4		C8 H24 F N5 O4	273.18064	20.079
Level 4		C8 H24 F N7 O2 P2	331.14545	15.305
Level 4		C8 H24 F O P3	248.10237	11.284
Level 4		C8 H24 N O6 P	261.13395	15.042
Level 4		C8 H24 N2 O2 P2	242.13097	15.802
Level 4		C8 H24 N5 O4 P S	317.12986	14.431
Level 4		C8 H24 N9 O3 P	325.17391	9.867
Level 4		C8 H25 F2 N O2 S	237.15743	7.889
Level 4		C8 H25 F2 N O2 S	237.15743	8.454
Level 4		C8 H25 F2 N7 O3	305.19869	18.938
Level 4		C8 H25 F5 N2 O2 S	308.15663	4.767

Identification level	Name	Formula	Calc. MW	RT [min]
Level 4		C8 H25 N9 O3	295.20735	8.276
Level 4		C8 H26 F5 N3 O4	323.18437	14.611
Level 4		C8 H26 N2 O P2	228.15193	17.82
Level 4		C8 H26 N9 P	279.20473	13.394
Level 4		C8 H27 F N2 O10	330.16548	12.526
Level 4		C8 H27 F3 N P S	257.15502	7.772
Level 4		C8 H27 F3 N3 O2 P	285.17967	5.175
Level 4		C8 H27 N5 O2 S4	353.10466	10.362
Level 4		C8 H28 F10 O2 P2	408.13933	6.353
Level 4		C8 H28 F2 N9 P S	351.18945	12.898
Level 4		C8 H28 N2 O P2	230.1674	17.066
Level 4		C8 H28 N2 O P2	230.16729	17.303
Level 4		C8 H3 F N P S3	258.91457	1.09
Level 4		C8 H3 F7 N2 O13	467.95198	14.787
Level 4		C8 H4 O3	148.01621	19.391
Level 4		C8 H5 Cl2 N3 O4 S3	372.88061	0.863
Level 4		C8 H5 F N6 O3	252.04035	11.111
Level 4		C8 H5 F2 N6 P S5	413.88809	1.275
Level 4		C8 H6 Br F3 O2	269.95073	1.122
Level 4		C8 H6 Cl F2 O P S2	285.92537	0.932
Level 4		C8 H6 F2 N2 O	184.04499	8.398
Level 4		C8 H6 N2 O2	162.04327	2.148
Level 4		C8 H6 O4	166.02686	19.39
Level 4		C8 H6 O4	166.02637	23.906
Level 4		C8 H7 F2 P S	203.99709	1.087
Level 4		C8 H7 N O	133.05283	8.533
Level 4		C8 H7 N O S2	196.99708	11.895
Level 4		C8 H7 N O2 S	181.01986	8.777
Level 4		C8 H7 N3 O	161.05894	6.232
Level 4		C8 H8	104.06269	5.618
Level 4		C8 H8 F N4 P	210.04639	6.209
Level 4		C8 H8 N2 O	148.06386	6.253
Level 4		C8 H8 N2 O	148.06385	6.482
Level 4		C8 H8 N2 O	148.06385	9.327
Level 4		C8 H8 N2 O2 S	196.03052	3.75
Level 4		C8 H8 N4 O2	192.0623	2.516
Level 4		C8 H8 N4 O2	192.06489	7.039
Level 4		C8 H8 N4 O3	208.05984	6.235
Level 4		C8 H8 O3 S	184.01915	6.7
Level 4		C8 H9 N O2	151.0634	9.16
Level 4		C8 H9 N O2 S	183.03506	7.618
Level 4		C8 H9 N O3	167.05844	8.32
Level 4		C8 H9 N3 O2	179.06952	11.188
Level 4		C8 H9 N3 O3	195.06447	6.423

Identification level	Name	Formula	Calc. MW	RT [min]
Level 4		C8 H9 N3 O3	195.06446	6.971
Level 4		C8 H9 N5 O	191.08081	4.2
Level 4		C8 H9 N5 O	191.08079	6.293
Level 4		C8 H9 N5 O	191.08069	6.548
Level 4		C8 H9 N5 O2	207.0758	6.843
Level 4		C8 H9 N5 O2 S	239.04767	9.022
Level 4		C8 H9 N5 O3	223.07064	6.331
Level 4		C9 H Cl2 N O S	240.91509	0.956
Level 4		C9 H10 Cl N5 O2	255.05235	9.503
Level 4		C9 H10 N2 O2	178.07424	5.729
Level 4		C9 H10 N4 O2	206.08056	8.539
Level 4		C9 H10 N4 O3	222.07545	7.122
Level 4		C9 H10 N4 O3	222.07554	8.039
Level 4		C9 H10 O	134.07303	9.132
Level 4		C9 H11 F2 N5 O2	259.08804	7.777
Level 4		C9 H11 F3 O	192.07599	6.015
Level 4		C9 H11 N O2	165.07895	1.957
Level 4		C9 H11 N O2	165.079	13.573
Level 4		C9 H11 N O3	181.07387	5.093
Level 4		C9 H11 N O3	181.07426	8.556
Level 4		C9 H11 N O3 S	213.04601	6.756
Level 4		C9 H11 N O4	197.069	6.534
Level 4		C9 H11 N3 O2	193.08527	7.892
Level 4		C9 H11 N3 O3	209.0802	6.84
Level 4		C9 H11 N5 O3	237.0863	7.276
Level 4		C9 H11 N5 O3	237.08614	8.133
Level 4		C9 H11 N5 O3	237.08627	8.83
Level 4		C9 H11 O	135.08015	2.103
Level 4		C9 H12 Br3 N3 O S	446.82512	1.007
Level 4		C9 H12 F3 O4 P	272.04357	6.751
Level 4		C9 H12 N2 O	164.09519	3.304
Level 4		C9 H12 N2 O2	180.0899	4.66
Level 4		C9 H12 N2 O2	180.09001	8.917
Level 4		C9 H12 N2 O2	180.09003	9.209
Level 4		C9 H12 N2 O2	180.09019	9.529
Level 4		C9 H12 N2 O2	180.09038	9.657
Level 4		C9 H12 N2 O3	196.08485	4.157
Level 4		C9 H12 N4 O2 S	240.06811	10.576
Level 4		C9 H12 N4 O3	224.09121	7.906
Level 4		C9 H12 N4 O3 S	256.0631	6.859
Level 4		C9 H12 O3	190.06287	8.452
Level 4		C9 H13 F3 O4	242.07663	7.881
Level 4		C9 H13 N O S	183.07211	7.39
Level 4		C9 H13 N O S	183.07198	7.956

Identification level	Name	Formula	Calc. MW	RT [min]
Level 4		C9 H13 N O3	183.08943	1.465
Level 4		C9 H13 N O3	183.08998	9.381
Level 4		C9 H13 N O3 S	215.06188	7.098
Level 4		C9 H13 N O3 S2	247.03362	5.346
Level 4		C9 H13 N O5 S	247.0515	8.111
Level 4		C9 H13 N3 O	179.10602	5.402
Level 4		C9 H13 N3 S2	227.05577	7.587
Level 4		C9 H14 Cl N5	227.09405	12.338
Level 4		C9 H14 Cl N5 O	243.08899	13.421
Level 4		C9 H14 F2 S	192.07883	11.818
Level 4		C9 H14 N2 O2	182.10576	6.326
Level 4		C9 H14 N2 O2	182.10553	7.82
Level 4		C9 H14 N2 O3	198.10049	1.933
Level 4		C9 H14 N2 O3	198.10047	2.47
Level 4		C9 H14 N2 O3	198.10044	2.624
Level 4		C9 H14 N2 O3	198.10053	4.327
Level 4		C9 H14 O3	170.09434	12.764
Level 4		C9 H14 O4	203.11595	5.829
Level 4		C9 H14 O5	202.08424	13.896
Level 4		C9 H15 F O4	206.09465	10.595
Level 4		C9 H15 F3 N3 O P	269.09135	12.496
Level 4		C9 H15 F3 N6	264.13072	2.955
Level 4		C9 H15 F3 O2	212.10259	8.504
Level 4		C9 H15 F3 O2	212.10228	12.084
Level 4		C9 H15 F3 O3	228.09755	9.406
Level 4		C9 H15 F3 O5	260.0872	4.497
Level 4		C9 H15 F3 O5	260.08708	4.666
Level 4		C9 H15 N O2	169.11023	2.522
Level 4		C9 H15 N O3	185.1054	1.605
Level 4		C9 H15 N O3	185.10544	10.287
Level 4		C9 H15 N O3 S	217.07782	2.292
Level 4		C9 H15 N3 O	181.12175	10.071
Level 4		C9 H15 N3 O	181.12177	10.256
Level 4		C9 H15 N3 O2	197.1165	2.274
Level 4		C9 H15 N5	193.133	9.336
Level 4		C9 H15 N5 O2	225.12259	3.799
Level 4		C9 H16 Cl N5	229.10958	16.986
Level 4		C9 H16 F2 N2 O S	238.09539	8.025
Level 4		C9 H16 N10 O2 P2	358.09317	11.781
Level 4		C9 H16 N2 O2	184.12154	6.322
Level 4		C9 H16 N2 O2	184.12139	8.25
Level 4		C9 H16 O4	188.10478	7.408
Level 4		C9 H16 O5	204.09971	6.998
Level 4		C9 H16 O5	204.09966	7.281

Identification level	Name	Formula	Calc. MW	RT [min]
Level 4		C9 H16 O5	204.09966	7.601
Level 4		C9 H16 O5	204.09972	8.091
Level 4		C9 H17 F2 N S	209.10534	9.994
Level 4		C9 H17 F3 N3 P S	287.08288	10.504
Level 4		C9 H17 F3 N8 O S3	406.06327	8.631
Level 4		C9 H17 F3 O2	214.11782	9.895
Level 4		C9 H17 F3 O3	230.11287	8.135
Level 4		C9 H17 N O	155.13108	10.375
Level 4		C9 H17 N O2	153.11553	12.164
Level 4		C9 H17 N O2	153.1155	12.921
Level 4		C9 H17 N O3	187.1209	3.324
Level 4		C9 H17 N3 O	183.13735	9.451
Level 4		C9 H17 N4 O4 P S	308.06962	12.962
Level 4		C9 H17 N5 O	211.14348	11.233
Level 4		C9 H18 F2 O4 S2	292.06117	12.444
Level 4		C9 H18 F2 S	196.11006	18.202
Level 4		C9 H18 F5 N3 S	295.11439	13.969
Level 4		C9 H18 N O4 P	235.09761	14.863
Level 4		C9 H18 N2 O2	186.13673	1.748
Level 4		C9 H18 O3	174.12581	13.78
Level 4		C9 H18 O3	174.12565	9.38
Level 4		C9 H18 O3	174.12562	10.881
Level 4		C9 H18 O3	174.12557	11.076
Level 4		C9 H18 O3	174.12555	11.637
Level 4		C9 H18 O4	190.12044	7.853
Level 4		C9 H18 O4	190.12041	8.146
Level 4		C9 H18 O4	190.12041	8.276
Level 4		C9 H18 O4	190.12057	8.341
Level 4		C9 H18 O5	206.1153	7.764
Level 4		C9 H18 O5	206.11531	7.889
Level 4		C9 H18 O5	206.11535	8.125
Level 4		C9 H18 O5	206.11535	8.454
Level 4		C9 H18 O5	206.11535	8.728
Level 4		C9 H18 O5	206.1153	8.968
Level 4		C9 H18 O5	206.11531	9.109
Level 4		C9 H18 O5	206.1155	9.405
Level 4		C9 H18 O5	206.11533	10.875
Level 4		C9 H18 O7	238.10521	4.605
Level 4		C9 H19 F N P	191.12387	8.266
Level 4		C9 H19 F N P	191.12398	8.509
Level 4		C9 H19 F N3 P S	251.10215	6.289
Level 4		C9 H19 F3 O S	232.1102	16.097
Level 4		C9 H19 N O2	173.14183	10.534
Level 4		C9 H19 N O4	205.13134	1.802

Identification level	Name	Formula	Calc. MW	RT [min]
Level 4		C9 H19 N O4	205.13158	7.408
Level 4		C9 H19 N O5	221.12614	3.095
Level 4		C9 H19 N O6	237.12121	7.161
Level 4		C9 H19 N2 O2 Si	215.12151	9.578
Level 4		C9 H19 N3 O2	201.1478	8.57
Level 4		C9 H19 N9	253.17567	18.368
Level 4		C9 H2 F5 O3 P	283.96633	1.078
Level 4		C9 H2 N O3 P S2	266.92126	0.958
Level 4		C9 H20 Cl F O2 P2	276.06108	4.589
Level 4		C9 H20 F N2 O P	222.12959	7.265
Level 4		C9 H20 N O2 S	206.1209	1.396
Level 4		C9 H20 N2	156.16266	1.043
Level 4		C9 H20 O P2 S	238.07032	6.706
Level 4		C9 H20 O4	192.13615	8.695
Level 4		C9 H20 O4	192.13616	8.956
Level 4		C9 H20 O4	192.13633	9.102
Level 4		C9 H20 O4	192.13621	9.404
Level 4		C9 H20 O4	192.13624	9.566
Level 4		C9 H20 O4	192.13613	9.721
Level 4		C9 H20 O6	224.12585	5.482
Level 4		C9 H20 O6 P2	286.07364	8.402
Level 4		C9 H20 O6 P2	286.07358	8.512
Level 4		C9 H21 F N O4 P	257.11951	1.393
Level 4		C9 H21 F2 N O2 S	245.12605	1.85
Level 4		C9 H21 F2 N P2 S	275.08415	15.01
Level 4		C9 H21 F2 N S	213.1366	10.653
Level 4		C9 H21 F3 N2 O2 S	278.12679	11.121
Level 4		C9 H21 N O4	207.14704	2.568
Level 4		C9 H21 N O4	207.14705	8.391
Level 4		C9 H21 N O5	223.14214	7.879
Level 4		C9 H21 N O5	223.14189	8.707
Level 4		C9 H21 N O5	223.14196	9.111
Level 4		C9 H21 N O5	223.14214	9.406
Level 4		C9 H22 F3 N2 O2 P	278.13665	6.704
Level 4		C9 H22 F4 N4	262.17815	16.734
Level 4		C9 H22 N2 O2	190.16802	1.787
Level 4		C9 H22 N4 S3	282.10073	13.175
Level 4		C9 H23 F O P2 S	260.09246	12.07
Level 4		C9 H23 F O7 S	294.11539	4.651
Level 4		C9 H23 F2 N O S	231.14712	6.931
Level 4		C9 H23 F3 N O2 P	265.14257	5.504
Level 4		C9 H23 F3 N3 O P	277.15315	2.062
Level 4		C9 H23 F3 N3 O3 P	309.1436	7.128
Level 4		C9 H23 F3 N3 P	261.15761	5.916

Identification level	Name	Formula	Calc. MW	RT [min]
Level 4		C9 H23 F3 S	220.14649	17.274
Level 4		C9 H23 F4 N2 O2 P	298.14322	12.441
Level 4		C9 H23 N O4	209.16266	9.1
Level 4		C9 H23 N8 O P	290.17353	15.651
Level 4		C9 H24 F P S	214.13191	10.531
Level 4		C9 H24 F2 O S	218.15167	11.077
Level 4		C9 H24 F3 O5 P	300.13229	6.49
Level 4		C9 H24 N O2 P S2	273.09917	9.331
Level 4		C9 H25 F N2 O P2 S	290.11478	5.476
Level 4		C9 H25 F N2 S2	244.14417	14.712
Level 4		C9 H25 F2 N O S	233.16279	7.245
Level 4		C9 H25 F2 N O2 S	249.15748	6.992
Level 4		C9 H25 F2 N O3 S	265.15237	5.322
Level 4		C9 H25 N10 O P	320.1947	2.003
Level 4		C9 H26 Cl F2 N O2 S	285.13443	11.415
Level 4		C9 H26 F N4 O3 P	288.17266	17.305
Level 4		C9 H26 N10 O5	354.2087	13.006
Level 4		C9 H26 N5 O4 P S	331.14528	15.549
Level 4		C9 H26 N9 O P	307.19947	15.617
Level 4		C9 H27 F2 N O S	235.17821	8.145
Level 4		C9 H27 F2 N O S	235.17818	8.345
Level 4		C9 H27 F2 N O S	235.1782	8.489
Level 4		C9 H27 F2 N O2 S	251.17313	8.128
Level 4		C9 H27 F2 N O2 S	251.17313	8.726
Level 4		C9 H27 F3 N3 O2 P	297.18036	6.406
Level 4		C9 H27 F4 O2 P	274.16795	18.863
Level 4		C9 H27 F4 O4 P	306.15846	13.783
Level 4		C9 H28 F N O3 P2	279.15282	17.645
Level 4		C9 H28 F N5 O2 S3	353.13755	8.345
Level 4		C9 H28 N O P3 S	291.11095	7.262
Level 4		C9 H28 N8 O3 P2	358.17552	13.559
Level 4		C9 H29 F N6 O S3	352.15585	19.266
Level 4		C9 H29 F2 N O S	237.19388	8.687
Level 4		C9 H29 F2 N O S	237.19386	8.955
Level 4		C9 H29 F2 N O S	237.1939	9.408
Level 4		C9 H29 F5 N6 O4	380.21748	19.371
Level 4		C9 H29 F5 N8 S3	440.15985	15.624
Level 4		C9 H29 F6 N5 O S	369.19964	8.794
Level 4		C9 H29 F7 N P3	377.13897	10.034
Level 4		C9 H29 F8 N2 P	348.19351	15.302
Level 4		C9 H29 N5 O4 S	303.19493	6.29
Level 4		C9 H29 O7 P	280.16538	18.387
Level 4		C9 H3 Cl F3 N4 O P S5	465.82932	1.079
Level 4		C9 H3 Cl N2 O3 S5	381.8443	1.114

Identification level	Name	Formula	Calc. MW	RT [min]
Level 4		C9 H30 F2 N2 O5 P2	346.16038	15.869
Level 4		C9 H30 N8 O P2 S2	392.14451	11.324
Level 4		C9 H31 F N6 O2 S3	370.16624	19.269
Level 4		C9 H31 N3 O3 S3	325.15254	7.858
Level 4		C9 H31 N4 P S2	290.17327	13.38
Level 4		C9 H31 P3 S	264.136	13.229
Level 4		C9 H4 Cl O P S2	257.91286	0.944
Level 4		C9 H4 Cl2 F3 P S	301.91019	12.57
Level 4		C9 H4 F5 N3 O6	345.00168	1.287
Level 4		C9 H4 O4	176.01106	1.044
Level 4		C9 H5 F18 N6 P S	601.97559	17.256
Level 4		C9 H6 Cl3 N3 O9	404.91657	29.348
Level 4		C9 H6 I N O2 S2	350.88815	1.156
Level 4		C9 H7 N O	145.05289	5.845
Level 4		C9 H7 N O2	161.04779	5.912
Level 4		C9 H7 N O2	161.04768	9.432
Level 4		C9 H7 N O2	161.04787	9.736
Level 4		C9 H7 N O3	177.04251	2.765
Level 4		C9 H7 N O3	177.0428	10.238
Level 4		C9 H7 N5 O2	217.06012	8.211
Level 4		C9 H8 Br F3 N2 O	295.97698	13.561
Level 4		C9 H8 Br3 N O4	430.8011	21.018
Level 4		C9 H8 Cl N6 O P S	313.99089	5.636
Level 4		C9 H8 N2 O	160.06376	8.672
Level 4		C9 H8 N2 O2	176.05861	2.237
Level 4		C9 H8 O4	180.04215	19.389
Level 4		C9 H9 N O	147.06851	10.589
Level 4		C9 H9 N O S	179.04065	9.624
Level 4		C9 H9 N O2	163.06343	9.041
Level 4		C9 H9 N O2	163.06343	9.125
Level 4		C9 H9 N O2	163.06345	9.358
Level 4		C9 H9 N O2	163.06324	11.667
Level 4		C9 H9 N O2	163.06342	11.921
Level 4		C9 H9 N O3	179.0582	1.742
Level 4		C9 H9 N O3	179.05818	2.23
Level 4		C9 H9 N3 O2	191.06951	11.347
Level 4		C9 H9 N3 O2	191.06951	12.465
Level 4		C9 H9 N3 O3	207.06451	7.258
Level 4		Ce F4	215.89929	1.111
Level 4		Eu S	184.89301	0.957
Level 4		F P S2	113.91608	1.089
Level 4		F4 O S	123.96034	0.956
Level 4		F4 O S	123.9603	1.104
Level 4		F9 P Si3	285.89039	1.131

Identification level	Name	Formula	Calc. MW	RT [min]
Level 4		H2 O5 Sn2	321.79527	1.162
Level 4		H3 O3 Sc	95.96418	0.937
Level 4		H3 O3 Sc	95.9642	0.986
Level 4		H4 Cl N2 P S	129.95229	1.015
Level 4		H4 Cl N2 P S	129.95238	1.172
Level 4		I3 Tl	585.68932	15.574
Level 4		Mg O4 S	119.93711	0.953
Level 5			235.65265	1.526
Level 5			159.12434	8.345
Level 5			246.59172	9.26
Level 5			650.42058	12.121
Level 5			546.65807	15.06
Level 5			627.52477	17.706
Level 5			569.73015	18.054
Level 5			586.49123	18.098
Level 5			1110.72385	18.623
Level 5			1230.91233	19.396
Level 5			688.48033	19.66
Level 5			673.49509	20.024
Level 5			408.76957	20.333
Level 5			907.76836	24.354
Level 5			894.76732	25.419
Level 5			868.74932	26.502
Level 5			868.74986	26.702
Level 5			851.79503	28.842
Level 5			889.81212	29.136
Level 5			119.04889	29.339
Level 5			145.02269	29.351
Level 5			104.01277	0.898
Level 5			517.74925	0.903
Level 5			90.01665	0.903
Level 5			201.97267	0.905
Level 5			471.75823	0.908
Level 5			207.9117	0.91
Level 5			343.81769	0.92
Level 5			120.97805	0.922
Level 5			103.01186	0.922
Level 5			227.87751	0.933
Level 5			132.01009	0.935
Level 5			373.80489	0.938
Level 5			421.78115	0.939
Level 5			132.00766	0.94
Level 5			145.02248	0.94
Level 5			146.98437	0.941

Identification level	Name	Formula	Calc. MW	RT [min]
Level 5			131.00683	0.941
Level 5			133.0281	0.942
Level 5			147.96839	0.942
Level 5			125.99334	0.943
Level 5			161.98398	0.943
Level 5			128.97383	0.944
Level 5			129.95778	0.944
Level 5			116.98181	0.944
Level 5			133.03057	0.944
Level 5			133.91496	0.945
Level 5			103.96715	0.945
Level 5			83.95231	0.945
Level 5			112.99618	0.945
Level 5			154.96613	0.946
Level 5			119.01239	0.946
Level 5			85.98538	0.947
Level 5			86.98619	0.947
Level 5			101.00182	0.947
Level 5			107.98276	0.948
Level 5			105.95579	0.948
Level 5			142.98948	0.948
Level 5			100.00106	0.949
Level 5			118.01158	0.949
Level 5			103.99593	0.949
Level 5			111.97767	0.949
Level 5			121.97767	0.95
Level 5			104.99671	0.95
Level 5			133.98907	0.95
Level 5			115.97855	0.95
Level 5			137.98407	0.95
Level 5			87.94521	0.95
Level 5			507.704	0.951
Level 5			119.97344	0.951
Level 5			93.9671	0.951
Level 5			84.95565	0.951
Level 5			453.69719	0.952
Level 5			339.80562	0.953
Level 5			127.0119	0.953
Level 5			231.90669	0.953
Level 5			132.99208	0.953
Level 5			91.97864	0.955
Level 5			102.96619	0.955
Level 5			138.99665	0.955
Level 5			136.9637	0.958

Identification level	Name	Formula	Calc. MW	RT [min]
Level 5			223.88899	0.96
Level 5			109.97988	0.961
Level 5			123.00729	0.961
Level 5			154.97418	0.961
Level 5			118.95332	0.963
Level 5			109.94469	0.963
Level 5			120.98591	0.964
Level 5			127.95524	0.964
Level 5			150.97958	0.968
Level 5			386.79949	0.969
Level 5			253.84409	0.974
Level 5			109.95282	0.974
Level 5			211.86268	0.975
Level 5			100.94264	0.975
Level 5			195.86829	0.976
Level 5			489.7224	0.977
Level 5			521.68579	0.977
Level 5			91.93412	0.98
Level 5			378.81835	0.983
Level 5			387.78512	0.984
Level 5			353.88219	0.984
Level 5			345.79323	0.986
Level 5			553.71709	0.986
Level 5			577.70354	0.987
Level 5			455.80068	0.989
Level 5			483.79079	0.99
Level 5			87.98373	0.997
Level 5			467.76775	0.997
Level 5			118.94499	1
Level 5			407.80196	1
Level 5			413.80712	1
Level 5			653.64433	1.003
Level 5			164.12438	1.007
Level 5			459.76784	1.008
Level 5			528.78349	1.008
Level 5			97.96803	1.008
Level 5			207.91151	1.011
Level 5			471.7594	1.013
Level 5			353.74794	1.013
Level 5			155.96746	1.016
Level 5			463.72695	1.018
Level 5			361.76122	1.018
Level 5			345.79262	1.019
Level 5			491.72066	1.019

Identification level	Name	Formula	Calc. MW	RT [min]
Level 5			605.71843	1.021
Level 5			113.8854	1.025
Level 5			129.98799	1.025
Level 5			114.01663	1.025
Level 5			95.91444	1.025
Level 5			445.808	1.027
Level 5			129.9942	1.028
Level 5			575.77604	1.03
Level 5			138.90739	1.03
Level 5			122.93344	1.03
Level 5			505.76915	1.031
Level 5			395.82746	1.031
Level 5			303.80373	1.033
Level 5			673.77503	1.035
Level 5			372.83101	1.036
Level 5			461.77303	1.036
Level 5			497.75412	1.036
Level 5			372.82045	1.038
Level 5			309.83369	1.038
Level 5			667.75981	1.041
Level 5			77.94832	1.044
Level 5			563.80496	1.044
Level 5			162.02037	1.045
Level 5			585.80705	1.045
Level 5			203.90354	1.046
Level 5			719.69475	1.047
Level 5			621.76901	1.05
Level 5			469.80355	1.051
Level 5			157.92948	1.051
Level 5			252.89104	1.051
Level 5			83.95233	1.051
Level 5			290.88768	1.053
Level 5			461.82555	1.054
Level 5			141.97096	1.055
Level 5			492.82071	1.055
Level 5			122.0428	1.056
Level 5			197.8775	1.058
Level 5			583.79134	1.059
Level 5			208.92249	1.061
Level 5			286.84403	1.063
Level 5			517.74493	1.063
Level 5			278.81178	1.064
Level 5			391.77994	1.064
Level 5			567.73906	1.065

Identification level	Name	Formula	Calc. MW	RT [min]
Level 5			479.77245	1.065
Level 5			209.84846	1.065
Level 5			637.74587	1.071
Level 5			95.94998	1.072
Level 5			579.79045	1.073
Level 5			251.85573	1.08
Level 5			236.86729	1.082
Level 5			622.77971	1.086
Level 5			139.91876	1.09
Level 5			347.78755	1.09
Level 5			559.73052	1.092
Level 5			575.77574	1.093
Level 5			543.75481	1.093
Level 5			573.77337	1.094
Level 5			131.92668	1.096
Level 5			391.79699	1.1
Level 5			389.8774	1.104
Level 5			143.9214	1.105
Level 5			151.88982	1.105
Level 5			587.80419	1.111
Level 5			569.76096	1.114
Level 5			554.79287	1.114
Level 5			615.75538	1.119
Level 5			106.02548	1.121
Level 5			141.89335	1.122
Level 5			585.80705	1.123
Level 5			154.97423	1.13
Level 5			109.9447	1.131
Level 5			569.76451	1.132
Level 5			497.76198	1.132
Level 5			381.84685	1.133
Level 5			136.96372	1.133
Level 5			275.81001	1.138
Level 5			519.74272	1.147
Level 5			118.98941	1.155
Level 5			112.99627	1.167
Level 5			132.00761	1.168
Level 5			206.92664	1.171
Level 5			379.75371	1.172
Level 5			131.00683	1.175
Level 5			641.76018	1.179
Level 5			146.98434	1.185
Level 5			127.06222	1.218
Level 5			599.77672	1.25

Identification level	Name	Formula	Calc. MW	RT [min]
Level 5			148.00484	1.251
Level 5			595.76341	1.262
Level 5			176.12409	1.274
Level 5			134.09503	5.808
Level 5			191.63954	7.923

Figure S2: Comparison of the node and sample signatures

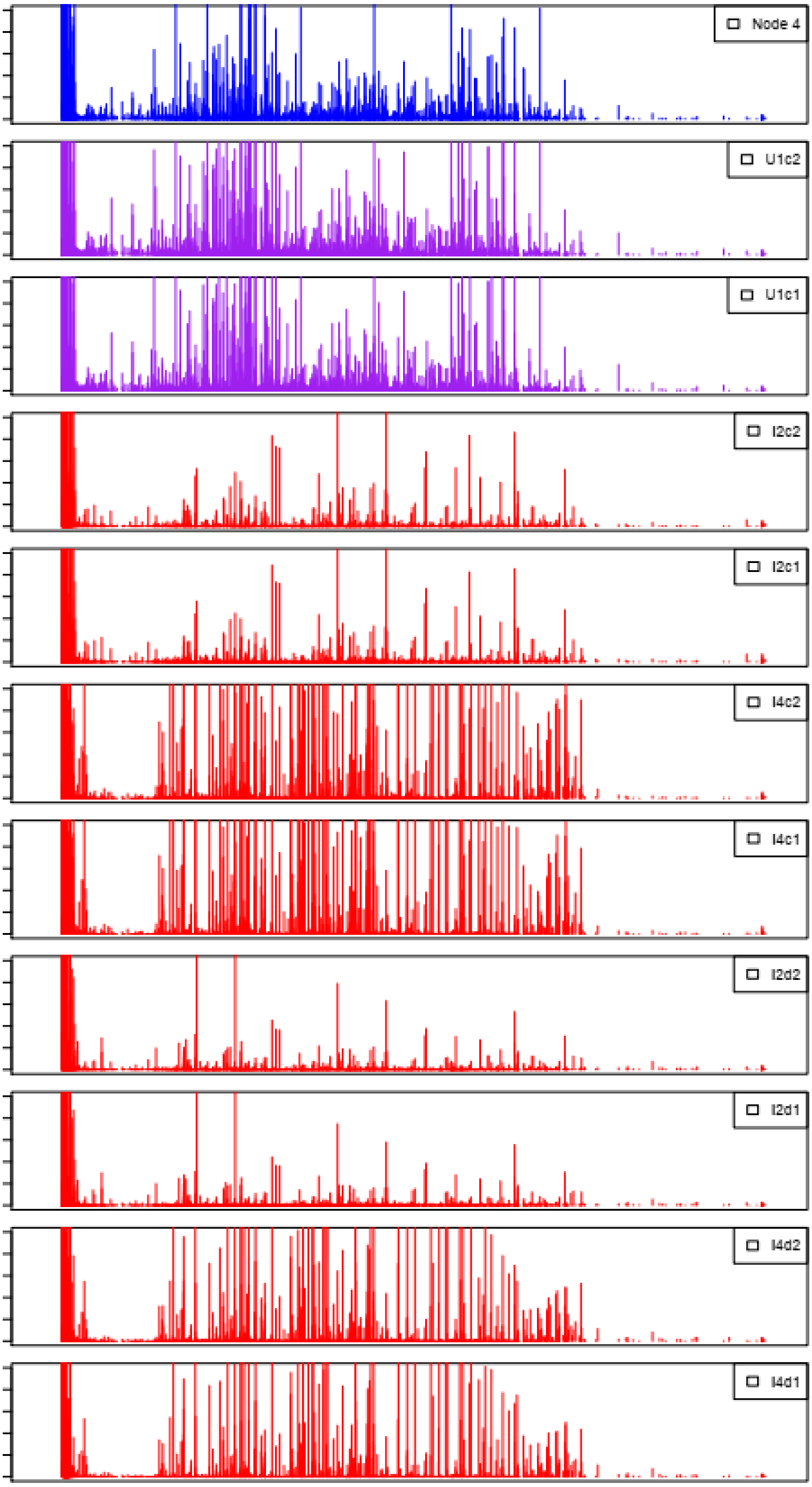

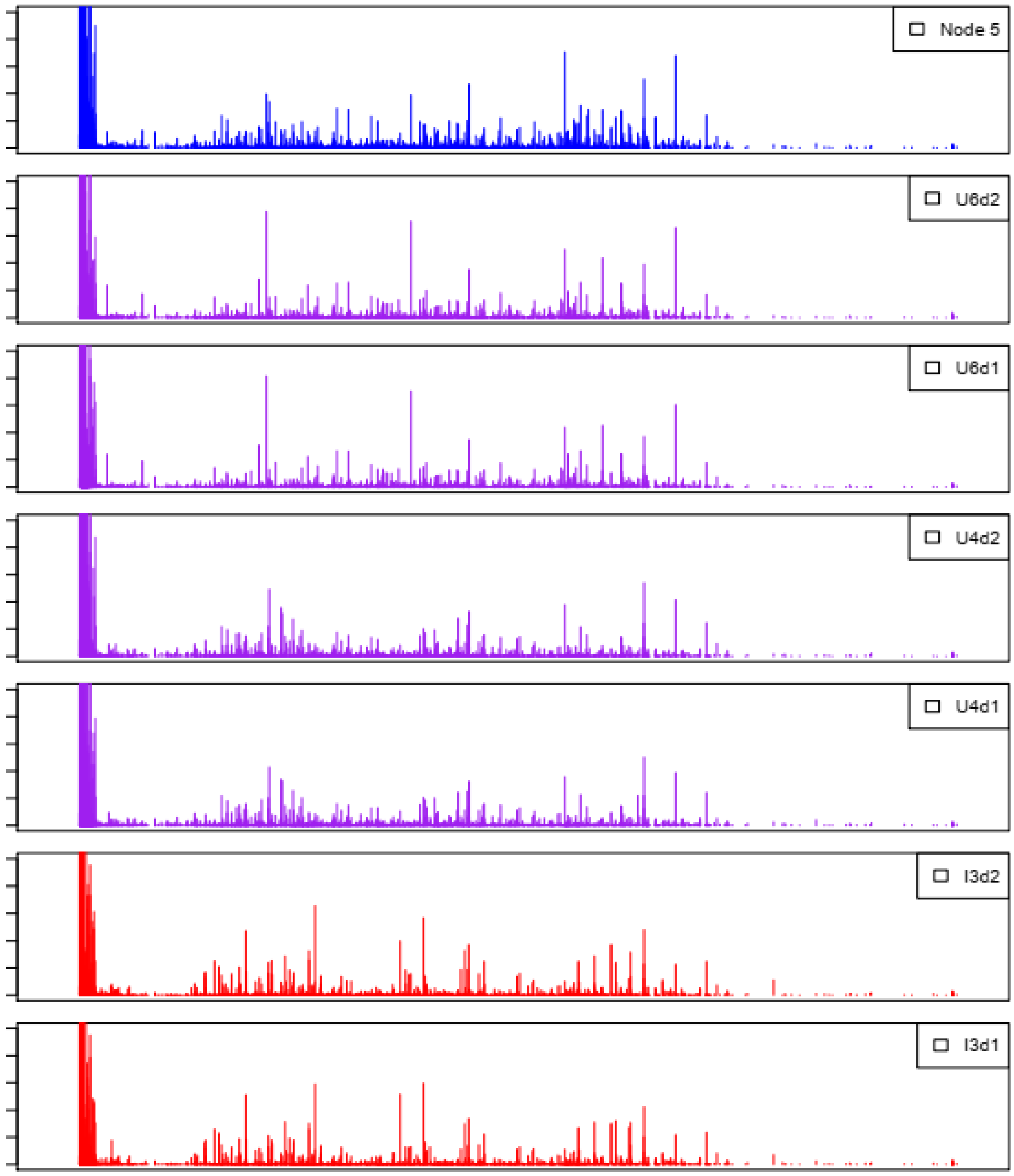
Node 5
U6d2
U6d1
U4d2
U4d1
I3d2
I3d1

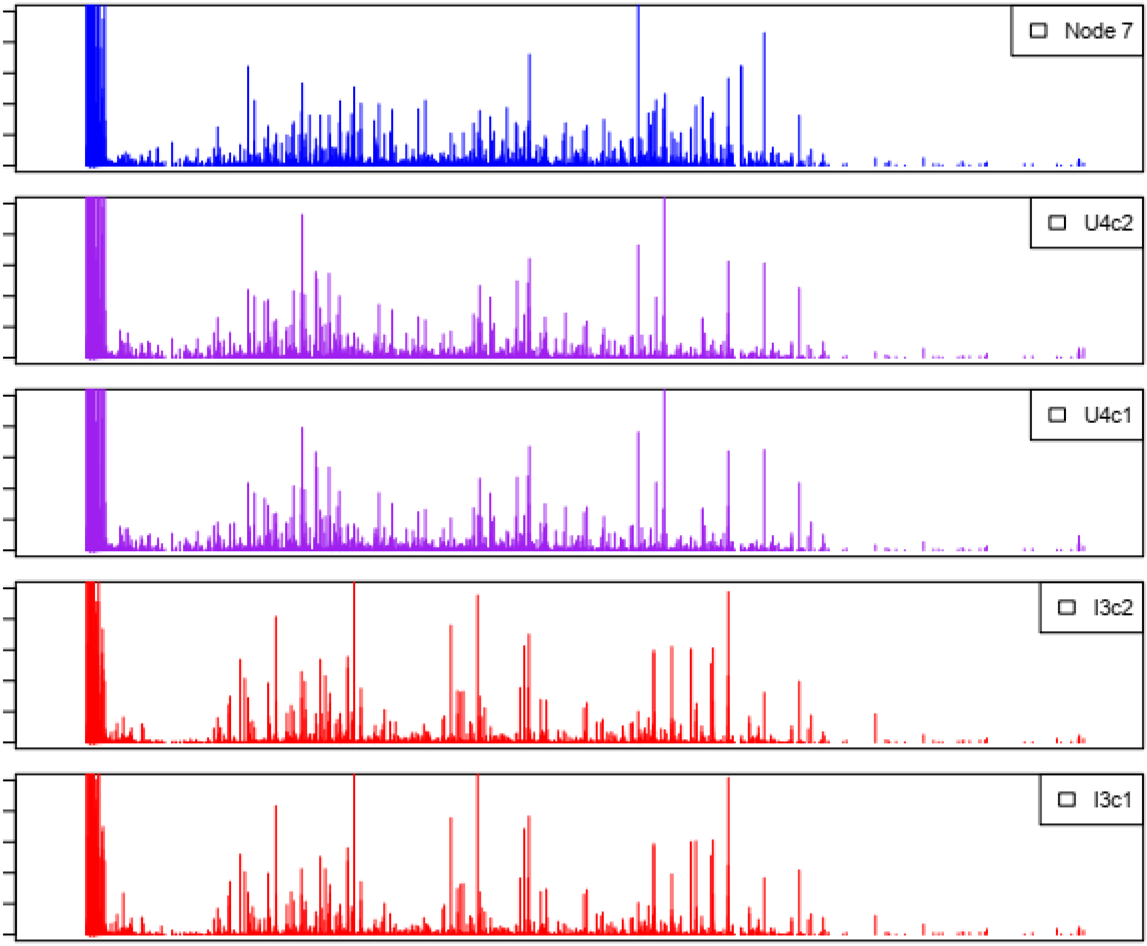
Node 7
U4c2
U4c1
I3c2
I3c1
